# Largest known madtsoiid snake from warm Eocene period of India suggests intercontinental Gondwana dispersal

**DOI:** 10.1038/s41598-024-58377-0

**Published:** 2024-04-18

**Authors:** Debajit Datta, Sunil Bajpai

**Affiliations:** Department of Earth Sciences, Indian Institute of Technology Roorkee, Roorkee, Uttarakhand 247667 India

**Keywords:** Palaeontology, Palaeontology

## Abstract

Here we report the discovery of fossils representing partial vertebral column of a giant madtsoiid snake from an early Middle Eocene (Lutetian, ~ 47 Ma) lignite-bearing succession in Kutch, western India. The estimated body length of ~ 11–15 m makes this new taxon (*Vasuki indicus* gen et sp. nov.) the largest known madtsoiid snake, which thrived during a warm geological interval with average temperatures estimated at ~ 28 °C. Phylogenetically, *Vasuki* forms a distinct clade with the Indian Late Cretaceous taxon *Madtsoia pisdurensis* and the North African Late Eocene *Gigantophis garstini*. Biogeographic considerations, seen in conjunction with its inter-relationship with other Indian and North African madtsoiids, suggest that *Vasuki* represents a relic lineage that originated in India. Subsequent India-Asia collision at ~ 50 Ma led to intercontinental dispersal of this lineage from the subcontinent into North Africa through southern Eurasia.

## Introduction

Madtsoiidae are an extinct clade of primarily Gondwanan terrestrial snakes with a temporal range spanning about 100 Myr from the Late Cretaceous–Late Pleistocene^1–3^. Their geographic range during the Late Cretaceous encompassed Madagascar, South America, India, Africa and the European archipelago^1,4–9^. The Cenozoic forms are restricted to North Africa, South America, the Indian subcontinent and Australia^2,10–17^. Madtsoiids display a broad spectrum of body-sizes and include some of the largest known terrestrial snakes that ever lived^2,7,9^. Although a speciose clade, most taxa are known exclusively from vertebrae, resulting in poorly constrained in-group relationships^2,8,16^. Additionally, the phylogenetic position of Madtsoiidae within Ophidia has remained contentious, as some studies recover it within Serpentes whereas others place it outside the crown group^3,9,17–20^. These phylogenetic uncertainties have hampered our understanding of madtsoiid biogeography and radiation events^2,8^.

**Figure 1:**
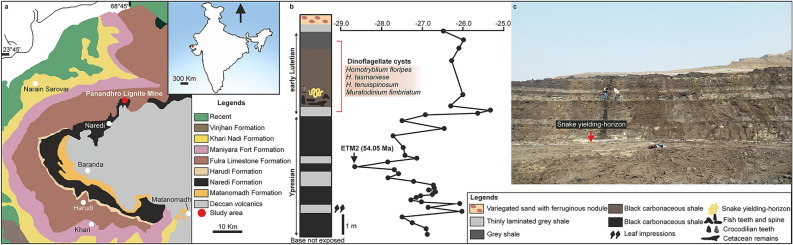
Geological map of Kutch Basin showing fossil locality (**a**); stratigraphic column at Panandhro Lignite Mine showing the position of madtsoiid snake-yielding horizon with age diagnostic dinoflagellate cyst assemblage and δ^13^C curve marking hyperthermal event ETM2 (modified after Agrawal et al.^23^) (**b**); panoramic view of the fossil site (**c**). Map and stratigraphic column were drawn by D.D. using CorelDRAW 2019 (Version number: 21.0.0.593, URL link: http://www.corel.com/en/). ETM2 age estimate after Westerhold et al.^24^.

In the Indian subcontinent, Late Cretaceous (Maastrichtian) madtsoiids are known from the Deccan volcanic province, including the large-sized *Madtsoia pisdurensis* from the Lameta Formation^6,8^. Among Tertiary madtsoiids, indeterminate forms are known from the early Paleocene Khadro Formation (Pakistan^16^) and the early Eocene Cambay Shale (India^15^). The latter also yielded the large madtsoiid *Platyspondylophis*^21^*.* The Eocene and Late Oligocene records include indeterminate taxa from Kutch and Ladakh, respectively^14,22^. Here we report the discovery of a giant madtsoiid snake, one of largest snakes ever reported, from an interval corresponding to a warm Middle Eocene period (~ 47 Ma) of India. Fossils were collected from an early Lutetian grey shale unit from Panandhro Lignite Mine, Kutch, Gujrat State, western India (Supplementary Note 1, Fig. 1), and includes an excellently preserved, partial vertebral column. The discovery of a giant Eocene snake has important implications for madtsoiid biogeography in the context of Gondwanan inter-continental dispersal, and the evolution of large body-sizes possibly driven by high temperatures in the Middle Eocene tropical zones.

## Results

### Systematic paleontology

Squamata Oppel, 1811

Ophidia Brongniart, 1800

Madtsoiidae (Hoffstetter 1961) McDowell, 1987

*Vasuki indicus* gen. et sp. nov.

#### Etymology

Generic name after the well-known Hindu mythical serpent ‘Vāsuki’ around the neck of Lord Shiva; specific name is for the country of origin i.e., India.

#### Holotype

**Figure 2:**
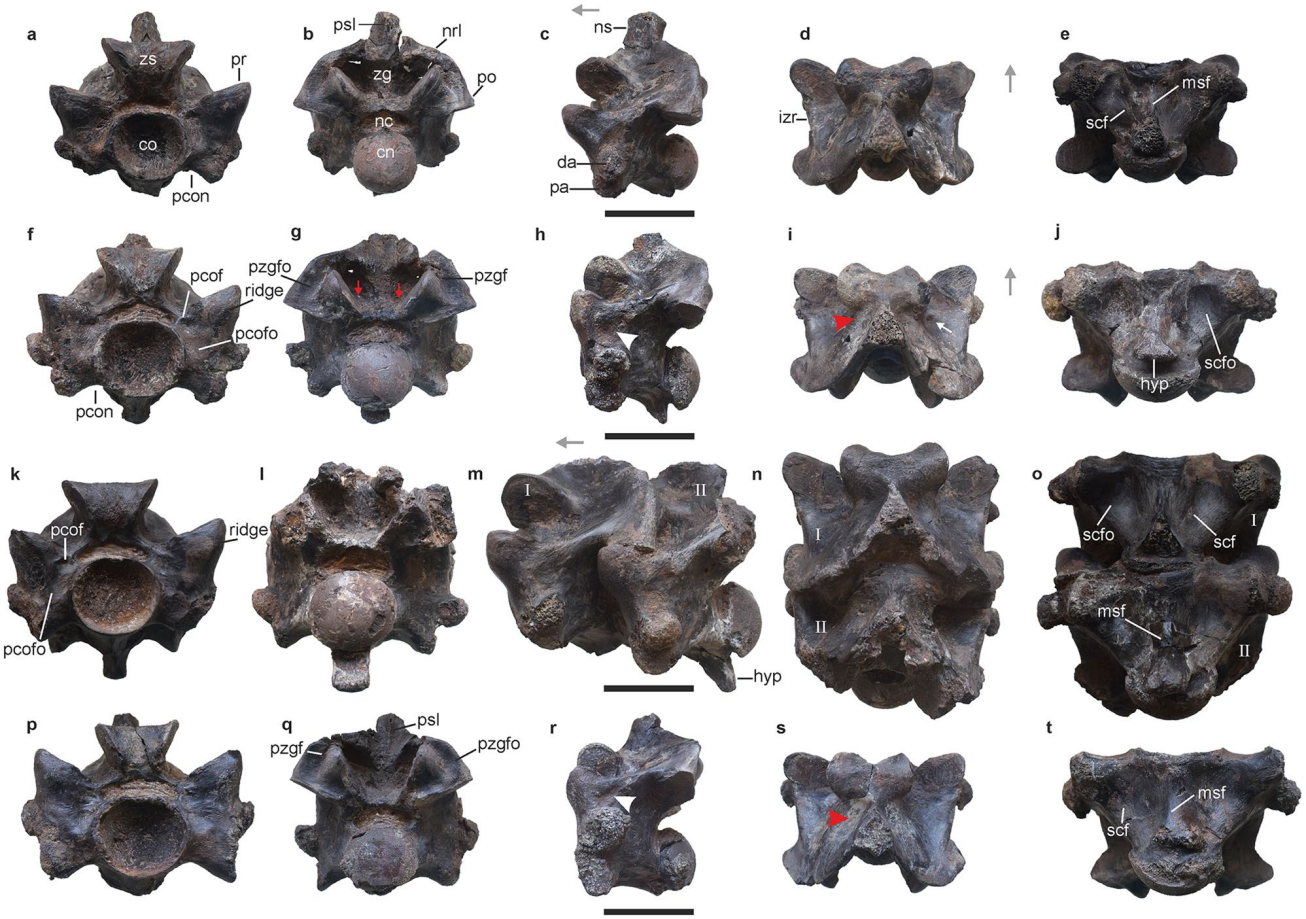
Anterior trunk vertebrae of *Vasuki indicus*. IITR/VPL/SB 3102-3, partial vertebra in anterior view (**a**); posterior view (**b**); left lateral view (**c**); dorsal view (**d**); ventral view (**e**). IITR/VPL/SB 3102-5, complete vertebra in anterior view (**f**); posterior view (**g**); left lateral view (**h**); dorsal view (**i**); ventral view (**j**). IITR/VPL/SB 3102-7I-II, partial vertebra in anterior view (**k**); posterior view; (**l**); left lateral view (**m**); dorsal view (**n**); ventral view (**o**). IITR/VPL/SB 3102-6, complete posterior anterior trunk vertebra in anterior view (**p**); posterior view (**q**); left lateral view (**r**); dorsal view (**s**); ventral view (**t**). Grey arrows indicate anterior direction. Red arrowheads and arrows indicate fossae on neural spinal base and endozygantral foramina, respectively. Roman numerals on figures (**m–o**) refer to individual vertebrae in articulated specimens where ‘I” is towards the anterior. White arrowhead and arrow indicate fossa medial to diapophysis and foramen on dorsal surface of neural arch. *co* cotyle, *cn* condyle, *da* diapophysis, *hyp* hypapophysis, *izr* interzygapophyseal ridge, *msf* median shaft, *nc* neural canal, *nrl* neural arch lamina, *ns* neural spine, *pa* parapophysis, *pcof* paracotylar foramen, *pcofo* paracotylar fossa, *pcon* paracotylar notch, *po* postzygapophysis, *pr* prezygapophysis, *psl* prespinal lamina, *pzgf* parazygantral foramen, *pzgfo* parazygantral fossa, *scf* subcentral foramen, *scfo* subcentral fossa, *zg* zygantrum, *zs* zygosphene. Scale bar represents 50 mm.

**Figure 3:**
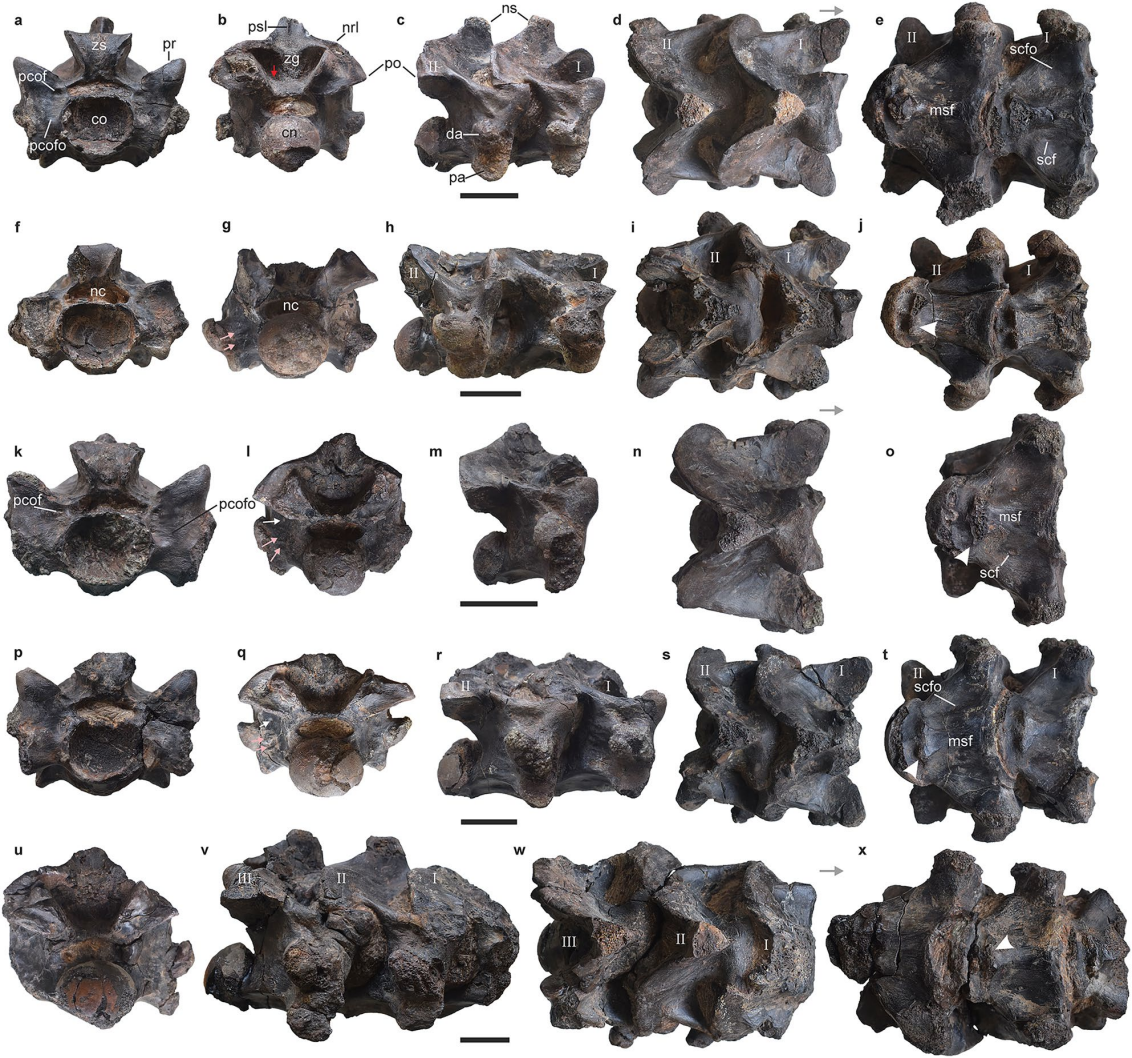
Precloacal vertebrae of *Vasuki indicus*. IITR/VPL/SB 3102-10I-II, complete posterior anterior trunk/mid-trunk vertebrae in anterior view (**a**); posterior view (**b**); right lateral view (**c**); dorsal view (**d**); ventral view (**e**). IITR/VPL/SB 3102-9I-II, partial mid-trunk vertebrae in anterior view (**f**); posterior view (**g**); left lateral (reversed) view (**h**); dorsal view (**i**); ventral view (**j**). IITR/VPL/SB 3102-4, nearly-complete mid-trunk vertebra in anterior view (**k**); posterior view; (**l**); left lateral (reversed) view (**m**); dorsal view (**n**); ventral view (**o**). IITR/VPL/SB 3102-8I-II, partial mid-trunk vertebrae in anterior view (**p**); posterior view (**q**); right lateral view (**r**); dorsal view (**s**); ventral view (**t**). IITR/VPL/SB 3102-11I-III, partial mid-trunk vertebrae in posterior view (**u**); right lateral view (**v**); dorsal view (**w**); ventral view (**x**). Grey arrows indicate anterior direction. Roman numerals on figures (**c–e,h–j,r–t,v–w**) refer to individual vertebrae in articulated specimens where ‘I” is towards the anterior. Pink and white arrows indicate fossae and foramen on lateral surface of centrum, respectively. Red arrow indicates endozygantral foramen. White arrowheads indicate paired protuberance on ventral median shaft. *co* cotyle, *cn* condyle, *da* diapophysis, *hyp* hypapophysis, *izr* interzygapophyseal ridge, *msf* median shaft, *nc* neural canal, *nrl* neural arch lamina, *ns* neural spine, *pa* parapophysis, *pcof* paracotylar foramen, *pcofo* paracotylar fossa, *po* post-zygapophysis, *pr* prezygapophysis, *psl* prespinal lamina, *scf* subcentral foramen, *scfo* subcentral fossa, *zg* zygantrum, *zs* zygosphene. Scale bar represents 50 mm.

IITR/VPL/SB 3102-1-21; a partial vertebral column representing the precloacal region (Figs. 2, 3; Supplementary Table 1).

#### Horizon and locality

Naredi Formation; Panandhro Lignite Mine, district Kutch, Gujarat state, western India.

#### Diagnosis

*Vasuki* exhibits a unique combination of the following characters: presence of prominent paracotylar foramina (shared with Madtsoiidae); middle-sized cotyle (shared with Madtsoiidae); median prominence on ventral margin of centrum (shared with Madtsoiidae); prezygapophyseal process absent; high angle of synapophysis with horizontal in anterior view (avg. 71.5°); MTV diapophysis level with dorsoventral midpoint of neural canal (shared with *Madtsoia madagascariensis*, *Madtsoia camposi*, *Wonambi barriei* and *Adinophis*); prezygapophyseal buttress succeeded posteriorly by elliptical fossa (shared with *Madtsoia pisdurensis*); deep V-shaped embayment (shared with *Gigantophis garstini* and *Madtsoia pisdurensis*); oval precloacal cotyle (shared with *Gigantophis garstini* and *Madtsoia pisdurensis*); transversely wide vertebrae (shared with *Gigantophis garstini* and *Madtsoia pisdurensis*); neural spine posteriorly canted (shared with *Gigantophis garstini* and *Madtsoia pisdurensis*); broad hemal keel with posterior process (shared with *Gigantophis garstini* and *Madtsoia pisdurensis*); strongly notched anterior zygosphenal margin; endozygantral foramen present (shared with *Madtsoia madagascariensis*, *Powellophis* and *Gigantophis garstini*).

Autapomorphies: exceptionally large vertebrae [centrum length (cL): 37.5–62.7 mm and prezygapophyseal width (prW): 62.4–111.4 mm]; neural spine cross-section spade-shaped; poorly developed hemal keel which remains dorsal to the parapophyses; chisel-shaped posterior process of the hemal keel.

### Description

The collection comprises 27 associated vertebrae which are mostly well-preserved and include a few in articulation (Figs. 2A–T, 3A–W). 22 out of the 27 specimens can be confidently assigned to the precloacal region based on the absence of hemapophyses, pleurapophyses and lymphapophyses, and are further constrained to a position anterior to the posterior trunk region as suggested by a greater mediolateral width of the neural arch compared to centrum length (sensu LaDuke^1^; Rio and Mannion^2^; Supplementary Tables 1, 2; Supplementary Fig. 2). Such vertebral dimensions are usually found in large-bodied madtsoiids such as, *Gigantophis*^2^; *Yurlunggur*^11^, *Madtsoia*^1,10,13^, and *Wonambi*^25^. Moreover, the closure of vertebral sutures suggests these specimens likely reached skeletal maturity, similar for instance to *Madtsoia pisdurensis*^8^.

*Vasuki* is characterized by exceptionally large vertebrae where centrum length (cL) and prezygapophyseal width (prW) range between 37.5–62.7 and 62.4–111.4 mm, respectively (Supplementary Table 2). We recognize this as an autapomorphy since these proportions eclipse all large-sized madtsoiids [*Madtsoia* (cL = 18–25 mm; prW = 35–65 mm; LaDuke et al.^1^), *Gigantophis* (cL = 28–41 mm; prW = 44–66 mm; Rio and Mannion^2^), *Platyspondylophis* (cL = 18–21 mm; prW = 26–43 mm; Smith et al.^21^) and *Yurlunggur* (cL = 15–22 mm; prW = 19–41 mm)]. Some caution, however, is warranted here because of uncertainties as to whether the largest size of these large-bodied madtsoiids has been captured, although, the same is true for *Vasuki.*

In overall form, the vertebrae of the new Indian taxon are massive (prW >> cL) and comprise a procoelous centrum. Anteriorly, the centrum preserves an anteroventrally inclined cotyle, whereas the posterior condyle is deflected posterodorsally resulting in considerable visibility of the condyle and cotyle in dorsal and ventral views, respectively (Fig. 2C,E). In anterior view, the cotyle is strongly concave with its ventral margin recessed relative to the dorsal. The cotyle is mediolaterally wider than dorsoventrally high (Figs. 2P, 3A,F,K; IITR/VPL/SB 3102-4, coW/coH = 1.2; Supplementary Table 2) as in all madtsoiids [e.g., *Gigantophis garstini*^2^ (NHMUK R8344, coW/coH = 1.2), *Madtsoia madagascariensis* (FMNH PR 2551, coW/coH = 1.24) *Yurlunggur* (NTM P8695-243, coW/coH = 1.22), and *Wonambi* (QMF23038, coW/coH = 1.4]. Laterally, the cotyle is bordered on each side by a well-developed and moderately deep paracotylar fossa (Figs. 2K,P, 3A,K). The dorsal and ventral margins of the fossa are prominent and defined by bony struts emanating from the dorsolateral and lateral cotylar margins, respectively. The lateral margin of the fossa, however, is flush with the surface. Furthermore, in some specimens the paracotylar fossa is divided into a shallower dorsal and deeper ventral sub-fossa by a weak secondary strut extending laterally from the dorsolateral margin of the cotyle. A tiny paracotylar foramen is present on the dorsal-most part of one or both paracotylar fossae, immediately lateral to the neural canal (Figs. 2F,K,P, 3A,K). While the presence of paracotylar fossae and foramina is a synapomorphy of Madtsoiidae^16,26^, the exact morphology of these features is variable across the clade. “*Gigantophis* sp.” (CPAG-RANKT-V-1), *Menarana nosymena* and *Adinophis fisaka* (FMNH PR 2572) differ from *Vasuki* in the presence of paired paracotylar foramina on each side^1,16,27^. In *Madtsoia* and *Eomadtsoia* (MPEF-PV 2378) the foramina are deep and comparatively large, whereas in *Yurlunggur* these occur in clusters^7,8,10,11,13^. *Eomadtsoia*, however, shares with *Vasuki* the presence of prominent ventral rim of the paracotylar fossa^7^. In *Gigantophis garstini* the paracotylar fossa lacks a ventral margin and in *Platyspondylophis* the paracotylar foramen is absent altogether^2,21^.

The posterior condyle is transversely wider than high (IITR/VPL/SB 3102–4, cnW/cnH = 1.2; Supplementary Table 2) with the width progressively increasing from ATV (Fig. 2B,G; cnW/cnH = 1.1) to MTV (Fig. 3G, Q; cnW/cnH = 1.2–1.3). Similar proportions of the posterior condyle characterize most madtsoiids [e.g., *Nidophis* (LPB FGGUB v.547/3, ATV, cnW/cnH = 1.1; LPB FGGUB v.547/1, MTV, cnW/cnH = 1.2); *Gigantophis garstini* (NHMUK R8344, MTV, cnW/cnH = 1.2 Rio and Mannio^2^); *Madtsoia camposi* (DGM 1310b, MTV, cnW/cnH = 1.3] (Fig. 3G,I,Q). Furthermore, in posterior view, two small, distinct fossae are discernible on the lateral surface of the centrum immediately posterior to the left diapophysis (Fig. 3G,I,Q). The fossae are vertically arranged, on top of each other, and separated by a prominent ridge. Whether these unilateral fossae represent an individual condition or a general feature cannot be currently ascertained and will require additional specimens of *Vasuki*.

The synapophysis is dorsoventrally high and comprises a distinct diapophysis and parapophysis (Figs. 2M,R, 3C,R) unlike in *Gigantophis garstini*, *Madtsoia madagascariensis*, and *Madtsoia pisdurensis*^1,2,8^. In anterior view, the orientation of the synapophysis changes from ventrolateral (Fig. 2F,K) to somewhat laterally facing (Fig. 3K,P,U) across the precloacal series. This change is marked by an increase in the synapophyseal angle (α), with the horizontal, from ATV (α = avg. 56.6°) to MTV (α = avg. 71.5°). A narrower synapophyseal angle was observed in most of the comparative madtsoiid taxa including *Eomadtsoia* [MPEF-PV 2378 (MTV), α = 45°], *Gigantophis garstini* [NHMUK R8344 (MTV) α = 48], *Madtsoia madagascariensis* [FMNH PR 2549 (ATV), α = 47°; FMNH PR 2551 (MTV), α = 56°], “*Gigantophis* sp.” [CPAG-RANKT-V-1 (MTV), α = 56°], *Madtsoia camposi* [DGM 1310c (MTV), α = 57°], *Wonambi* [QMF23038 (MTV) α = 58°] and *Madtsoia bai* [AMNH 3155 (MTV), α = 62°]. In lateral view, the synapophysis is inclined at (β) 20°–27° from the vertical in *Vasuki*. This is similar to *Wonambi* [QMF23038, β = 25°], *Nanowana* [QMF19741, β =  ~ 25°], *Madtsoia camposi* [DGM 1310c, β = 26°] and *Yurlunggur* [P8695, β = 22°–26°]. In contrast, wider angles characterize *Gigantophis garstini* [NHMUK R8344, β = 30°^2^], *Platyspondylophis* [β = 30°–35°], *Madtsoia madagascariensis* [FMNH PR 2549, β = 33°] and “*Gigantophis* sp.” [CPAG-RANKT-V-1, β =  ~ 90°], whereas in *Patagniophis* [β = 7°–9°], *Powellophis* [PVL 4714–4, β = 18°] and *Madtsoia pisdurensis* [225/GSI/PAL/CR/10, β = 12°] the angles are narrower.

An arcuate paracotylar notch (sensu LaDuke et al.^1^), between the ventral cotylar rim and the parapophysis, is consistently present in all specimens (Fig. 2A,F). The parapophysis comprises a sub-rectangular facet, in lateral view, and extends below the ventral cotylar rim in ATV (Fig. 2F,H,P,R). In MTV it lies dorsal to the ventral cotylar rim (Fig. 3F,P) unlike *Madtsoia pisdurensis* and *Gigantophis garstini* where the parapophyseal base is ventral and in level with the ventral cotylar rim, respectively^2,8^. The diapophysis is bulbous and extends laterally beyond the prezygapophysis (Figs. 2F,H, 3P,R), contrary to *Powellophis*^3^, *Patagoniophis australiensis*^28^, *Madtsoia pisdurensis*^8^, *Madtsoia madagascariensis*^1^ and *Nidophis*^9^. The dorsal margin of the diapophysis remains ventral to the dorsal cotylar margin in ATV (Fig. 2A,F), but becomes level with the dorsoventral midpoint of the neural canal in MTV (Fig. 3K,P). A similar disposition of the MTV diapophysis is observed in *Madtsoia madagascariensis*, *Madtsoia camposi*, *Wonambi barriei* and *Adinophis*^1,2,13,27^. The dorsal diapophyseal margin lies between the ventral margin of the neural canal and the dorsoventral midpoint of the cotyle in “*Gigantophis* sp.”^16^, *Gigantophis garstini*^2^, *Nidophis*^9^, *Yurlunggur*^11^ and *Powellophis*^3^. In *Platyspondylophis* the diapophysis extends beyond the ventral margin of the neural canal in all preserved precloacal vertebrae^21^.

The prezygapophyseal buttress is massive, lacks a prezygapophyseal process and bears an oblique, blunt ridge anteriorly (Fig. 2F,K). In lateral view, the buttress is succeeded posteriorly by an elliptical fossa (Fig. 2C,H,R). The fossa occurs immediately ventral to the interzygapophyseal ridge and medial to the diapophysis, similar to *Madtsoia pisdurensis* (Mohabey et al.^8^). The prezygapophyseal facets are elliptical (5022–4, przL/przW = 1.3) and inclined ventromedially (prα = 20°–28°; Fig. 2A,D,F,I). In dorsal view, these facets diverge at 45° from the sagittal plane, contrary to the transversely oriented facets in *Madtsoia bai*^10^, *Madtsoia madagascariensis*^1^, *Platyspondylophis*^21^, and *Yurlunggur*^11^. Strongly divergent prezygapophyses are also observed in *Gigantophis garstini*^2^ (~ 70°) and *Eomadtsoia*^7^ (60°–80°). The postzygapophyseal facets in *Vasuki* are also elliptical (IITR/VPL/SB 3102-8II, pozL/pozW = 1.2; Supplementary Table 2) and medioventrally oriented (poα = 12°–26°; Figs. 2G,J, 3B,E). The interzygapophyseal ridge is thick and posterodorsally directed, acting as a bridge between the pre- and postzygapophyses. A small lateral foramen is present ventral to the ridge (Fig. 3L,Q) as in *Powellophis*^3^. In dorsal view the interzygapophyseal ridges are straight and differ from the arcuate ridges seen in most madtsoiids [e.g., *Madtsoia*, *Gigantophis garstini*, *Wonambi*, *Yurlunggur* and *Platyspondylophis*]^2,8,10,11,13,18,21,28^.

The neural canal is reniform (Figs. 2P,Q, 3F,G) in cross-section and significantly wider than high (ncW/ncH = 3–3.6). It differs from the comparatively narrower and trilobate neural canal in *Gigantophis garstini*^2^ (NHMUK R8344, ncW/ncH = 2.3), *Platyspondylophis* (WIF/A 2271, ncW/ncH = 2.1), *Madtsoia* (ncW/ncH = 1.3–2.3), *Yurlunggur* (NTM P8695-243, ncW/ncH = 2.3), “*Gigantophis* sp.” (CPAG-RANKT-V-1, ncW/ncH = 1.8) and *Powellophis* (PVL 4714–4, ncW/ncH = 1.6), and the sub-elliptical canal in *Wonambi* (QMF23038, ncW/ncH = 1.3).

The zygosphene is trapezoidal and mediolaterally wider than high (zsW/zsH = 1.4–1.8; Fig. 2A,K), as in *Gigantophis garstini* (NHMUK R8344, zsW/zsH = 2^2^), *Madtsoia bai* (AMNH 3155, zsW/zsH = 1.8) and *Madtsoia madagascariensis* (FMNH PR 2551, zsW/zsH = 1.9). Transversely much wider zygosphenes characterize *Nidophis* (LPB FGGUB v.547/1, zsW/zsH = 5), *Madtsoia camposi* (DGM 1310a, zsW/zsH = 2.8), *Eomadtsoia* (MPEF-PV 2378, zsW/zsH = 2.6), *Platyspondylophis* (WIF/A 2269, zsW/zsH = 2.2) and *Patagoniophis* (QMF 19717, zsW/zsH = 5). In *Vasuki*, the zygosphene is wider than the cotyle, contrary to *Gigantophis garstini*, “*Gigantophis* sp.”, *Platyspondylophis* and *Madtsoia*^1,8,10,13,16,21^. In anterior view, dorsal margin of the zygosphene is straight and the articular facets are steeply inclined (~ 40° form the vertical; Figs. 2F,P, 3A). These facets are oval in lateral view (IITR/VPL/SB 3102–6, zsfL/zsfW = 1.1). The anterior zygosphenal margin is markedly notched in dorsal view (zsα = 118°–128°; Figs. 2I,N, 3N), and differs from the non-notched zygosphene in *Madtsoia pisdurensis*^8^, *Madtsoia camposi*^13^, *Eomadtsoia*^7^ and *Platyspondylophis*^21^. In “*Gigantophis* sp.” (zsα = 145°) and *Madtsoia madagascariensis* (zsα = 145°–147°) the zygosphene is weakly notched.

The zygantrum is mediolaterally wider than high, with steeply inclined facets (50°–60° from the horizontal; Fig. 2B,G,Q). The facets are elliptical in posterior view, but devoid of a median wall present in *Gigantophis garstini*^2^. An anteroventrally directed fossa is present at the base of each facet, and accommodates an endozygantral foramen (Figs. 2G, 3B). The latter is also present in *Madtsoia madagascariensis*^1^, *Powellophis*^3^ and *Gigantophis garstini*^2^. In *Vasuki*, the zygantral roof above each facet is medio-dorsally convex and descends as sub-vertical ridges into the zygantrum (Fig. 2Q) as in *Madtsoia madagascariensis*^1^. The roof is ventrally convex in *Eomadtsoia* and *Madtsoia pisdurensis*, and straight in *Powellophis*, *Platyspondylophis*, *Yurlunggur* and *Gigantophis garstini*^3,7,8,11,21^. A large, dorsolaterally oriented, elliptical parazygantral fossa flanks the zygantrum laterally on either side and bears a small parazygantral foramina (Fig. 2B,G,Q).

The neural spine is dorsoventrally high (MTV, nsH/tvH = 0.21–0.29, Supplementary Table 2) and buttressed posteriorly by the neural arch laminae (Fig. 3B–D,V,W). The latter extend anterodorsally from the dorsolateral margin of the postzygapophyses up to the dorsal spinal margin, resulting in a deep median embayment. In lateral view, the spine is steeply inclined posterodorsally (12°–19° from the vertical) with a concave anterior and a straight posterior margin. While a high neural spine characterizes most large madtsoiids [*Madtsoia camposi* (DGM, 1310b, MTV, nsH/tvH = 0.22), *Madtsoia madagascariensis* (FMNH PR 2551, MTV, nsH/tvH = 0.33), *Madtsoia bai* (AMNH 3154, MTV, nsH/tvH = 0.22), *Wonambi* (QMF23038, MTV, nsH/tvH = 0.27)], it is more gently inclined in these large-sized taxa [e.g., *Madtsoia madagascariensis* (27°–33°), *Wonambi* (30°), *Gigantophis garstini* (30°)]. A convex anterior margin in *Madtsoia madagascariensis* as well as *Powellophis* and *Nanowana* further distinguishes them from *Vasuki*. Furthermore, the presence of a sharp postspinal lamina (sensu Tschopp^29^) on the posterior spinal surface and a spade-shaped cross-section of the spine differentiates *Vasuki* from other madtsoiids (Figs. 2D,S, 3D). In dorsal view, the neural spine base is flanked on either side by a prominent fossa (Fig. 2I,S), as in *Madtsoia pisdurensis*^8^ and *Madtsoia madagascariensis*^1^. The fossae occur immediately posterior to the zygosphene and are bordered ventrally by weak, rounded bony struts emanating from the posterolateral zygosphenal margin. Ventral to these struts, a prominent foramen is present on the dorsal surface of the neural arch posterior to the zygosphene (Fig. 2I), similar to *Madtsoia madagascariensis*^1^.

In ventral view, the centrum is triangular and widest across the parapophyses. Large paired subcentral fossae, more prominent in the anterior trunk vertebrae (ATV), occupy most of the ventral surface of the centrum (Figs. 2J,O, 3E,T). The fossae are bordered laterally by robust subcentral ridges that extend posteromedially from the parapophyses to the dorsoventral midpoint of the condyle. These ridges are straight to weakly convex in ventral view and differ from the concave ridges in *Patagoniophis*^28^ and *Madtsoia madagascariensis*^1^. The subcentral fossae are separated by a transversely convex low hemal keel (Figs. 2T, 3E,O,T). The latter is broad, weakly raised and terminates anterior to the precondylar constriction. The hemal keel is not prominent, unlike the narrow/sharp keel in “*Gigantophis* sp.”, *Eomadtsoia*, *Nidophis*, *Nanowana* and *Powellophis*^2,3,7,9,16^. In *Vasuki*, this keel remains dorsal to the ventral parapophyseal margin (Figs. 2M,R, 3M,R,V) unlike the hemal keel of other madtsoiids which descends below the parapophysis. Consequently, we identify the disposition of the hemal keel as an autapomorphy of *Vasuki*.

A small subcentral foramen is present on either side of the ventral shaft in *Vasuki* (Fig. 2E,O,T), as in *Madtsoia madagascariensis*^1^, *Madtsoia camposi*^13^, *Nidophis*^9^, and *Patagoniophis*^28^. The hypapophysis is paddle-like with sharp lateral margins and extends up to the level of the ventral condylar rim in ATV (Fig. 2G,H,J,L,M,O). The hypapophysis is directed posteroventrally unlike the ventrally directed hypapophysis in *Madtsoia madagascariensis*^1^ and *Patagoniophis*^28^. Across the precloacals, the hypapophysis progressively reduces in prominence and is replaced by a chisel shaped structure with paired protuberances separated from the ventral condylar rim by a short, sharp ridge in the mid-trunk vertebrae (MTV; Fig. 3J,O,T,X). This chisel shaped structure appears autapomorphic for *Vasuki* as it differs from the condition in other madtsoiids.

### Phylogenetic analysis

**Figure 4:**
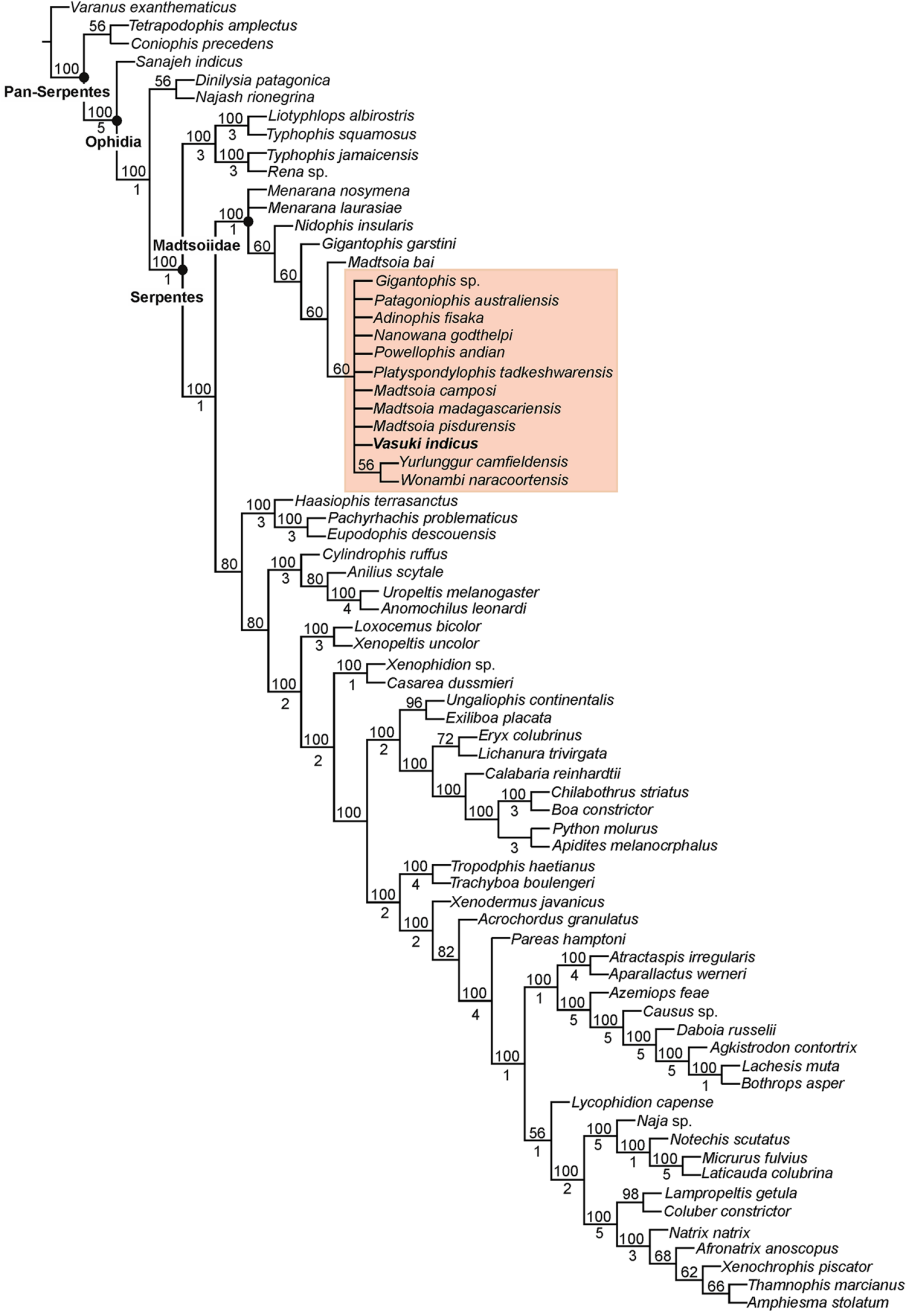
Phylogenetic position of *Vasuki indicus* gen. et sp. nov. IITR/VPL/SB 3102 in 50% majority-rule tree of Analysis 1. Clade comprising *Vasuki indicus* highlighted in pink. Numbers above and below nodes indicate the frequency a clade is represented in the most parsimonious trees and Bremer support values, respectively.

**Figure 5:**
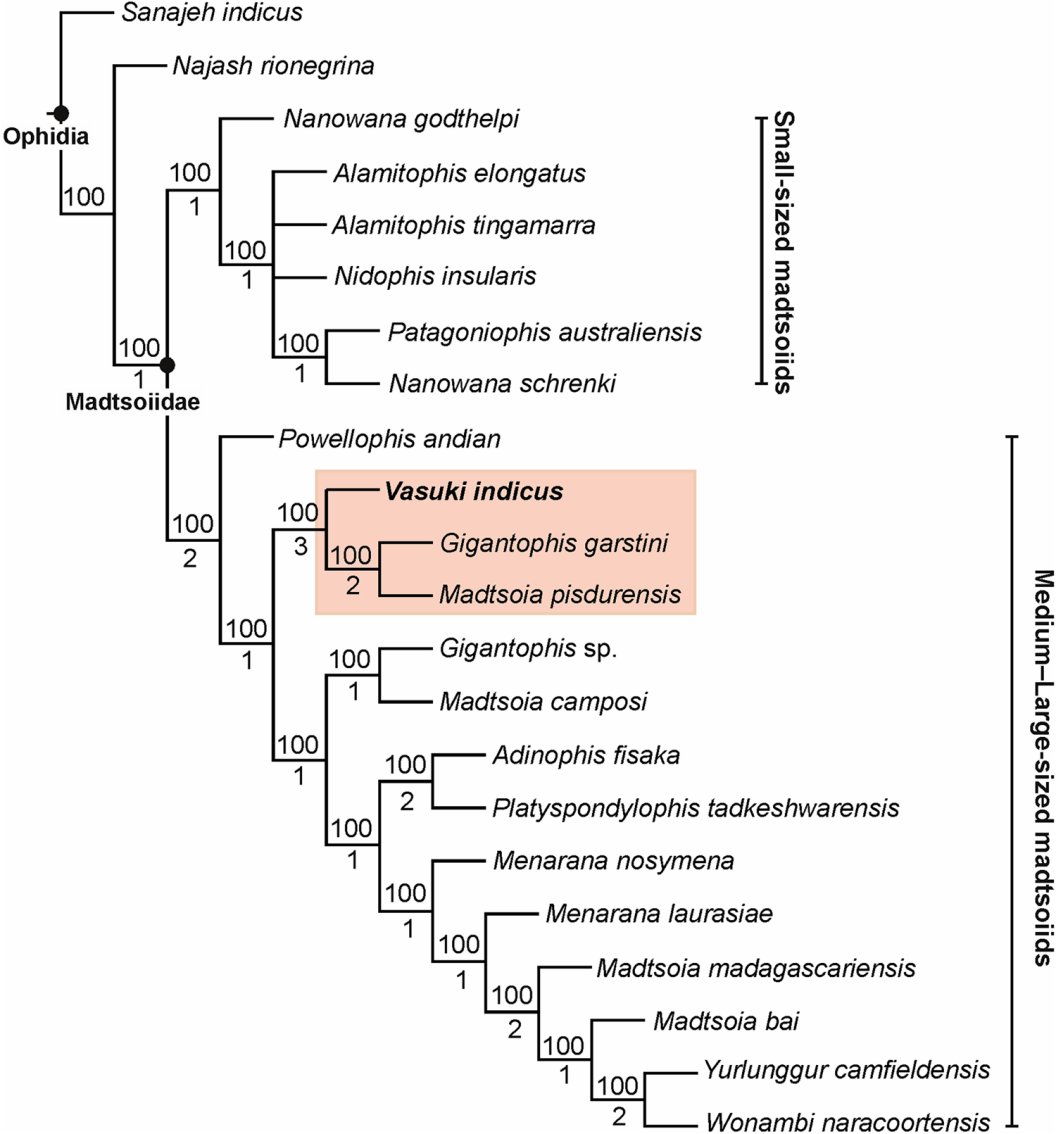
Phylogenetic position of *Vasuki indicus* gen. et sp. nov. IITR/VPL/SB 3102 in 50% majority-rule tree of Analysis 2. Clade comprising *Vasuki indicus* highlighted in pink. Numbers above and below nodes indicate the frequency a clade is represented in the most parsimonious trees and Bremer support values, respectively.

The position of *Vasuki* within Madtsoiidae was tested in a modified version of the character-taxon matrix of Zaher et al.^30^ (Analysis 1; see “Methods” section and Supplementary Note 2). 50 most parsimonious trees were recovered with a tree length of 1610, consistency index (CI) of 0.386 and retention index (RI) of 0.73. The resultant tree topologies are largely consistent with Zaher et al.^30^ as Madtsoiidae was recovered as a distinct clade within crown Serpentes (Fig. 4, Supplementary Fig. 3). Madtsoiidae, however, was poorly resolved and did not provide insights into the inter-relationship of *Vasuki* with the other members of the clade. The poor resolution is likely a reflection of the absence of cranial material in majority of madtsoiids and a function of the large matrix where very few vertebral characters could be scored for most madtsoiid taxa. We, therefore, ran a second analysis (Analysis 2) by removing all non-madtsoiid Serpentes and combining the cranial and vertebral characters of Zaher et al.^30^ and Garberoglio et al.^3^, respectively (see “Methods” section and Supplementary Note 3). The latter dataset was used because as the study focused on madtsoiid ingroup relationships. Our analysis recovered only two most parsimonious trees with a tree length of 191, CI of 0.634 and RI of 0.62. Both trees (Fig. 5, Supplementary Fig. 4) were mostly well resolved and the resultant topologies largely consistent with recent studies^2,3,7^ on madtsoiid inter-relationships. Madtsoiidae shows size-based clustering with the small (< 2 m) and medium–large bodied (> 3 m) taxa recovered as separate clades (Fig. 5). *Vasuki* is nested within a distinct clade (Bremer support = 3) as a sister taxon to Indian Late Cretaceous *Madtsoia pisdurensis* + North African Late Eocene *Gigantophis garstini*.

### Estimation of body length

**Figure 6:**
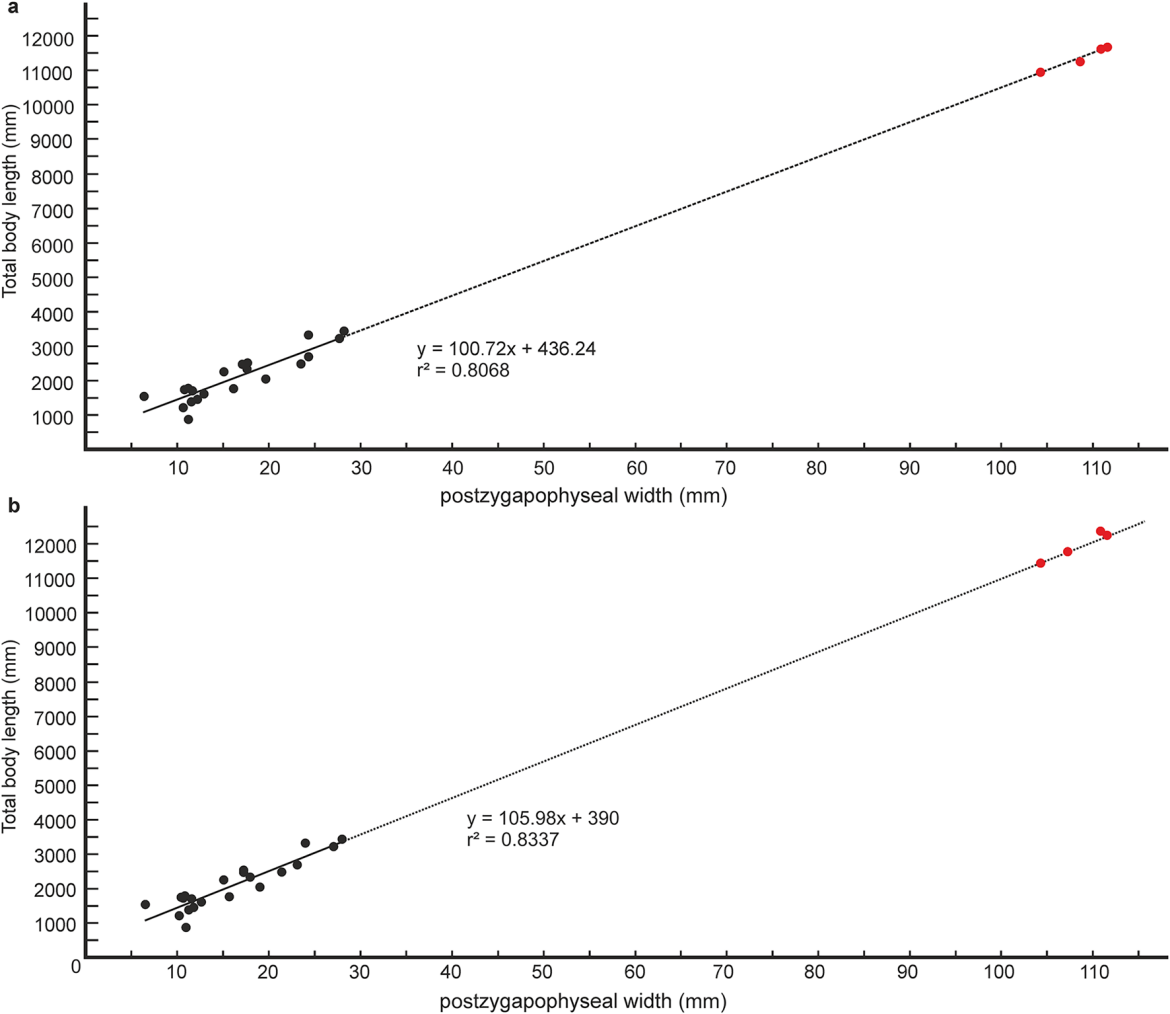
Regressions of vertebral metrics on total body length in extant boine taxa. Regression of postygapophyseal width on total body length in extant boine taxa from vertebrae 60% posteriorly along the vertebral column; p = 0.00000003, standard error =  ± 0.3 m (**a**). Regression of postygapophyseal width on total body length in extant boine taxa from vertebrae 65% posteriorly along the vertebral column; p = 0.00000001, standard error =  ± 0.2 m (**b**). Measurements of extant boine snakes taken from Head et al.^31^ and plotted as black circles. Estimated body lengths of *Vasuki indicus* shown in red.

**Figure 7:**
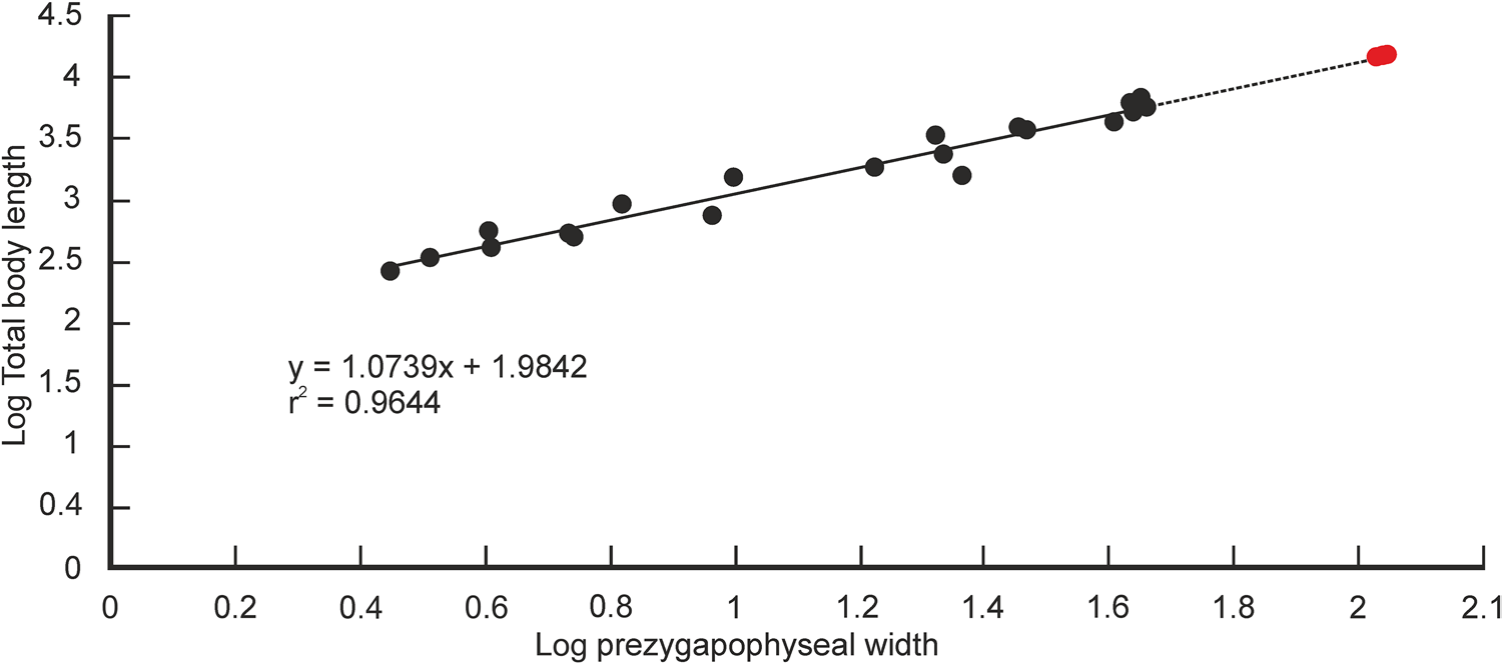
Regression of total body length on prezygapophyseal width in extant snakes. Measurements of extant snakes taken from McCartney et al.^32^ and plotted as black circles. Estimated body lengths of *Vasuki indicus* shown in red. p = 0.000000000000003; standard error =  ± 0.09 m.

Quantitative estimates of total body length (TBL) of *Vasuki* were made based on two separate methods which have been used in recent years for size estimation of extinct large-bodied snakes (see “Methods” section and Supplementary Tables 3–5). In these methods TBL was regressed on the postzygapophyseal width (following Head et al.^31^; Rio and Mannion^2^) and the prezygapophyseal width (= trans-prezygapophyseal width; following McCartney et al.^32^, Garberoglio et al.^3^), respectively. In the present study estimates were made from MTV (IITR/VPL/SB 3102-4, 3102-8I–II, 3102-11II–III), the largest specimens in the collection, following Rio and Mannion^2^, McCartney et al.^32^ and Garberoglio et al.^3^. Both regression models were statistically significant (p < 0.05) and had a high explanatory power (r^2^ = 0.83–0.96) which asserts their validity. The TBL estimates following Head et al.^31^ ranges between 10.9 and 12.2 m (Fig. 6A,B), whereas those following McCartney et al.^32^ is between 14.5 and 15.2 m (Fig. 7A). These estimates, however, should be treated with caution as the collection lacks posterior precloacal and cloacal vertebrae, and an understanding of the intracolumnar variation in madtsoiids is currently non-existent.

It is worth noting that the largest body-length estimates of *Vasuki* appear to exceed that of *Titanoboa*, even though the vertebral dimensions of the Indian taxon are slightly smaller than those of *Titanoboa*. We acknowledge that this observation may be a reflection of the different datasets used to formulate the predictive equations. However, we do not disregard the results based on the dataset of MacCartney et al.^32^, since the equations derived from the dataset of Head et al.^31^ involve measurements of extant boine taxa that are taken from vertebrae 60–65% posteriorly along the column. Caution is warranted here because of the uncertainties surrounding the phylogenetic position of Madtsoiidae relative to crown snakes which make estimations based on a model depicting intracolumnar variation in vertebral morphology of a particular extant family/taxa tentative. Consequently, predictive regression equations following McCartney et al.^32^, which comprise vertebral data from an array of extant snakes, are also considered in our study.

## Discussion

### Phylogenetic implications

The analyses presented here recovered a monophyletic Madtsoiidae with the clade placed within crown Serpentes in Analysis 1 (Fig. 4, Supplementary Fig. 3). This is in accordance with most phylogenetic studies which assessed the relationship of snake total group within Squamata^30,33–35^. Furthermore, similar to Zaher et al.^30^, the tree topology in Analysis 1 recovered *Sanajeh*, *Diniliysia*, *Najash* stemward of crown Serpentes. Although the clade Madtsoiidae remains poorly resolved in Analysis 1, we found a combination of five synapomorphies supporting the placement of *Vasuki* within Madtsoiidae [centrum broad and subtriangular (ch 613); deep V-shaped embayment along posterior margin of neural arch (ch 614); presence of well-developed paracotylar foramina (ch 615); absence of prezygapophyseal accessory process (ch 616); presence of parazygantral foramina (ch 617)].

On the other hand, Analysis 2 gave insights into the ingroup relationships of Madtsoiidae (Fig. 5, Supplementary Fig. 4). The resultant topologies are largely comparable with previous phylogenetic results^2,3,7,9^, as the taxa were found to resolve into two size-based clades (large vs small). While the possibility of size-related features driving such groupings cannot be ruled out, the recovery of small–medium sized taxa (e.g., *Adinophis*, *Menarana*, *Powellophis*) within the large bodied clade suggests the presence of size-independent characters supporting these clades. A similar argument was also put forward by Garberoglio et al.^3^ while discussing the occurrence of size-based clades within Madtsoiidae. However, none of the speciose genera (e.g., *Madtsoia*, *Nanowana*, *Menarana*) included in this study formed monophyletic clades. The Bremer support for most internal nodes within Madtsoiidae remains low, although a few have comparatively higher support (Fig. 5). These results highlight the need for more rigorous sampling involving a better anatomical coverage of madtsoiids, leading to more robust phylogenetic relationships.

A unique combination of 7 synapomorphies nest *Vasuki* within Madtsoiidae [well-developed paracotylar foramina (ch 610); median prominence on ventral margin of centrum (ch 611); coW:dW between 0.5 and 0.3 (ch 634); lateral ridge on precloacal vertebrae below lateral foramen (ch 635); thick zygosphene (ch 645); moderately high neural spine (ch 648); lateral foramina present dorsal to subcentral ridges (ch 650)]. Furthermore, a combination of 6 unambiguous synapomorphies [posterior neural arch margin with deep V-shaped embayment (ch 614); oval precloacal cotyle (ch 615); transversely wide vertebrae (ch 629); hemal keel not sharp and narrow (ch 633); neural spine posteriorly canted (ch 652); presence of posterior process of hemal keel (ch 653)] support the placement of *Vasuki* with *Gigantophis garstini* and *Madtsoia pisdurensis*. Moreover, a single autapomorphy characterises *Vasuki*—chisel-shaped process of hemal keel (ch 654).

It is noteworthy that some of the synapomorphies mentioned above may be individually plesiomorphic characters, it is the unique combination of characters that justifies the recovery of *Vasuki* within Madtsoiidae. Previous studies (e.g., Head et al.^31^; Mohabey et al.^8^) have used character combinations to diagnose Madtsoiidae and other snake taxa.

### Body length estimation and paleoecology

Our TBL estimations show that *Vasuki* was not only the largest madtsoiid (Table 1) but one of the largest snakes ever reported. Its vertebral dimensions are second only to the Paleocene Boinae *Titanoboa* (Head et al.^31^). We attempted to infer the paleoecology of this large Indian madtsoiid from vertebral morphology since several previous studies on other extinct snakes (e.g., *Palaeophis colossaeus*, *Powellophis* and *Madtsoia madagascarensis*) have highlighted the importance of vertebrae in paleoecological reconstructions^1,3,32^. The transversely wide vertebrae of *Vasuki* bear mainly laterally-directed synapophyses which would have been associated with laterally directed ribs, suggesting a broad and cylindrical body (see McCartney et al.^32^). These features suggest a non-aquatic lifestyle for *Vasuki* as opposed to aquatic snakes which may possess high pterapophyses and have laterally compressed vertebrae with ventrally facing synapophyses, thereby placing the ribs beneath the vertebrae^3,32,36^. A high pterapophysis, however, is absent in many aquatic snakes and changes in the orientation of synapophyses from ventral to lateral across the vertebral column have been previously noted in aquatic snakes such as *Simoliophis*^37^. In hydrophiine sea snakes the vertebrae show true lateral compression only in the caudal region. Therefore, the possibility of an aquatic lifestyle for this giant Indian madtsoiid cannot be completely ruled out. An arboreal lifestyle is unlikely, judging from the large size of *Vasuki* and the fact that arboreal snakes tend to have elongated vertebrae with short zygapophyses^38^. A non-fossorial habitat is inferred here for *Vasuki* based on large body-size and non-depressed neural arch-spine complexes which would have placed the dorsal muscles (e.g., M. Semispinalis et spinalis, M. Interarticularis superior) away from the sagittal plane (sensu Auffenburg^39^)^1,3^. This is further supported by the inferred presence of dorsoventrally thick *M. multifidus*, which originates from the anterodorsal neural spinal surface and inserts anteriorly onto the posterior margin of the neural arch laminae of the preceding vertebra. Gross similarity in vertebral morphology with extant large-bodied pythonids (e.g., *Python* and *Malayopython*)^40^ suggests a terrestrial/semi-aquatic paleohabitat for *Vasuki*. Corroborative evidence comes from the depositional environment of the *Vasuki*-yielding horizon, which was reconstructed as a back swamp marsh^23,41–45^, similar to the habitat of modern large pythonids.

**Table 1:** Comparison of body length estimates of *Vasuki indicus* and other large-bodied matdsoiids.

Taxa	Specimen number	Estimated body length
*Vasuki indicus*	IITR/VPL/SB 3102-8II	11.6^a^; 12.1^b^; 15.2^c^
*Madtsoia pisduriensis*	225/GSI/PAL/CR/10	4.7^a^; 4.9^b^; 5.2^c^
*Madtsoia camposi*	DGM1311	3.6^a^; 3.7^b^; 4.1^c^
*Madtsoia bai*	AMHN 3154	3.8^a^; 3.9^b^; 4^c^
“*Gigantophis* sp.”	(CPAG-RANKT-V-1)	6^a^;6.3^b^; 6.8^c^
*Madtsoia madagascarensis*	–	5–8^d^
*Gigantophis garstini*	NHMUK R8344A	~ 7^e^
*Platyspondylophis tadkeshwarensis*	WIF/A 2269	~ 5^f^
*Yurlunggur * sp.	–	4.7–5.7^g^

All estimates are in meters. Superscripts indicate source of estimates where a–c refer to predictive equations used in the present study (^a^y = 100.72x + 436.24, ^b^y = 105.98x + 390, ^c^y = 1.0739x + 1.9842); ^d^LaDuke et al.^1^; ^e^Rio and Mannion^2^; ^f^Smith et al.^21^; ^g^Scanlon^26^. See Figs. 6 and 7 for p-values and standard errors of predictive equations; see Abbreviations section.

*Vasuki* is envisaged as a slow-moving snake that possibly adopted a rectilinear locomotory mechanism as indicated by its large size, anteroposteriorly short and transversely wide vertebrae and absence of accessory prezygapophyseal processes^1,38,46^. A similar, anatomy-based inference was also drawn for the large Malagasy *Madtsoia madagascariensis*^1^, although rectilinear locomotion has also been documented in extant snakes with well-developed prezygapophyseal processes, such as vipers^47^. In spite of the uncertainties associated with the locomotory mechanism of *Vasuki*, it was perhaps too large to be an active forager and was more likely an ambush predator that would subdue its prey through constriction, similar to modern anacondas and large-bodied pythonids^1,42,48^.

The new Indian madtsoiid suggests a relatively warm climate (~ 28 °C) for the Middle Eocene (early Lutetian) paleogeographic position of India within the tropical zone^49,50^. This inference stems mainly from empirically derived dependence of poikilotherm body temperature on the ambient environmental temperature, which in turn controls the maximum body size^31,32,51^. Following Head et al.^31^, the mean annual paleotemperature (MAPT) for the Middle Eocene was estimated based on a relationship between the present mean annual temperature (MAT), TBL difference between *Vasuki* and reticulated python (*Malayopython reticulatus*, the longest known extant snake)^42^ and the mass-specific metabolic rate of pythons (see “Methods” section). The predicted MAPT falls between 27.2 and 28.6 °C, corresponding to the temperature range necessary for the survival of an 11–15 m snake, and suggests the Middle Eocene tropics were 0.7–2.1 °C (ΔT) warmer than at present (MAT = 26.5 °C^52^). These estimates are largely comparable to those for the Palaeocene and Late Cretaceous based on the extinct *Titanoboa* (ΔT = 1.9–3.7 °C) and the frog *Beelzebufo ampinga* (ΔT = 2.1 °C), respectively^52^. Studies based on δ^18^O isotopic ratios from foraminifera and TEX86 index^53–56^ have predicted high tropical sea surface temperatures (≥ 30 °C) during the Middle Eocene at ~ 47 Ma, whereas some estimates suggest tropical cooling for the early Middle and Late Eocene, but particularly during 45–34Ma^57,58^. The paleotemperature inferred here (< 30 °C) are lower than the afore-mentioned estimates (≥ 30 °C), but suggests that the Middle Eocene (early Lutetian, ~ 47 Ma) climate was warmer than at present.

A possible limitation of this study could be the use of a pythonid (*Malayopython reticulatus*) as the modern analog, especially since Pythonoidae and Madtsoiidae are phylogenetically distant. However, our choice of a modern analog is based on the inferred foraging mode and terrestrial/semi-aquatic paleohabitat of *Vasuki*, using anatomical data and the depositional environment of the fossiliferous horizon. The latter are similar to those of modern large pythonids which known to inhabit swamps, marshes and lowland forests^41–43,45^.

In India, Paleogene hyperthermal events, such as PETM and ETM2 are well documented from the Kutch and Cambay basins of western India based on δ^13^C negative excursions^23,59–61^. In comparison, studies on Paleogene paleotemperatures are scarce. Based on oxygen isotopic ratios, temperatures in excess of 30 °C were determined for the late Paleocene and early Eocene, whereas lower temperatures, ranging between 22 and 28 °C, were reported for the Middle–Late Eocene (~ 45–37 Ma)^62,63^. Our new estimates show that while the paleoclimate during the Middle Eocene (~ 47 Ma) became cooler compared to the Late Paleocene and early Eocene, it was still higher than at present. Further studies on Paleogene climates in the context of squamate speciation and extinction pattern are necessary in view of their suggested correlation with temperature patterns^64–66^.

### Paleobiogeography

**Figure 8:**
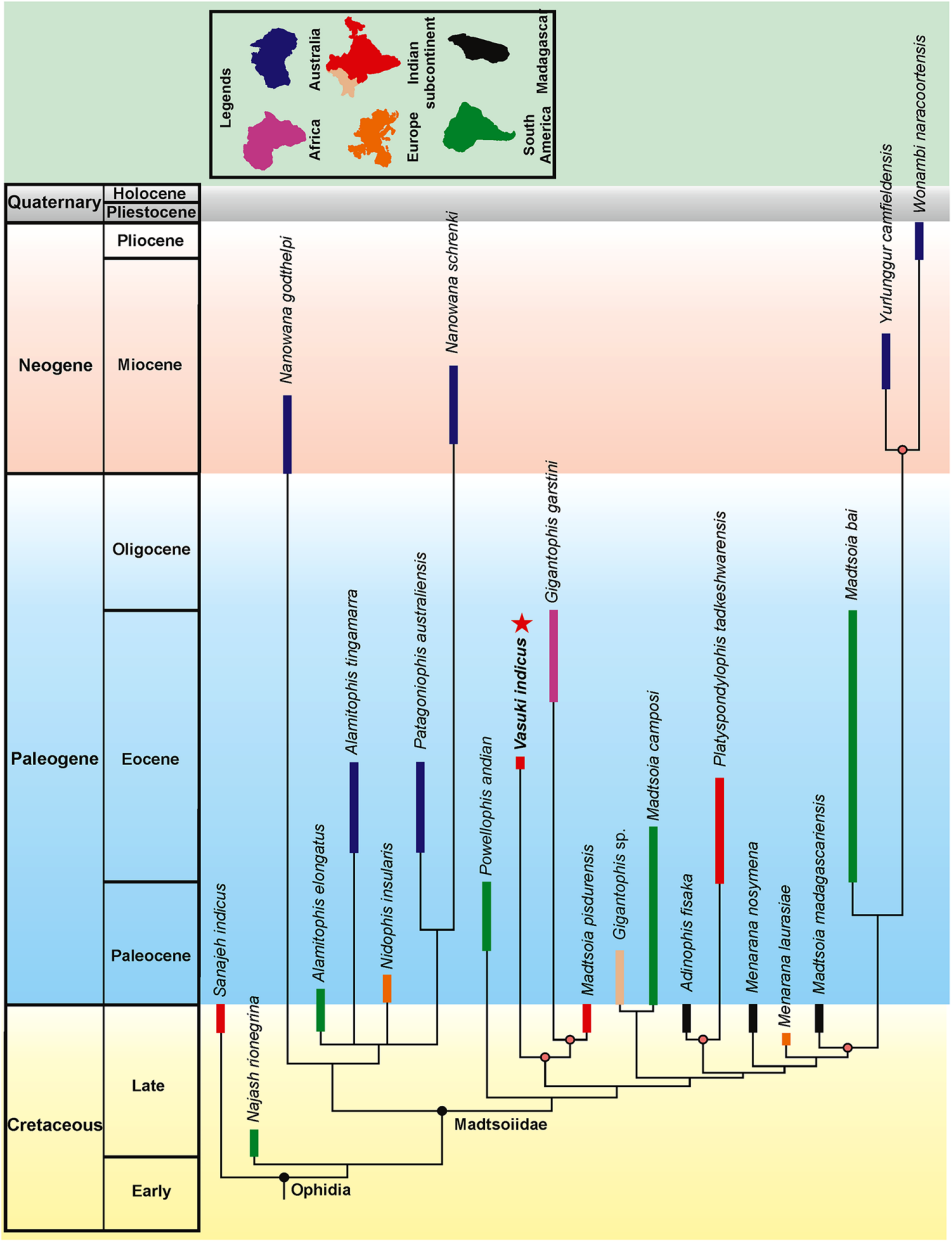
Time-calibrated phylogenetic tree, based on the 50% majority-rule tree of Fig. 5. Red star indicates position of *Vasuki indicus*. Clade for which biogeographic scenarios have been discussed are marked with colored nodes.

**Figure 9:**
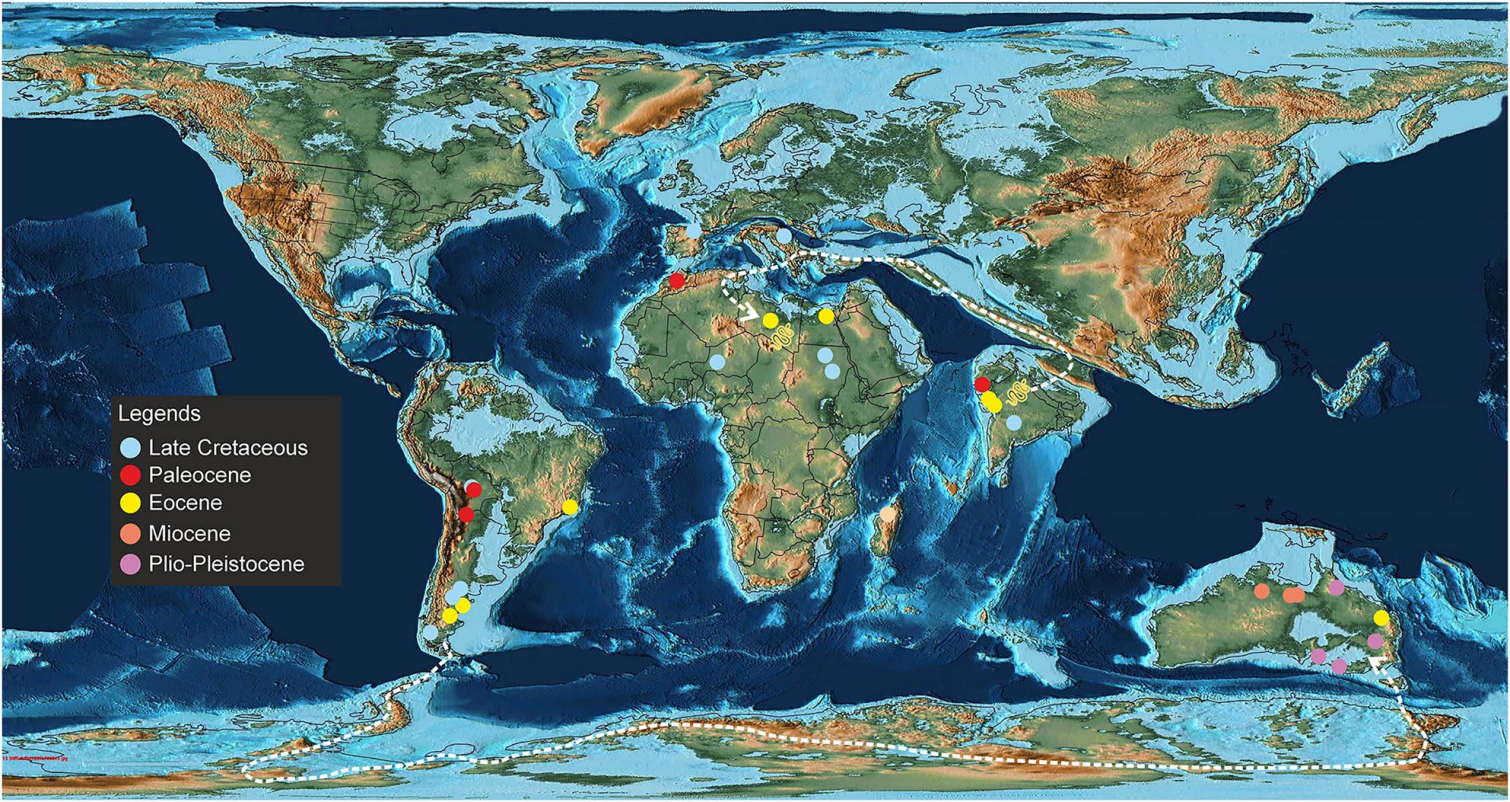
Palaeogeographic distribution of madtsoiids with taxa of different ages plotted together in a simplified Middle Eocene (50 Ma) map to show their global spatio-temporal occurences. Dashed-lines indicate possible dispersal routes between South America and Australia and the Indian subcontinent and North Africa. Palaeogeographic map after Scotese^43^ and sourced from https://www.earthbyte.org/paleomap-paleoatlas-for-gplates/ [This work is licensed under the Creative Commons Attribution 4.0 International License. http://creativecommons.org/licenses/by/4.0/]. Source of information on madtsoiid distribution from the Paleobiology database (https://www.paleo-biodb.org/).

Madtsoiids were a major group of terrestrial snakes whose temporal range straddles the Cretaceous–Paleogene boundary. Fossil occurrences depict a skewed distribution of these snakes as most taxa are known from the Gondwanan landmasses, except Antarctica^1,2^ (Figs. 8, 9). The Laurasian record is extremely poor with madtsoiids known only from the Late Cretaceous (upper Campanian–Maastrichtian) of southern Europe^9^. The distributional pattern also shows the appearance of taxa on landmasses which were separated during the Late Cretaceous and Cenozoic but which share close phylogenetic relations indicating biogeographic links (sensu LaDuke et al.^1^). This conundrum is aptly illustrated by the presence of *Madtsoia* in the Late Cretaceous (Maastrichtian) of Madagascar and India and the Early Paleogene of South America, and *Menarana* in the Maastrichtian of Madagascar and Spain^1,8^ (Fig. 8). Previous studies put forward multiple scenarios for madtsoiid paleobiogeography including—a pan-Gondwanan distribution, albeit unsampled, during the Early Cretaceous followed by regional extinctions and/vicariance; presence of land bridges allowing dispersal between different Gondwanan landmasses and to Europe; sweepstakes dispersal between continents separated by oceanic barriers^1,8,9^. However, Rio and Mannion^2^ argued in favour of an early pan-Gondwanan distribution and trans-Tethyan dispersals between Africa and Europe in the Late Cretaceous. The new Middle Eocene Indian madtsoiid further adds to the complexity of madtsoiid biogeography owing to its close phylogenetic ties with the Late Cretaceous *Madtsoia pisdurensis* from India and the Late Eocene North African *Gigantophis garstini* (Figs. 5, 8).

To assess the biogeographic significance of *Vasuki* we constructed a time-calibrated phylogenetic tree since this approach has been widely used in several previous studies for evaluating the paleobiogeographic significance of different vertebrate groups, including snakes and dinosaurs^2,67–69^. The rationale behind this approach is that phylogenetic relations are widely considered to be suggestive of biogeographic ties^1,70–72^. The phylogenetic tree used here is based on anatomically sparse data because most madtsoiid taxa are known exclusively from vertebrae and lack cranial material, resulting in weak support (Bremer support) for a majority of internal nodes within Madtsoiidae. For this reason, we restricted our biogeographic interpretations only to those nodes which had comparatively higher support (Bremer support ≥ 2; Fig. 8). Overall, the paleobiogeographic scenarios presented here should be treated with caution as future fossil discoveries may alter the phylogenetic position of some madtsoiid taxa and, in turn, the present biogeographic inferences.

Notwithstanding the above-mentioned limitations, the resultant tree in our study is consistent with the current consensus on madtsoiid origins as it suggests a Gondwanan origin reflecting the fact that all known early-diverging taxa are from erstwhile Gondwanan landmasses (Fig. 8). The tree topology argues for biotic exchanges between South America, Madagascar and Australia since the Malagasy *Madtsoia madagascariensis* (Late Cretaceous) and the South American *Madtsoia bai* (Eocene) are successive outgroups to the clade comprising the Neogene *Yurlunggur* and *Wonambi* from Australia. Paleogeographic reconstructions depict fragmentation of most major Gondwana landmasses by the early Cenomanian, with Indo-Madagascar separating from Australia–Antarctica by ~ 110 Ma^73–75^. However, previous studies suggested that land connections between South America and Australia facilitating faunal dispersal through Antarctica persisted till the early Eocene^1,75^ (Fig. 9). On the other hand, the Malagasy–South American–Australian biotic link can likely be explained by the presence of madtsoiids or their most recent common ancestors in these continental blocks prior to their break-up. Recent studies on madtsoiid biogeography envisage an Early Cretaceous pan-Gondwana dispersal of these snakes, with ghost lineages from time-calibrated trees predicting an Aptian origin of Madtsoiidae^1,2,8,9,19^. The fossil record, however, is inconsistent with the hypothesized Early Cretaceous madtsoiid origins since their currently known earliest representatives are from the Coniacian–Santonian of Niger^1,2,9,76,77^. Future sampling from the pre-Maastrichtian horizons of Africa and Indo-Madagascar may help resolve this conundrum.

The Indian madtsoiids, namely *Vasuki indicus*, *Madtsoia pisdurensis*, and *Platyspondylophis tadkeshwarensis*, are resolved into two distinct sub-clades (Fig. 8). *Platyspondylophis* (Ypresian) and the Malagasy *Adinophis fisaka* (Maastrichtian) are recovered as sister-taxa, whereas *Vasuki* (early Lutetian) is the earliest-diverging member of a clade comprising *Madtsoia pisdurensis* (Maastrichtian) and the North African *Gigantophis garstini* (Priabonian). These phylogenetic relations suggest Late Cretaceous–Paleogene biotic exchanges between the Indian subcontinent, Madagascar and North Africa. Among the various competing hypotheses explaining such faunal links, Krause et al.^74^ hypothesized connections (*stepping stones*) between the Indian subcontinent, Madagascar and Africa during the Late Cretaceous, which were possibly destroyed in subsequent tectonic events (e.g., subduction, hotspot related volcanism). The Oman-Kohistan-Ladakh arc (OKL) is another biogeographic pathway which is considered to have facilitated biotic interchanges between North Africa and India following the subcontinent’s collision with OKL at ~ 80 Ma^78^. While there is some support from paleomagnetic and radiometric data for the 80 Ma Indo–OKL collision^78^, subsequent studies based on detrital zircon ages and dating of post-collisional olasses have provided alternate explanations bearing on the sequence of accretion of the OKL with India/Asia^79,80^. These studies support OKL–Eurasia collision by ~ 100–80 Ma, with India colliding with Asia + OKL only during the Paleogene. This makes the possibility of Late Cretaceous Indo–African faunal exchange less likely^2^. More recent studies based on paleomagnetic data propose an initial collision between India and Kohistan-Ladakh arc at ~ 60–50 Ma followed by their final collision with Asia at ~ 45–50 Ma, with the arc being positioned at 8.3 ± 5.6°N at ~ 66–62 Ma^81,82^.

Among the scenarios discussed above we consider the following to be the most plausible explanation for the Indo-Madagascar-North African biotic links suggested by phylogenetic disposition of the Indian madtsoiids:

(i) A sister taxa relationship between the Maastrichtian Malagasy *Adinophis fisaka* and Indian *Platyspondylophis* (Ypresian) suggests a dispersal event at or before Indo-Madagascar separation at ~ 88 Ma^83^. The direction of dispersal, however, remains uncertain as the available fossil evidence does not allow a critical evaluation of this hypothesis due to the poor sampling record of pre-Maastrichtian Malagasy and Indian deposits. However, recovery of *Madtsoia* from the Maastrichtian of both India and Madagascar^8^ (Fig. 8) supports the prevalence of their biotic links, as also suggested by other groups including cordyliform lizards and the nigerophid *Indophis*^75,84^.
(ii) Post Indo-Madagascar separation at ~ 88 Ma, there was extended periods of isolation which ended with collision of the Indian subcontinent + Kohistan-Ladakh arc with Asia in the early Paleogene^50,81,82^ resulting in biogeographic pathways with North Africa through southern Eurasia (Fig. 9).
(iii) *Vasuki*, *Madtsoia pisdurensis* and *Gigantophis garstini* form a distinct clade to the exclusion of others, with the earliest-diverging taxa from India (Fig. 8). This clade also shows close phylogenetic links between Late Cretaceous and Middle Eocene Indian taxa, suggesting a possible Indian origin for this clade. The placement of *Gigantophis garstini* within this clade indicates possible dispersal events from India to North Africa following India-Asia collision, consistent with the Late Eocene (Priabonian, 37–35 Ma^2^) age of *Gigantophis* and recent paleobiogeographic reconstructions showing dispersal routes between India and North Africa via southern Eurasia following the collision^43^ (Fig. 9). Whereas an African origin of *Gigantophis garstini* cannot be ruled out considering the recovery of madtsoiids from the Late Cretaceous deposits of that continent, the taxonomic and phylogenetic uncertainties offer little support for this hypothesis. However, Rio and Mannion’s^2^ alternative explanation that an Early Cretaceous pan-Gondwanan dispersal and long ghost lineages may have led to close phylogenetic relations between *Gigantophis garstini* and the Indian madtsoiids, though potentially valid, is currently weakly supported because of poor sampling.

To summarize, we identify a lineage of exceptionally large-bodied madtsoiids (represented by the largest known madtsoiids, *Vasuki* and *Gigantophis garstini*) which originated in the Indian subcontinent and subsequently spread to Africa via southern Eurasia during the Eocene. The discovery of *Vasuki*, and the sparse anatomical coverage of known madtsoiids highlight the need for rigorous sampling of Late Cretaceous and Paleogene Gondwanan deposits. Recovery of additional material and new taxa (including large-sized forms) may provide further insights into madtsoiid systematics and biogeography.

## Methods

### Osteological description

The osteological description of the skeletal specimens was carried out following the nomenclature of LaDuke et al.^1^, Rio and Mannion^2^ and Mohabey et al.^8^. Different parameters of the fossil specimens were measured (Supplementary Fig. 2) using Mitutoyo digital callipers with a precision of 0.01 mm. Explanatory line drawings are used wherever necessary. The terminology for vertebral laminae and fossae follows Rio and Mannion^2^ and Tschopp^29^.

### Phylogenetic analysis

The phylogenetic affinity of *Vasuki* was assessed in two separate analyses (Analysis 1 and 2). In Analysis 1 (Supplementary Dataset 1) the character-taxon matrix of Zaher et al.^30^ was used. All non-Pan-Serpentes toxicoferans were removed except for *Varanus exanthematicus* which was used as the outgroup. 15 madtsoiid taxa, including *Vasuki*, were added. The character-taxon matrix included 72 taxa and 785 characters. The phylogenetic analysis was performed using TNT version 1.6^85^ where the software memory was set to retain 10,000 trees and a display buffer of 10 Mb. The Traditional Search option was used to analyse the dataset. The constraints for the analysis included 50 replications of Wagner trees, in which the swapping algorithm was bisection reconnection with 10 trees saved per replication. To determine the robustness of the nodes, Bremer support values were calculated using the script bremer.run in which only trees suboptimal by 20 steps were retained.

In Analysis 2 (Supplementary Dataset 2) all non-madtsoiid Serpentes were removed except for the basal ophidians *Najash* and *Sanajeh*. The latter taxon was used as the outgroup. The dataset combined the cranial and vertebral characters of Zaher et al.^30^ and Garberoglio et al.^3^, respectively. 3 additional madtsoiid taxa were included. The character-taxon matrix included 22 taxa and 656 characters. The analysis was performed using TNT version 1.6^85^ following the software settings and search parameters of Analysis 1. The script bremer.run was used to calculate Bremer support values in which only trees suboptimal by 20 steps were retained.

### Time-calibrated tree

This was constructed by plotting the temporal ranges of the snake taxa onto the majority rule tree of Analysis 2 against a numerically calibrated geological time-scale. The temporal ranges of the taxa used in this study have been obtained from the Paleobiology Database (https://www.paleobiodb.org/), Rio and Mannion^2^, and Garberoglio et al.^3^.

### Body length estimation

The body-length estimates of *Vasuki* were based on the datasets of Head et al.^31^ and McCartney et al.^32^. The dataset of Head et al.^31^ comprises measurements of trans-postzygapophyseal width (poW) and TBL of 21 extant boine taxa, whereas that of McCartney et al.^32^ include measurements of trans-prezygapophyseal width and total body length of 21 extant snakes.

The following predictive regression equations were formulated after

1. Head et al.^31^:
  $${\text{y}}\, = \,{1}00.{\text{72x}}\, + \,{436}.{24},$$
  where postzygapophyseal width (x) is equated with the total body length (y). The dataset was from vertebrae 60% posteriorly along the vertebral column, and was not log transformed as the measured parameters were approximately normally distributed (sensu Head et al.^31^).
  $${\text{y}}\, = \,{1}0{5}.{\text{98x}}\, + \,{39}0,$$
  where postzygapophyseal width (x) is equated with the total body length (y). The dataset was from vertebrae 65% posteriorly along the vertebral column, and was not log transformed as the measured parameters were approximately normally distributed (sensu Head et al.^31^).
2. McCartney et al.^32^ and Garberoglio et al.^3^:
  $${\text{y}}\, = \,{1}.0{\text{739x}}\, + \,{1}.{9842},$$
  where trans-prezygapophyseal width (x) is equated with the total body length (y). Log transformed values of the measured parameters were used to normalize the dataset.

In previous studies, body lengths have been estimated for extinct snakes, which are part of extant clades, using maximum likelihood methods^31,32^. Head et al.^31^ developed a model depicting intracolumnar variation of vertebral morphology in extant boines to assign vertebral specimens of the giant extinct boid *Titanoboa* to their most likely position in the vertebral column. Based on vertebral landmarks, the specimens of *Titanoboa* were matched to a position 60–65% posteriorly along the column (MTV, sensu Rio and Mannion^2^), and size estimates were obtained by regressing TBL on poW based on vertebrae of extant boines from those positions. However, such models showing intracolumnar variation in madtsoiids are currently non-existent as very few of these snakes are known from complete/nearly complete vertebral column^2^. Size estimates of *Vasuki* were calculated in this study using MTV following Rio and Mannion^2^, although, these estimates should be considered tentative as the specimens of *Vasuki* cannot be assigned to the same position as the boine vertebrae used to formulate the equations. Also, there may be differences in the relationship between poW and TBL between extant boines and *Vasuki*. Furthermore, uncertainties associated with the phylogenetic position of Madtsoiidae relative to crown snakes, preclude formulation of models showing intracolumnar variation in vertebral morphology based on any extant clade. Consequently, predictive regression equations, based on data from an array of extant snakes from McCartney et al.^32^, were used to determine the body length of the new Indian taxon and therefore, the estimated lengths, though reasonable, should also be treated with caution.

### Estimation of paleotemperature

Paleotemperature estimates were obtained using the following equation provided in Head et al.^31^:

$$\mathrm{MAPT }=MAT+3\mathrm{\alpha }10 \,^\circ {\text{C}}\left(\frac{{{\text{log}}}_{10}\left(TB{L}_{v}/TB{L}_{M}\right)}{{{\text{log}}}_{10}{{\text{Q}}}_{10}}\right),$$

$$\mathrm{MAPT }=MAT+5.1\, ^\circ {\text{C}}\left(\frac{{{\text{log}}}_{10}\left(TB{L}_{v}/TB{L}_{M}\right)}{0.41}\right),$$

where MAPT is the mean annual paleotemperature; MAT is the present mean annual temperature (26.5 °C^52^); TBL_M_ = 10.05 m is the maximum total body length of *Malayopython reticulatus*^41^; TBL_V_ is the maximum estimated body length of *Vasuki* (15.2 m); Q_10_ (mass specific metabolic rate of pythonids) = 2.6^86^; α (metabolic scaling component) = 0.17^52,87^.

Since Madtsoiidae are an extinct clade, the body length of *Malayopython reticulatus* (Serpentes, Pythonidae) was used in the study as it is the longest known extant snake^42^. The choice of *Malayopython* as the modern analog is based on the similarity in gross vertebral morphology and, inferred mode of life and habitat between *Vasuki* and extant large-bodied pythonids^40–43,45^. However, in the absence of extant representatives of madtsoiids or their close relatives, the estimated paleotemperature values should be treated with caution.

## Supplementary Information

Supplementary Information 1.

Supplementary Information 2.

Supplementary Information 3.

Supplementary Information 4.

Supplementary Information 5.

## Data Availability

All data associated with the manuscript are provided in the Supplementary File.

## Abbreviations

AMNH: American Museum of Natural History, New York
CPAG: Centre of Pure and Applied Geology, University of Sindh, Pakistan
DGM: Departamento Nacional de Produção Mineral, Rio de Janeiro, Brazil
FMNH: The Field Museum, Chicago, USA
IITR/VPL/SB: Vertebrate Paleontology Laboratory, Indian Institute of Technology Roorkee, Roorkee, India
LPB FGGUB: Laboratory of Paleontology, Faculty of Geology and Geophysics, University of Bucharest, Bucharest, Romania
MPEF-PV: Vertebrate Paleontological collection, MuseoPaleontológico Egidio Feruglio, Trelew, Chubut Province, Argentina
NTM: Northern Territory Museum, Australia
NHMUK: The Natural History Museum, London, U.K.
QMF: Queensland Museum, Brisbane, Australia
PVL: Vertebrate Paleontological Collection of the Instituto Miguel Lillo, San Miguel de Tucumán, Argentina
WIF/A: Wadia Institute of Himalayan Geology, Dehradun, India

## Code availability

Nomenclatural acts. This published work and the nomenclatural acts it contains have been registered in Zoo- Bank, the proposed online registration system for the International Code of Zoological Nomenclature (ICZN). The LSIDs for this publication are urn:lsid:zoobank.org:act: 2F44E9BE-AE99-45E8-A132-D36A935D3B36 (*Vasuki*) and urn:lsid:zoobank.org:act: 0DD3FB9F-A500-4FFE-842C-EFE51EC76E4D (*V. indicus*).
