# Supplementary material for: Largest known madtsoiid snake from warm Eocene period of India suggests intercontinental Gondwana dispersal
Source: Sci Rep. 2024 Apr 18;14:8054. doi: 10.1038/s41598-024-58377-0 (PMC11549349; doi:10.1038/s41598-024-58377-0)
Supplement: Supplementary file 3 — Supplementary Information 3. [file 41598_2024_58377_MOESM3_ESM.pdf]

Supplementary Dataset for

Largest known madtsoiid snake from warm Eocene period of India suggests intercontinental Gondwana dispersal

Debajit Datta[1,*] & Sunil Bajpai[1,*]

[1]Department of Earth Sciences, Indian Institute of Technology, Roorkee, Uttarakhand 247667, India; debajitdatta.pd@es.iitr.ac.in; debajitdatta9@gmail.com; sunil.bajpai@es.iitr.ac.in

ORCID

Sunil Bajpai: https://orcid.org/0000-0002-2279-445X

Debajit Datta: https://orcid.org/0000-0001-6078-9830

* Authors for correspondence

Supplementary Dataset

1. Supplementary Dataset 2: Character matrix for *Vasuki indicus* gen. et sp. nov. used in the phylogenetic analysis 2.

Supplementary Dataset 2: Character matrix for *Vasuki indicus* gen. et sp. nov.

Taxa/ Characters	1	2	3	4	5	6	7	8	9	10	11	12	13	14	15	16
Sanajeh indicus	1	?	0	?	0	0	?	?	?	?	1	?	?	?	?	?
Najash rionegrina	?	1	?	?	?	?	?	?	?	?	?	?	0	?	?	?
Nanowana godhelpi	?	?	?	?	?	?	?	?	?	?	?	?	?	?	?	?
Alamitophis tingamarra	?	?	?	?	?	?	?	?	?	?	?	?	?	?	?	?
Alamitophis elongatus	?	?	?	?	?	?	?	?	?	?	?	?	?	?	?	?
Nidophis	?	?	?	?	?	?	?	?	?	?	?	?	?	?	?	?
Patagoniophis	?	?	?	?	?	?	?	?	?	?	?	?	?	?	?	?
Nanowana schrenki	?	?	?	?	?	?	?	?	?	?	?	?	?	?	?	?
Powellophis	?	?	?	?	?	?	?	?	?	?	?	?	?	?	?	?
Vasuki indicus	?	?	?	?	?	?	?	?	?	?	?	?	?	?	?	?
Gigantophis garstini	?	?	?	?	?	?	?	?	?	?	?	?	?	?	?	?
Madtsoia pisdurensis	?	?	?	?	?	?	?	?	?	?	?	?	?	?	?	?
Gigantophis sp	?	?	?	?	?	?	?	?	?	?	?	?	?	?	?	?
Madtsoia camposi	?	?	?	?	?	?	?	?	?	?	?	?	?	?	?	?
Adinophis	?	?	?	?	?	?	?	?	?	?	?	?	?	?	?	?
Platyspondylophis	?	?	?	?	?	?	?	?	?	?	?	?	?	?	?	?
Menarana nosymena	?	?	?	?	?	?	?	?	?	?	?	?	?	?	?	?
Menarana laurasiae	?	?	?	?	?	?	?	?	?	?	?	?	?	?	?	?
Madtsoia madagascariensis	?	?	?	?	?	?	?	?	?	?	?	?	?	?	?	?
Madtsoia bai	?	?	?	?	?	?	?	?	?	?	?	?	?	?	?	?
Yurlunggur camfieldensis	1	?	0	0	0	0	?	?	0	1	1	0	?	?	?	?
Wonambi naracoortensis	?	?	?	?	?	0	?	?	?	?	1	?	?	?	?	?

Taxa/ Characters	17	18	19	20	21	22	23	24	25	26	27	28	29	30	31	32
Sanajeh indicus	?	?	0	1	?	?	?	?	?	?	?	?	?	?	?	?
Najash rionegrina	0	?	?	?	0	2	?	1	0	?	?	?	?	0	0	?
Nanowana godhelpi	?	?	?	?	?	?	?	?	?	?	?	?	?	?	?	?
Alamitophis tingamarra	?	?	?	?	?	?	?	?	?	?	?	?	?	?	?	?
Alamitophis elongatus	?	?	?	?	?	?	?	?	?	?	?	?	?	?	?	?
Nidophis	?	?	?	?	?	?	?	?	?	?	?	?	?	?	?	?
Patagoniophis	?	?	?	?	?	?	?	?	?	?	?	?	?	?	?	?
Nanowana schrenki	?	?	?	?	?	?	?	?	?	?	?	?	?	?	?	?
Powellophis	?	?	?	?	?	?	?	?	?	?	?	?	?	?	?	?
Vasuki indicus	?	?	?	?	?	?	?	?	?	?	?	?	?	?	?	?
Gigantophis garstini	?	?	?	?	?	?	?	?	?	?	?	?	?	?	?	?
Madtsoia pisdurensis	?	?	?	?	?	?	?	?	?	?	?	?	?	?	?	?
Gigantophis sp	?	?	?	?	?	?	?	?	?	?	?	?	?	?	?	?
Madtsoia camposi	?	?	?	?	?	?	?	?	?	?	?	?	?	?	?	?
Adinophis	?	?	?	?	?	?	?	?	?	?	?	?	?	?	?	?
Platyspondylophis	?	?	?	?	?	?	?	?	?	?	?	?	?	?	?	?
Menarana nosymena	?	?	?	?	?	?	?	?	?	?	?	?	?	?	?	?
Menarana laurasiae	?	?	?	?	?	?	?	?	?	?	?	?	?	?	?	?
Madtsoia madagascariensis	?	?	?	?	?	?	?	?	?	?	?	?	?	?	?	?
Madtsoia bai	?	?	?	?	?	?	?	?	?	?	?	?	?	?	?	?
Yurlunggur camfieldensis	?	?	0	1	0	2	1	1	1	?	?	?	?	0	0	1
Wonambi naracoortensis	?	?	?	?	?	?	1	?	?	?	?	?	?	?	?	?

Taxa/ Characters	33	34	35	36	37	38	39	40	41	42	43	44	45	46	47	48
Sanajeh indicus	?	?	?	?	?	?	?	?	?	?	?	?	?	?	?	?
Najash rionegrina	?	0	0	1	0	?	?	0	0	0	0	0	2	2	?	0
Nanowana godhelpi	?	?	?	?	?	?	?	?	?	?	?	?	?	?	?	?
Alamitophis tingamarra	?	?	?	?	?	?	?	?	?	?	?	?	?	?	?	?
Alamitophis elongatus	?	?	?	?	?	?	?	?	?	?	?	?	?	?	?	?
Nidophis	?	?	?	?	?	?	?	?	?	?	?	?	?	?	?	?
Patagoniophis	?	?	?	?	?	?	?	?	?	?	?	?	?	?	?	?
Nanowana schrenki	?	?	?	?	?	?	?	?	?	?	?	?	?	?	?	?
Powellophis	?	?	?	?	?	?	?	?	?	?	?	?	?	?	?	?
Vasuki indicus	?	?	?	?	?	?	?	?	?	?	?	?	?	?	?	?
Gigantophis garstini	?	?	?	?	?	?	?	?	?	?	?	?	?	?	?	?
Madtsoia pisdurensis	?	?	?	?	?	?	?	?	?	?	?	?	?	?	?	?
Gigantophis sp	?	?	?	?	?	?	?	?	?	?	?	?	?	?	?	?
Madtsoia camposi	?	?	?	?	?	?	?	?	?	?	?	?	?	?	?	?
Adinophis	?	?	?	?	?	?	?	?	?	?	?	?	?	?	?	?
Platyspondylophis	?	?	?	?	?	?	?	?	?	?	?	?	?	?	?	?
Menarana nosymena	?	?	?	?	?	?	?	?	?	?	?	?	?	?	?	?
Menarana laurasiae	?	?	?	?	?	?	?	?	?	?	?	?	?	?	?	?
Madtsoia madagascariensis	?	?	?	?	?	?	?	?	?	?	?	?	?	?	?	?
Madtsoia bai	?	?	?	?	?	?	?	?	?	?	?	?	?	?	?	?
Yurlunggur camfieldensis	0	0	0	1	0	0	0	0	0	0	0	?	2	?	1	1
Wonambi naracoortensis	?	?	0	1	?	?	?	0	0	?	0	?	2	?	1	1

Supplementary Dataset 2: continued

Taxa/ Characters	49	50	51	52	53	54	55	56	57	58	59	60	61	62	63	64
Sanajeh indicus	?	?	?	?	?	?	?	?	?	?	?	?	?	?	?	0
Najash rionegrina	0	?	0	?	0	?	?	2	?	?	0	1	1	0	?	0
Nanowana godhelpi	?	?	?	?	?	?	?	?	?	?	?	?	?	?	?	?
Alamitophis tingamarra	?	?	?	?	?	?	?	?	?	?	?	?	?	?	?	?
Alamitophis elongatus	?	?	?	?	?	?	?	?	?	?	?	?	?	?	?	?
Nidophis	?	?	?	?	?	?	?	?	?	?	?	?	?	?	?	?
Patagoniophis	?	?	?	?	?	?	?	?	?	?	?	?	?	?	?	?
Nanowana schrenki	?	?	?	?	?	?	?	?	?	?	?	?	?	?	?	?
Powellophis	?	?	?	?	?	?	?	?	?	?	?	?	?	?	?	?
Vasuki indicus	?	?	?	?	?	?	?	?	?	?	?	?	?	?	?	?
Gigantophis garstini	?	?	?	?	?	?	?	?	?	?	?	?	?	?	?	?
Madtsoia pisdurensis	?	?	?	?	?	?	?	?	?	?	?	?	?	?	?	?
Gigantophis sp	?	?	?	?	?	?	?	?	?	?	?	?	?	?	?	?
Madtsoia camposi	?	?	?	?	?	?	?	?	?	?	?	?	?	?	?	?
Adinophis	?	?	?	?	?	?	?	?	?	?	?	?	?	?	?	?
Platyspondylophis	?	?	?	?	?	?	?	?	?	?	?	?	?	?	?	?
Menarana nosymena	?	?	?	?	?	?	?	?	?	?	?	?	?	?	?	?
Menarana laurasiae	?	?	?	?	?	?	?	?	?	?	?	?	?	?	?	?
Madtsoia madagascariensis	?	?	?	?	?	?	?	?	?	?	?	?	?	?	?	?
Madtsoia bai	?	?	?	?	?	?	?	?	?	?	?	?	?	?	?	?
Yurlunggur camfieldensis	0	?	?	?	0	0	?	1	?	?	0	?	0	1	?	0
Wonambi naracoortensis	?	0	0	0	0	0	?	?	?	?	0	1	?	?	?	0

Taxa/ Characters	65	66	67	68	69	70	71	72	73	74	75	76	77	78	79	80
Sanajeh indicus	1	1	?	0	1	?	?	?	0	?	?	?	?	?	?	0
Najash rionegrina	2	?	?	?	1	?	?	0	0	0	?	1	2	0	0	1
Nanowana godhelpi	?	?	?	?	?	?	?	?	?	?	?	?	?	?	?	?
Alamitophis tingamarra	?	?	?	?	?	?	?	?	?	?	?	?	?	?	?	?
Alamitophis elongatus	?	?	?	?	?	?	?	?	?	?	?	?	?	?	?	?
Nidophis	?	?	?	?	?	?	?	?	?	?	?	?	?	?	?	?
Patagoniophis	?	?	?	?	?	?	?	?	?	?	?	?	?	?	?	?
Nanowana schrenki	?	?	?	?	?	?	?	?	?	?	?	?	?	?	?	?
Powellophis	?	?	?	?	?	?	?	?	?	?	?	?	?	?	?	?
Vasuki indicus	?	?	?	?	?	?	?	?	?	?	?	?	?	?	?	?
Gigantophis garstini	?	?	?	?	?	?	?	?	?	?	?	?	?	?	?	?
Madtsoia pisdurensis	?	?	?	?	?	?	?	?	?	?	?	?	?	?	?	?
Gigantophis sp	?	?	?	?	?	?	?	?	?	?	?	?	?	?	?	?
Madtsoia camposi	?	?	?	?	?	?	?	?	?	?	?	?	?	?	?	?
Adinophis	?	?	?	?	?	?	?	?	?	?	?	?	?	?	?	?
Platyspondylophis	?	?	?	?	?	?	?	?	?	?	?	?	?	?	?	?
Menarana nosymena	?	?	?	?	?	?	?	?	?	?	?	?	?	?	?	?
Menarana laurasiae	?	?	?	?	?	?	?	?	?	?	?	?	?	?	?	?
Madtsoia madagascariensis	?	?	?	?	?	?	?	?	?	?	?	?	?	?	?	?
Madtsoia bai	?	?	?	?	?	?	?	?	?	?	?	?	?	?	?	?
Yurlunggur camfieldensis	1	3	?	0	0	1	1	0	0	0	?	1	2	0	0	1
Wonambi naracoortensis	1	2	?	?	1	1	1	0	0	0	?	1	?	?	?	1

Taxa/ Characters	81	82	83	84	85	86	87	88	89	90	91	92	93	94	95	96
Sanajeh indicus	0	0	?	?	?	0	0	1	0	2	0	2	?	0	0	1
Najash rionegrina	?	?	?	?	?	?	?	?	?	?	?	?	?	?	?	1
Nanowana godhelpi	?	?	?	?	?	?	?	?	?	?	?	?	?	?	?	?
Alamitophis tingamarra	?	?	?	?	?	?	?	?	?	?	?	?	?	?	?	?
Alamitophis elongatus	?	?	?	?	?	?	?	?	?	?	?	?	?	?	?	?
Nidophis	?	?	?	?	?	?	?	?	?	?	?	?	?	?	?	?
Patagoniophis	?	?	?	?	?	?	?	?	?	?	?	?	?	?	?	?
Nanowana schrenki	?	?	?	?	?	?	?	?	?	?	?	?	?	?	?	?
Powellophis	?	?	?	?	?	?	?	?	?	?	?	?	?	?	?	?
Vasuki indicus	?	?	?	?	?	?	?	?	?	?	?	?	?	?	?	?
Gigantophis garstini	?	?	?	?	?	?	?	?	?	?	?	?	?	?	?	?
Madtsoia pisdurensis	?	?	?	?	?	?	?	?	?	?	?	?	?	?	?	?
Gigantophis sp	?	?	?	?	?	?	?	?	?	?	?	?	?	?	?	?
Madtsoia camposi	?	?	?	?	?	?	?	?	?	?	?	?	?	?	?	?
Adinophis	?	?	?	?	?	?	?	?	?	?	?	?	?	?	?	?
Platyspondylophis	?	?	?	?	?	?	?	?	?	?	?	?	?	?	?	?
Menarana nosymena	?	?	?	?	?	?	?	?	?	?	?	?	?	?	?	?
Menarana laurasiae	?	?	?	?	?	?	?	?	?	?	?	?	?	?	?	?
Madtsoia madagascariensis	?	?	?	?	?	?	?	?	?	?	?	?	?	?	?	?
Madtsoia bai	?	?	?	?	?	?	?	?	?	?	?	?	?	?	?	?
Yurlunggur camfieldensis	?	?	?	?	?	?	?	?	?	?	?	?	?	?	?	1
Wonambi naracoortensis	?	?	?	?	?	?	?	?	?	?	?	?	?	?	?	1

Supplementary Dataset 2: continued

Taxa/ Characters	113	114	115	116	117	118	119	120	121	122	123	124	125	126	127	128
Sanajeh indicus	?	0	?	?	2	0	0	?	?	?	?	?	1	1	?	1
Najash rionegrina	?	0	?	?	2	0	?	?	?	?	?	?	1	?	?	1
Nanowana godhelpi	?	?	?	?	?	?	?	?	?	?	?	?	?	?	?	?
Alamitophis tingamarra	?	?	?	?	?	?	?	?	?	?	?	?	?	?	?	?
Alamitophis elongatus	?	?	?	?	?	?	?	?	?	?	?	?	?	?	?	?
Nidophis	?	?	?	?	?	?	?	?	?	?	?	?	?	?	?	?
Patagoniophis	?	?	?	?	?	?	?	?	?	?	?	?	?	?	?	?
Nanowana schrenki	?	?	?	?	?	?	?	?	?	?	?	?	?	?	?	?
Powellophis	?	?	?	?	?	?	?	?	?	?	?	?	?	?	?	?
Vasuki indicus	?	?	?	?	?	?	?	?	?	?	?	?	?	?	?	?
Gigantophis garstini	?	?	?	?	?	?	?	?	?	?	?	?	?	?	?	?
Madtsoia pisdurensis	?	?	?	?	?	?	?	?	?	?	?	?	?	?	?	?
Gigantophis sp	?	?	?	?	?	?	?	?	?	?	?	?	?	?	?	?
Madtsoia camposi	?	?	?	?	?	?	?	?	?	?	?	?	?	?	?	?
Adinophis	?	?	?	?	?	?	?	?	?	?	?	?	?	?	?	?
Platyspondylophis	?	?	?	?	?	?	?	?	?	?	?	?	?	?	?	?
Menarana nosymena	?	?	?	?	?	?	?	?	?	?	?	?	?	?	?	?
Menarana laurasiae	?	?	?	?	?	?	?	?	?	?	?	?	?	?	?	?
Madtsoia madagascariensis	?	?	?	?	?	?	?	?	?	?	?	?	?	?	?	?
Madtsoia bai	?	?	?	?	?	?	?	?	?	?	?	?	?	?	?	?
Yurlunggur camfieldensis	?	2	?	?	2	0	1	?	?	0	0	?	1	1	?	1
Wonambi naracoortensis	?	0	?	?	?	0	1	?	0	0	0	?	1	?	?	1

Taxa/ Characters	129	130	131	132	133	134	135	136	137	138	139	140	141	142	143	144
Sanajeh indicus	0	?	1	?	0	0	?	0	0	?	0	0	3	?	?	?
Najash rionegrina	?	?	1	0	0	0	?	0	?	?	?	0	3	0	0	0
Nanowana godhelpi	?	?	?	?	?	?	?	?	?	?	0	?	?	?	?	?
Alamitophis tingamarra	?	?	?	?	?	?	?	?	?	?	?	?	?	?	?	?
Alamitophis elongatus	?	?	?	?	?	?	?	?	?	?	?	?	?	?	?	?
Nidophis	?	?	?	?	?	?	?	?	?	?	?	?	?	?	?	?
Patagoniophis	?	?	?	?	?	?	?	?	?	?	?	?	?	?	?	?
Nanowana schrenki	?	?	1	?	?	?	?	?	?	?	?	?	?	?	?	?
Powellophis	?	?	?	?	?	?	?	?	?	?	?	?	?	?	?	?
Vasuki indicus	?	?	?	?	?	?	?	?	?	?	?	?	?	?	?	?
Gigantophis garstini	?	?	?	?	?	?	?	?	?	?	?	?	?	?	?	?
Madtsoia pisdurensis	?	?	?	?	?	?	?	?	?	?	?	?	?	?	?	?
Gigantophis sp	?	?	?	?	?	?	?	?	?	?	?	?	?	?	?	?
Madtsoia camposi	?	?	?	?	?	?	?	?	?	?	?	?	?	?	?	?
Adinophis	?	?	?	?	?	?	?	?	?	?	?	?	?	?	?	?
Platyspondylophis	?	?	?	?	?	?	?	?	?	?	?	?	?	?	?	?
Menarana nosymena	?	?	?	?	?	?	?	?	?	?	?	?	?	?	?	?
Menarana laurasiae	?	?	?	?	?	?	?	?	?	?	?	?	?	?	?	?
Madtsoia madagascariensis	?	?	?	?	?	?	?	?	?	?	?	?	?	?	?	?
Madtsoia bai	?	?	?	?	?	?	?	?	?	?	?	?	?	?	?	?
Yurlunggur camfieldensis	1	1	1	2	0	0	?	0	1	?	0	0	3	0	?	?
Wonambi naracoortensis	1	1	1	2	0	0	?	0	1	0	0	0	3	0	0	0

Taxa/ Characters	145	146	147	148	149	150	151	152	153	154	155	156	157	158	159	160
Sanajeh indicus	0	?	0	0	?	?	?	?	?	?	?	?	?	?	?	?
Najash rionegrina	0	?	0	?	?	?	0	0	?	1	0	0	1	?	?	2
Nanowana godhelpi	?	?	?	?	?	?	?	?	?	?	?	?	?	?	?	?
Alamitophis tingamarra	?	?	?	?	?	?	?	?	?	?	?	?	?	?	?	?
Alamitophis elongatus	?	?	?	?	?	?	?	?	?	?	?	?	?	?	?	?
Nidophis	?	?	?	?	?	?	?	?	?	?	?	?	?	?	?	?
Patagoniophis	?	?	?	?	?	?	?	?	?	?	?	?	?	?	?	?
Nanowana schrenki	?	?	?	?	?	?	?	?	?	?	?	?	?	?	?	?
Powellophis	?	?	?	?	?	?	?	?	?	?	?	?	?	?	?	?
Vasuki indicus	?	?	?	?	?	?	?	?	?	?	?	?	?	?	?	?
Gigantophis garstini	?	?	?	?	?	?	?	?	?	?	?	?	?	?	?	?
Madtsoia pisdurensis	?	?	?	?	?	?	?	?	?	?	?	?	?	?	?	?
Gigantophis sp	?	?	?	?	?	?	?	?	?	?	?	?	?	?	?	?
Madtsoia camposi	?	?	?	?	?	?	?	?	?	?	?	?	?	?	?	?
Adinophis	?	?	?	?	?	?	?	?	?	?	?	?	?	?	?	?
Platyspondylophis	?	?	?	?	?	?	?	?	?	?	?	?	?	?	?	?
Menarana nosymena	?	?	?	?	?	?	?	?	?	?	?	?	?	?	?	?
Menarana laurasiae	?	?	?	?	?	?	?	?	?	?	?	?	?	?	?	?
Madtsoia madagascariensis	?	?	?	?	?	?	?	?	?	?	?	?	?	?	?	?
Madtsoia bai	?	?	?	?	?	?	?	?	?	?	?	?	?	?	?	?
Yurlunggur camfieldensis	0	?	0	0	0	?	?	?	?	1	0	0	1	0	0	2
Wonambi naracoortensis	0	?	0	0	?	?	?	?	?	1	?	?	?	?	?	?

Supplementary Dataset 2: continued

Taxa/ Characters	161	162	163	164	165	166	167	168	169	170	171	172	173	174	175	176
Sanajeh indicus	0	?	?	?	1	?	?	?	?	?	?	?	?	?	?	?
Najash rionegrina	?	?	0	0	1	?	0	0	1	0	?	?	0	2	?	1
Nanowana godhelpi	?	?	?	?	?	?	?	?	?	?	?	?	?	?	?	?
Alamitophis tingamarra	?	?	?	?	?	?	?	?	?	?	?	?	?	?	?	?
Alamitophis elongatus	?	?	?	?	?	?	?	?	?	?	?	?	?	?	?	?
Nidophis	?	?	?	?	?	?	?	?	?	?	?	?	?	?	?	?
Patagoniophis	?	?	?	?	?	?	?	?	?	?	?	?	?	?	?	?
Nanowana schrenki	?	?	?	?	?	?	?	?	?	?	?	?	?	?	?	?
Powellophis	?	?	?	?	?	?	?	?	?	?	?	?	?	?	?	?
Vasuki indicus	?	?	?	?	?	?	?	?	?	?	?	?	?	?	?	?
Gigantophis garstini	?	?	?	?	?	?	?	?	?	?	?	?	?	?	?	?
Madtsoia pisdurensis	?	?	?	?	?	?	?	?	?	?	?	?	?	?	?	?
Gigantophis sp	?	?	?	?	?	?	?	?	?	?	?	?	?	?	?	?
Madtsoia camposi	?	?	?	?	?	?	?	?	?	?	?	?	?	?	?	?
Adinophis	?	?	?	?	?	?	?	?	?	?	?	?	?	?	?	?
Platyspondylophis	?	?	?	?	?	?	?	?	?	?	?	?	?	?	?	?
Menarana nosymena	?	?	?	?	?	?	?	?	?	?	?	?	?	?	?	?
Menarana laurasiae	?	?	?	?	?	?	?	?	?	?	?	?	?	?	?	?
Madtsoia madagascariensis	?	?	?	?	?	?	?	?	?	?	?	?	?	?	?	?
Madtsoia bai	?	?	?	?	?	?	?	?	?	?	?	?	?	?	?	?
Yurlunggur camfieldensis	0	0	?	?	1	?	0	0	1	0	?	?	0	2	?	0
Wonambi naracoortensis	0	?	?	?	?	?	?	?	?	0	?	?	?	?	?	?

Taxa/ Characters	177	178	179	180	181	182	183	184	185	186	187	188	189	190	191	192
Sanajeh indicus	?	?	?	?	?	?	?	?	?	?	?	?	?	?	?	?
Najash rionegrina	?	0	0	0	2	0	?	0	0	?	1	0	0	?	1	0
Nanowana godhelpi	?	?	?	?	?	?	?	?	?	?	?	?	?	?	?	?
Alamitophis tingamarra	?	?	?	?	?	?	?	?	?	?	?	?	?	?	?	?
Alamitophis elongatus	?	?	?	?	?	?	?	?	?	?	?	?	?	?	?	?
Nidophis	?	?	?	?	?	?	?	?	?	?	?	?	?	?	?	?
Patagoniophis	?	?	?	?	?	?	?	?	?	?	?	?	?	?	?	?
Nanowana schrenki	?	?	?	?	?	?	?	?	?	?	?	?	?	?	?	?
Powellophis	?	?	?	?	?	?	?	?	?	?	?	?	?	?	?	?
Vasuki indicus	?	?	?	?	?	?	?	?	?	?	?	?	?	?	?	?
Gigantophis garstini	?	?	?	?	?	?	?	?	?	?	?	?	?	?	?	?
Madtsoia pisdurensis	?	?	?	?	?	?	?	?	?	?	?	?	?	?	?	?
Gigantophis sp	?	?	?	?	?	?	?	?	?	?	?	?	?	?	?	?
Madtsoia camposi	?	?	?	?	?	?	?	?	?	?	?	?	?	?	?	?
Adinophis	?	?	?	?	?	?	?	?	?	?	?	?	?	?	?	?
Platyspondylophis	?	?	?	?	?	?	?	?	?	?	?	?	?	?	?	?
Menarana nosymena	?	?	?	?	?	?	?	?	?	?	?	?	?	?	?	?
Menarana laurasiae	?	?	?	?	?	?	?	?	?	?	?	?	?	?	?	?
Madtsoia madagascariensis	?	?	?	?	?	?	?	?	?	?	?	?	?	?	?	?
Madtsoia bai	?	?	?	?	?	?	?	?	?	?	?	?	?	?	?	?
Yurlunggur camfieldensis	?	0	?	0	?	?	0	0	0	?	1	0	0	?	0	0
Wonambi naracoortensis	?	?	?	?	?	?	?	?	?	?	?	?	?	?	?	?

Taxa/ Characters	193	194	195	196	197	198	199	200	201	202	203	204	205	206	207	208
Sanajeh indicus	?	?	?	0	?	?	1	0	0	0	?	0	0	2	0	0
Najash rionegrina	1	?	?	1	?	?	?	?	?	?	?	0	?	2	0	0
Nanowana godhelpi	?	?	?	?	?	?	?	?	?	?	?	?	?	?	?	?
Alamitophis tingamarra	?	?	?	?	?	?	?	?	?	?	?	?	?	?	?	?
Alamitophis elongatus	?	?	?	?	?	?	?	?	?	?	?	?	?	?	?	?
Nidophis	?	?	?	?	?	?	?	?	?	?	?	?	?	?	?	?
Patagoniophis	?	?	?	?	?	?	?	?	?	?	?	?	?	?	?	?
Nanowana schrenki	?	?	?	?	?	?	?	?	?	?	?	?	?	?	?	?
Powellophis	?	?	?	?	?	?	?	?	?	?	?	?	?	?	?	?
Vasuki indicus	?	?	?	?	?	?	?	?	?	?	?	?	?	?	?	?
Gigantophis garstini	?	?	?	?	?	?	?	?	?	?	?	?	?	?	?	?
Madtsoia pisdurensis	?	?	?	?	?	?	?	?	?	?	?	?	?	?	?	?
Gigantophis sp	?	?	?	?	?	?	?	?	?	?	?	?	?	?	?	?
Madtsoia camposi	?	?	?	?	?	?	?	?	?	?	?	?	?	?	?	?
Adinophis	?	?	?	?	?	?	?	?	?	?	?	?	?	?	?	?
Platyspondylophis	?	?	?	?	?	?	?	?	?	?	?	?	?	?	?	?
Menarana nosymena	?	?	?	?	?	?	?	?	?	?	?	?	?	?	?	?
Menarana laurasiae	?	?	?	?	?	?	?	?	?	?	?	?	?	?	?	?
Madtsoia madagascariensis	?	?	?	?	?	?	?	?	?	?	?	?	?	?	?	?
Madtsoia bai	?	?	?	?	?	?	?	?	?	?	?	?	?	?	?	?
Yurlunggur camfieldensis	1	?	?	1	?	?	?	?	?	?	?	0	?	?	0	3
Wonambi naracoortensis	1	?	?	?	?	?	?	?	?	?	?	0	?	2	0	3

Supplementary Dataset 2: continued

**Taxa/ Characters**	**209**	**210**	**211**	**212**	**213**	**214**	**215**	**216**	**217**	**218**	**219**	**220**	**221**	**222**	**223**	**224**
Sanajeh indicus	0	0	?	?	0	0	?	?	1	1	?	?	?	?	?	?
Najash rionegrina	0	0	?	0	0	0	0	1	?	?	1	0	1	3	0	0
Nanowana godhelpi	?	?	?	?	?	?	?	?	?	?	?	?	?	?	?	?
Alamitophis tingamarra	?	?	?	?	?	?	?	?	?	?	?	?	?	?	?	?
Alamitophis elongatus	?	?	?	?	?	?	?	?	?	?	?	?	?	?	?	?
Nidophis	?	?	?	?	?	?	?	?	?	?	?	?	?	?	?	?
Patagoniophis	?	?	?	?	?	?	?	?	?	?	?	?	?	?	?	?
Nanowana schrenki	?	?	?	?	?	?	?	?	?	?	?	?	?	?	?	?
Powellophis	?	?	?	?	?	?	?	?	?	?	?	?	?	?	?	?
Vasuki indicus	?	?	?	?	?	?	?	?	?	?	?	?	?	?	?	?
Gigantophis garstini	?	?	?	?	?	?	?	?	?	?	?	?	?	?	?	?
Madtsoia pisdurensis	?	?	?	?	?	?	?	?	?	?	?	?	?	?	?	?
Gigantophis sp	?	?	?	?	?	?	?	?	?	?	?	?	?	?	?	?
Madtsoia camposi	?	?	?	?	?	?	?	?	?	?	?	?	?	?	?	?
Adinophis	?	?	?	?	?	?	?	?	?	?	?	?	?	?	?	?
Platyspondylophis	?	?	?	?	?	?	?	?	?	?	?	?	?	?	?	?
Menarana nosymena	?	?	?	?	?	?	?	?	?	?	?	?	?	?	?	?
Menarana laurasiae	?	?	?	?	?	?	?	?	?	?	?	?	?	?	?	?
Madtsoia madagascariensis	?	?	?	?	?	?	?	?	?	?	?	?	?	?	?	?
Madtsoia bai	?	?	?	?	?	?	?	?	?	?	?	?	?	?	?	?
Yurlunggur camfieldensis	1	0	?	0	?	?	?	1	?	?	1	0	1	3	0	0
Wonambi naracoortensis	1	1	?	0	?	0	?	?	?	?	?	?	?	?	?	?

**Taxa/ Characters**	**225**	**226**	**227**	**228**	**229**	**230**	**231**	**232**	**233**	**234**	**235**	**236**	**237**	**238**	**239**	**240**
Sanajeh indicus	?	?	?	?	?	?	?	?	0	0	1	1	1	0	2	?
Najash rionegrina	?	0	?	?	?	0	0	0	0	1	?	1	1	?	2	?
Nanowana godhelpi	?	?	?	?	?	?	?	?	?	?	?	?	?	?	?	?
Alamitophis tingamarra	?	?	?	?	?	?	?	?	?	?	?	?	?	?	?	?
Alamitophis elongatus	?	?	?	?	?	?	?	?	?	?	?	?	?	?	?	?
Nidophis	?	?	?	?	?	?	?	?	?	?	?	?	?	?	?	?
Patagoniophis	?	?	?	?	?	?	?	?	?	?	?	?	?	?	?	?
Nanowana schrenki	?	?	?	?	?	?	?	?	?	?	?	?	?	?	?	?
Powellophis	?	?	?	?	?	?	?	?	?	?	?	?	?	?	?	?
Vasuki indicus	?	?	?	?	?	?	?	?	?	?	?	?	?	?	?	?
Gigantophis garstini	?	?	?	?	?	?	?	?	?	?	?	?	?	?	?	?
Madtsoia pisdurensis	?	?	?	?	?	?	?	?	?	?	?	?	?	?	?	?
Gigantophis sp	?	?	?	?	?	?	?	?	?	?	?	?	?	?	?	?
Madtsoia camposi	?	?	?	?	?	?	?	?	?	?	?	?	?	?	?	?
Adinophis	?	?	?	?	?	?	?	?	?	?	?	?	?	?	?	?
Platyspondylophis	?	?	?	?	?	?	?	?	?	?	?	?	?	?	?	?
Menarana nosymena	?	?	?	?	?	?	?	?	?	?	?	?	?	?	?	?
Menarana laurasiae	?	?	?	?	?	?	?	?	?	?	?	?	?	?	?	?
Madtsoia madagascariensis	?	?	?	?	?	?	?	?	?	?	?	?	?	?	?	?
Madtsoia bai	?	?	?	?	?	?	?	?	?	?	?	?	?	?	?	?
Yurlunggur camfieldensis	?	0	?	?	?	0	0	0	?	?	?	?	?	?	3	?
Wonambi naracoortensis	?	?	?	?	?	0	?	0	?	?	?	?	?	?	3	?

**Taxa/ Characters**	**241**	**242**	**243**	**244**	**245**	**246**	**247**	**248**	**249**	**250**	**251**	**252**	**253**	**254**	**255**	**256**
Sanajeh indicus	?	?	1	1	?	?	?	?	?	2	?	?	?	?	?	?
Najash rionegrina	0	?	1	1	1	?	?	?	0	?	?	?	0	?	?	0
Nanowana godhelpi	?	?	?	?	?	?	?	?	?	?	?	?	?	?	?	?
Alamitophis tingamarra	?	?	?	?	?	?	?	?	?	?	?	?	?	?	?	?
Alamitophis elongatus	?	?	?	?	?	?	?	?	?	?	?	?	?	?	?	?
Nidophis	?	?	?	?	?	?	?	?	?	?	?	?	?	?	?	?
Patagoniophis	?	?	?	?	?	?	?	?	?	?	?	?	?	?	?	?
Nanowana schrenki	?	?	?	?	?	?	?	?	?	?	?	?	?	?	?	?
Powellophis	?	?	?	?	?	?	?	?	?	?	?	?	?	?	?	?
Vasuki indicus	?	?	?	?	?	?	?	?	?	?	?	?	?	?	?	?
Gigantophis garstini	?	?	?	?	?	?	?	?	?	?	?	?	?	?	?	?
Madtsoia pisdurensis	?	?	?	?	?	?	?	?	?	?	?	?	?	?	?	?
Gigantophis sp	?	?	?	?	?	?	?	?	?	?	?	?	?	?	?	?
Madtsoia camposi	?	?	?	?	?	?	?	?	?	?	?	?	?	?	?	?
Adinophis	?	?	?	?	?	?	?	?	?	?	?	?	?	?	?	?
Platyspondylophis	?	?	?	?	?	?	?	?	?	?	?	?	?	?	?	?
Menarana nosymena	?	?	?	?	?	?	?	?	?	?	?	?	?	?	?	?
Menarana laurasiae	?	?	?	?	?	?	?	?	?	?	?	?	?	?	?	?
Madtsoia madagascariensis	?	?	?	?	?	?	?	?	?	?	?	?	?	?	?	?
Madtsoia bai	?	?	?	?	?	?	?	?	?	?	?	?	?	?	?	?
Yurlunggur camfieldensis	0	?	1	1	1	1	?	?	?	2	0	1	1	0	0	?
Wonambi naracoortensis	?	?	?	?	?	?	?	?	?	?	?	?	?	?	?	?

Supplementary Dataset 2: continued

Taxa/ Characters	257	258	259	260	261	262	263	264	265	266	267	268	269	270	271	272
Sanajeh indicus	?	?	?	?	?	?	?	?	?	?	?	?	?	?	?	?
Najash rionegrina	?	?	1	?	?	?	?	?	?	?	0	1	0	1	?	?
Nanowana godhelpi	?	?	?	?	?	?	?	?	?	?	?	?	?	?	?	?
Alamitophis tingamarra	?	?	?	?	?	?	?	?	?	?	?	?	?	?	?	?
Alamitophis elongatus	?	?	?	?	?	?	?	?	?	?	?	?	?	?	?	?
Nidophis	?	?	?	?	?	?	?	?	?	?	?	?	?	?	?	?
Patagoniophis	?	?	?	?	?	?	?	?	?	?	?	?	?	?	?	?
Nanowana schrenki	?	?	?	?	?	?	?	?	?	?	?	?	?	?	?	?
Powellophis	?	?	?	?	?	?	?	?	?	?	?	?	?	?	?	?
Vasuki indicus	?	?	?	?	?	?	?	?	?	?	?	?	?	?	?	?
Gigantophis garstini	?	?	?	?	?	?	?	?	?	?	?	?	?	?	?	?
Madtsoia pisdurensis	?	?	?	?	?	?	?	?	?	?	?	?	?	?	?	?
Gigantophis sp	?	?	?	?	?	?	?	?	?	?	?	?	?	?	?	?
Madtsoia camposi	?	?	?	?	?	?	?	?	?	?	?	?	?	?	?	?
Adinophis	?	?	?	?	?	?	?	?	?	?	?	?	?	?	?	?
Platyspondylophis	?	?	?	?	?	?	?	?	?	?	?	?	?	?	?	?
Menarana nosymena	?	?	?	?	?	?	?	?	?	?	?	?	?	?	?	?
Menarana laurasiae	?	?	?	?	?	?	?	?	?	?	?	?	?	?	?	?
Madtsoia madagascariensis	?	?	?	?	?	?	?	?	?	?	?	?	?	?	?	?
Madtsoia bai	?	?	?	?	?	?	?	?	?	?	?	?	?	?	?	?
Yurlunggur camfieldensis	?	?	?	?	?	?	?	?	1	0	0	1	0	1	?	?
Wonambi naracoortensis	?	?	?	?	?	?	?	?	?	?	?	?	?	?	?	?

Taxa/ Characters	273	274	275	276	277	278	279	280	281	282	283	284	285	286	287	288
Sanajeh indicus	?	?	?	?	?	?	?	?	?	?	?	?	?	?	?	?
Najash rionegrina	?	1	?	?	?	?	?	?	?	?	?	?	?	?	?	1
Nanowana godhelpi	?	?	?	?	?	?	?	?	?	?	?	?	?	?	?	?
Alamitophis tingamarra	?	?	?	?	?	?	?	?	?	?	?	?	?	?	?	?
Alamitophis elongatus	?	?	?	?	?	?	?	?	?	?	?	?	?	?	?	?
Nidophis	?	?	?	?	?	?	?	?	?	?	?	?	?	?	?	?
Patagoniophis	?	?	?	?	?	?	?	?	?	?	?	?	?	?	?	?
Nanowana schrenki	?	?	?	?	?	?	?	?	?	?	?	?	?	?	?	?
Powellophis	?	?	?	?	?	?	?	?	?	?	?	?	?	?	?	?
Vasuki indicus	?	?	?	?	?	?	?	?	?	?	?	?	?	?	?	?
Gigantophis garstini	?	?	?	?	?	?	?	?	?	?	?	?	?	?	?	?
Madtsoia pisdurensis	?	?	?	?	?	?	?	?	?	?	?	?	?	?	?	?
Gigantophis sp	?	?	?	?	?	?	?	?	?	?	?	?	?	?	?	?
Madtsoia camposi	?	?	?	?	?	?	?	?	?	?	?	?	?	?	?	?
Adinophis	?	?	?	?	?	?	?	?	?	?	?	?	?	?	?	?
Platyspondylophis	?	?	?	?	?	?	?	?	?	?	?	?	?	?	?	?
Menarana nosymena	?	?	?	?	?	?	?	?	?	?	?	?	?	?	?	?
Menarana laurasiae	?	?	?	?	?	?	?	?	?	?	?	?	?	?	?	?
Madtsoia madagascariensis	?	?	?	?	?	?	?	?	?	?	?	?	?	?	?	?
Madtsoia bai	?	?	?	?	?	?	?	?	?	?	?	?	?	?	?	?
Yurlunggur camfieldensis	?	2	0	1	2	?	2	?	1	?	?	?	0	?	?	1
Wonambi naracoortensis	?	?	?	?	?	?	?	?	?	?	?	?	?	?	?	?

Taxa/ Characters	289	290	291	292	293	294	295	296	297	298	299	300	301	302	303	304
Sanajeh indicus	0	?	0	?	?	?	?	?	0	?	0	0	?	2	0	0
Najash rionegrina	0	1	?	0	0	0	?	?	0	?	0	0	0	2	0	?
Nanowana godhelpi	?	?	?	?	?	?	?	?	0	?	?	?	?	?	?	?
Alamitophis tingamarra	?	?	?	?	?	?	?	?	?	?	?	?	?	?	?	?
Alamitophis elongatus	?	?	?	?	?	?	?	?	?	?	?	?	?	?	?	?
Nidophis	?	?	?	?	?	?	?	?	?	?	?	?	?	?	?	?
Patagoniophis	?	?	?	?	?	?	?	?	?	?	?	?	?	?	?	?
Nanowana schrenki	?	?	?	?	?	?	?	?	0	?	?	?	?	?	1	?
Powellophis	?	?	?	?	?	?	?	?	?	?	?	?	?	?	?	?
Vasuki indicus	?	?	?	?	?	?	?	?	?	?	?	?	?	?	?	?
Gigantophis garstini	?	?	?	?	?	?	?	?	?	?	?	?	?	?	?	?
Madtsoia pisdurensis	?	?	?	?	?	?	?	?	?	?	?	?	?	?	?	?
Gigantophis sp	?	?	?	?	?	?	?	?	?	?	?	?	?	?	?	?
Madtsoia camposi	?	?	?	?	?	?	?	?	0	?	?	?	?	?	1	?
Adinophis	?	?	?	?	?	?	?	?	?	?	?	?	?	?	?	?
Platyspondylophis	?	?	?	?	?	?	?	?	?	?	?	?	?	?	?	?
Menarana nosymena	?	?	?	?	?	?	?	?	?	?	?	?	?	?	?	?
Menarana laurasiae	?	?	?	?	?	?	?	?	?	?	?	?	?	?	?	?
Madtsoia madagascariensis	?	?	?	?	?	?	?	?	?	?	?	?	?	?	?	?
Madtsoia bai	?	?	?	?	?	?	?	?	?	?	?	?	?	?	?	?
Yurlunggur camfieldensis	0	?	?	3	?	0	0	1	0	?	?	?	?	4	1	0
Wonambi naracoortensis	?	?	?	?	0	?	?	?	0	?	?	?	0	4	1	0

Supplementary Dataset 2: continued

Taxa/ Characters	305	306	307	308	309	310	311	312	313	314	315	316	317	318	319	320
Sanajeh indicus	2	1	0	?	?	?	0	0	0	3	?	?	?	?	0	1
Najash rionegrina	0	?	?	?	?	?	0	0	0	3	?	0	?	?	?	?
Nanowana godhelpi	2	?	?	?	?	1	?	1	?	?	?	?	?	?	0	?
Alamitophis tingamarra	?	?	?	?	?	?	?	?	?	?	?	?	?	?	?	?
Alamitophis elongatus	?	?	?	?	?	?	?	?	?	?	?	?	?	?	?	?
Nidophis	?	?	?	?	?	?	?	?	?	?	?	?	?	?	?	?
Patagoniophis	?	?	?	?	?	?	?	?	?	?	?	?	?	?	?	?
Nanowana schrenki	2	?	1	?	?	?	?	0	?	?	?	?	?	?	0	?
Powellophis	?	?	?	?	?	?	?	?	?	?	?	?	?	?	?	?
Vasuki indicus	?	?	?	?	?	?	?	?	?	?	?	?	?	?	?	?
Gigantophis garstini	?	?	?	?	?	?	?	?	?	?	?	?	?	?	?	?
Madtsoia pisdurensis	?	?	?	?	?	?	?	?	?	?	?	?	?	?	?	?
Gigantophis sp	?	?	?	?	?	?	?	?	?	?	?	?	?	?	?	?
Madtsoia camposi	?	?	0	?	?	?	?	?	?	?	?	?	?	?	0	?
Adinophis	?	?	?	?	?	?	?	?	?	?	?	?	?	?	?	?
Platyspondylophis	?	?	?	?	?	?	?	?	?	?	?	?	?	?	?	?
Menarana nosymena	?	?	?	?	?	?	?	?	?	?	?	?	?	?	?	?
Menarana laurasiae	?	?	?	?	?	?	?	?	?	?	?	?	?	?	?	?
Madtsoia madagascariensis	?	?	?	?	?	?	?	?	?	?	?	?	?	?	?	?
Madtsoia bai	?	?	?	?	?	?	?	?	?	?	?	?	?	?	?	?
Yurlunggur camfieldensis	2	?	0	?	?	?	0	0	0	3	?	0	?	?	0	2
Wonambi naracoortensis	2	?	0	?	?	?	0	0	0	3	?	0	?	?	0	2

Taxa/ Characters	321	322	323	324	325	326	327	328	329	330	331	332	333	334	335	336
Sanajeh indicus	?	?	?	0	?	?	?	?	?	0	?	0	0	?	?	?
Najash rionegrina	?	?	1	0	?	?	0	0	?	0	0	0	0	?	?	?
Nanowana godhelpi	?	?	?	?	?	?	?	?	?	?	?	?	?	?	?	?
Alamitophis tingamarra	?	?	?	?	?	?	?	?	?	?	?	?	?	?	?	?
Alamitophis elongatus	?	?	?	?	?	?	?	?	?	?	?	?	?	?	?	?
Nidophis	?	?	?	?	?	?	?	?	?	?	?	?	?	?	?	?
Patagoniophis	?	?	?	?	?	?	?	?	?	?	?	?	?	?	?	?
Nanowana schrenki	?	?	?	?	?	?	?	?	?	?	?	?	?	?	?	?
Powellophis	?	?	?	?	?	?	?	?	?	?	?	?	?	?	?	?
Vasuki indicus	?	?	?	?	?	?	?	?	?	?	?	?	?	?	?	?
Gigantophis garstini	?	?	?	?	?	?	?	?	?	?	?	?	?	?	?	?
Madtsoia pisdurensis	?	?	?	?	?	?	?	?	?	?	?	?	?	?	?	?
Gigantophis sp	?	?	?	?	?	?	?	?	?	?	?	?	?	?	?	?
Madtsoia camposi	?	?	?	?	?	?	?	?	?	?	?	?	?	?	?	?
Adinophis	?	?	?	?	?	?	?	?	?	?	?	?	?	?	?	?
Platyspondylophis	?	?	?	?	?	?	?	?	?	?	?	?	?	?	?	?
Menarana nosymena	?	?	?	?	?	?	?	?	?	?	?	?	?	?	?	?
Menarana laurasiae	?	?	?	?	?	?	?	?	?	?	?	?	?	?	?	?
Madtsoia madagascariensis	?	?	?	?	?	?	?	?	?	?	?	?	?	?	?	?
Madtsoia bai	?	?	?	?	?	?	?	?	?	?	?	?	?	?	?	?
Yurlunggur camfieldensis	1	3	1	0	?	?	?	0	?	0	0	0	0	?	?	?
Wonambi naracoortensis	?	?	1	0	?	?	?	0	?	?	?	?	?	?	?	?

Taxa/ Characters	337	338	339	340	341	342	343	344	345	346	347	348	349	350	351	352
Sanajeh indicus	0	1	0	?	?	?	?	?	2	?	?	?	?	?	?	?
Najash rionegrina	?	?	?	0	?	1	0	1	0	?	0	0	0	?	0	0
Nanowana godhelpi	0	?	?	?	?	?	?	?	?	?	?	?	?	?	?	?
Alamitophis tingamarra	?	?	?	?	?	?	?	?	?	?	?	?	?	?	?	?
Alamitophis elongatus	?	?	?	?	?	?	?	?	?	?	?	?	?	?	?	?
Nidophis	?	?	?	?	?	?	?	?	?	?	?	?	?	?	?	?
Patagoniophis	?	?	?	?	?	?	?	?	?	?	?	?	?	?	?	?
Nanowana schrenki	?	?	?	?	?	?	?	?	?	?	?	?	?	?	?	?
Powellophis	?	?	?	?	?	?	?	?	?	?	?	?	?	?	?	?
Vasuki indicus	?	?	?	?	?	?	?	?	?	?	?	?	?	?	?	?
Gigantophis garstini	?	?	?	?	?	?	?	?	?	?	?	?	?	?	?	?
Madtsoia pisdurensis	?	?	?	?	?	?	?	?	?	?	?	?	?	?	?	?
Gigantophis sp	?	?	?	?	?	?	?	?	?	?	?	?	?	?	?	?
Madtsoia camposi	?	?	?	?	?	?	?	?	?	?	?	?	?	?	?	?
Adinophis	?	?	?	?	?	?	?	?	?	?	?	?	?	?	?	?
Platyspondylophis	?	?	?	?	?	?	?	?	?	?	?	?	?	?	?	?
Menarana nosymena	?	?	?	?	?	?	?	?	?	?	?	?	?	?	?	?
Menarana laurasiae	?	?	?	?	?	?	?	?	?	?	?	?	?	?	?	?
Madtsoia madagascariensis	?	?	?	?	?	?	?	?	?	?	?	?	?	?	?	?
Madtsoia bai	?	?	?	?	?	?	?	?	?	?	?	?	?	?	?	?
Yurlunggur camfieldensis	0	2	0	0	0	0	0	0	2	0	0	0	0	0	0	0
Wonambi naracoortensis	0	2	?	0	0	0	?	0	2	0	0	0	0	?	0	?

Supplementary Dataset 2: continued

Taxa/ Characters	353	354	355	356	357	358	359	360	361	362	363	364	365	366	367	368
Sanajeh indicus	?	?	?	?	?	?	?	?	?	0	1	?	?	?	?	?
Najash rionegrina	?	1	?	1	?	0	0	?	0	0	1	?	?	?	?	?
Nanowana godhelpi	?	?	?	?	?	?	?	?	?	?	?	?	?	?	?	?
Alamitophis tingamarra	?	?	?	?	?	?	?	?	?	?	?	?	?	?	?	?
Alamitophis elongatus	?	?	?	?	?	?	?	?	?	?	?	?	?	?	?	?
Nidophis	?	?	?	?	?	?	?	?	?	?	?	?	?	?	?	?
Patagoniophis	?	?	?	?	?	?	?	?	?	?	?	?	?	?	?	?
Nanowana schrenki	?	?	?	?	?	?	?	?	?	?	?	?	?	?	?	?
Powellophis	?	?	?	?	?	?	?	?	?	?	?	?	?	?	?	?
Vasuki indicus	?	?	?	?	?	?	?	?	?	?	?	?	?	?	?	?
Gigantophis garstini	?	?	?	?	?	?	?	?	?	?	?	?	?	?	?	?
Madtsoia pisdurensis	?	?	?	?	?	?	?	?	?	?	?	?	?	?	?	?
Gigantophis sp	?	?	?	?	?	?	?	?	?	?	?	?	?	?	?	?
Madtsoia camposi	?	?	?	?	?	?	?	?	?	?	?	?	?	?	?	?
Adinophis	?	?	?	?	?	?	?	?	?	?	?	?	?	?	?	?
Platyspondylophis	?	?	?	?	?	?	?	?	?	?	?	?	?	?	?	?
Menarana nosymena	?	?	?	?	?	?	?	?	?	?	?	?	?	?	?	?
Menarana laurasiae	?	?	?	?	?	?	?	?	?	?	?	?	?	?	?	?
Madtsoia madagascariensis	?	?	?	?	?	?	?	?	?	?	?	?	?	?	?	?
Madtsoia bai	?	?	?	?	?	?	?	?	?	?	?	?	?	?	?	?
Yurlunggur camfieldensis	0	1	?	2	0	0	0	?	0	0	?	?	?	?	?	?
Wonambi naracoortensis	0	1	?	2	?	0	?	?	?	?	?	?	?	?	?	?

Taxa/ Characters	369	370	371	372	373	374	375	376	377	378	379	380	381	382	383	384
Sanajeh indicus	0	0	?	?	2	?	?	0	?	1	?	?	?	?	0	1
Najash rionegrina	0	0	0	1	2	0	0	0	?	1	?	?	?	?	0	1
Nanowana godhelpi	?	?	?	?	?	?	?	?	?	?	?	?	?	?	?	?
Alamitophis tingamarra	?	?	?	?	?	?	?	?	?	?	?	?	?	?	?	?
Alamitophis elongatus	?	?	?	?	?	?	?	?	?	?	?	?	?	?	?	?
Nidophis	?	?	?	?	?	?	?	?	?	?	?	?	?	?	?	?
Patagoniophis	?	?	?	?	?	?	?	?	?	?	?	?	?	?	?	?
Nanowana schrenki	?	?	?	?	?	?	?	?	?	?	?	?	?	?	?	?
Powellophis	?	?	?	?	?	?	?	?	?	?	?	?	?	?	?	?
Vasuki indicus	?	?	?	?	?	?	?	?	?	?	?	?	?	?	?	?
Gigantophis garstini	?	?	?	?	?	?	?	?	?	?	?	?	?	?	?	?
Madtsoia pisdurensis	?	?	?	?	?	?	?	?	?	?	?	?	?	?	?	?
Gigantophis sp	?	?	?	?	?	?	?	?	?	?	?	?	?	?	?	?
Madtsoia camposi	?	?	?	?	?	?	?	?	?	?	?	?	?	?	?	?
Adinophis	?	?	?	?	?	?	?	?	?	?	?	?	?	?	?	?
Platyspondylophis	?	?	?	?	?	?	?	?	?	?	?	?	?	?	?	?
Menarana nosymena	?	?	?	?	?	?	?	?	?	?	?	?	?	?	?	?
Menarana laurasiae	?	?	?	?	?	?	?	?	?	?	?	?	?	?	?	?
Madtsoia madagascariensis	?	?	?	?	?	?	?	?	?	?	?	?	?	?	?	?
Madtsoia bai	?	?	?	?	?	?	?	?	?	?	?	?	?	?	?	?
Yurlunggur camfieldensis	0	?	?	?	?	?	?	?	?	1	?	?	?	?	1	1
Wonambi naracoortensis	0	?	?	?	?	?	?	?	?	1	?	?	?	?	1	1

Taxa/ Characters	385	386	387	388	389	390	391	392	393	394	395	396	397	398	399	400
Sanajeh indicus	0	0	?	?	?	0	0	0	?	0	?	?	?	?	?	1
Najash rionegrina	0	0	1	0	?	0	0	?	?	0	?	?	0	1	0	1
Nanowana godhelpi	?	?	?	?	?	?	?	?	?	?	?	?	?	?	?	?
Alamitophis tingamarra	?	?	?	?	?	?	?	?	?	?	?	?	?	?	?	?
Alamitophis elongatus	?	?	?	?	?	?	?	?	?	?	?	?	?	?	?	?
Nidophis	?	?	?	?	?	?	?	?	?	?	?	?	?	?	?	?
Patagoniophis	?	?	?	?	?	?	?	?	?	?	?	?	?	?	?	?
Nanowana schrenki	?	?	?	?	?	?	?	?	?	?	?	?	?	?	?	?
Powellophis	?	?	?	?	?	?	?	?	?	?	?	?	?	?	?	?
Vasuki indicus	?	?	?	?	?	?	?	?	?	?	?	?	?	?	?	?
Gigantophis garstini	?	?	?	?	?	?	?	?	?	?	?	?	?	?	?	?
Madtsoia pisdurensis	?	?	?	?	?	?	?	?	?	?	?	?	?	?	?	?
Gigantophis sp	?	?	?	?	?	?	?	?	?	?	?	?	?	?	?	?
Madtsoia camposi	?	?	?	?	?	?	?	?	?	?	?	?	?	?	?	?
Adinophis	?	?	?	?	?	?	?	?	?	?	?	?	?	?	?	?
Platyspondylophis	?	?	?	?	?	?	?	?	?	?	?	?	?	?	?	?
Menarana nosymena	?	?	0	?	0	0	0	0	?	?	?	?	?	?	?	?
Menarana laurasiae	?	?	?	?	?	?	?	?	?	?	?	?	?	?	?	?
Madtsoia madagascariensis	?	?	?	?	?	?	?	?	?	?	?	?	?	?	?	?
Madtsoia bai	?	?	?	?	?	?	?	?	?	?	?	?	?	?	?	?
Yurlunggur camfieldensis	?	0	?	0	?	0	0	?	0	?	?	?	?	1	0	1
Wonambi naracoortensis	?	0	?	0	?	0	0	?	?	?	?	?	?	?	0	1

Supplementary Dataset 2: continued

Taxa/ Characters	401	402	403	404	405	406	407	408	409	410	411	412	413	414	415	416
Sanajeh indicus	0	0	1	?	?	0	?	?	?	?	?	?	?	?	?	?
Najash rionegrina	0	0	?	1	0	0	0	0	0	0	0	?	?	1	0	0
Nanowana godhelpi	?	?	?	?	?	?	?	?	?	?	?	?	?	?	?	?
Alamitophis tingamarra	?	?	?	?	?	?	?	?	?	?	?	?	?	?	?	?
Alamitophis elongatus	?	?	?	?	?	?	?	?	?	?	?	?	?	?	?	?
Nidophis	?	?	?	?	?	?	?	?	?	?	?	?	?	?	?	?
Patagoniophis	?	?	?	?	?	?	?	?	?	?	?	?	?	?	?	?
Nanowana schrenki	?	?	?	?	?	?	?	?	?	?	?	?	?	?	?	?
Powellophis	?	?	?	?	?	?	?	?	?	?	?	?	?	?	?	?
Vasuki indicus	?	?	?	?	?	?	?	?	?	?	?	?	?	?	?	?
Gigantophis garstini	?	?	?	?	?	?	?	?	?	?	?	?	?	?	?	?
Madtsoia pisdurensis	?	?	?	?	?	?	?	?	?	?	?	?	?	?	?	?
Gigantophis sp	?	?	?	?	?	?	?	?	?	?	?	?	?	?	?	?
Madtsoia camposi	?	?	?	?	?	?	?	?	?	?	?	?	?	?	?	?
Adinophis	?	?	?	?	?	?	?	?	?	?	?	?	?	?	?	?
Platyspondylophis	?	?	?	?	?	?	?	?	?	?	?	?	?	?	?	?
Menarana nosymena	?	?	?	1	?	?	?	?	?	?	?	?	?	?	?	?
Menarana laurasiae	?	?	?	?	?	?	?	?	?	?	?	?	?	?	?	?
Madtsoia madagascariensis	?	?	?	?	?	?	?	?	?	?	?	?	?	?	?	?
Madtsoia bai	?	?	?	?	?	?	?	?	?	?	?	?	?	?	?	?
Yurlunggur camfieldensis	1	0	1	1	0	1	0	?	0	?	?	?	?	1	1	1
Wonambi naracoortensis	?	0	1	1	0	1	?	?	0	?	?	?	?	1	1	1

Taxa/ Characters	417	418	419	420	421	422	423	424	425	426	427	428	429	430	431	432
Sanajeh indicus	?	?	?	?	?	?	?	?	0	?	?	?	?	?	?	?
Najash rionegrina	0	0	?	?	?	?	?	?	0	?	?	?	1	1	?	?
Nanowana godhelpi	?	?	?	?	?	?	?	?	?	?	?	?	?	?	?	?
Alamitophis tingamarra	?	?	?	?	?	?	?	?	?	?	?	?	?	?	?	?
Alamitophis elongatus	?	?	?	?	?	?	?	?	?	?	?	?	?	?	?	?
Nidophis	?	?	?	?	?	?	?	?	?	?	?	?	?	?	?	?
Patagoniophis	?	?	?	?	?	?	?	?	?	?	?	?	?	?	?	?
Nanowana schrenki	?	?	?	?	?	?	?	?	?	?	?	?	?	?	?	?
Powellophis	?	?	?	?	?	?	?	?	?	?	?	?	?	?	?	?
Vasuki indicus	?	?	?	?	?	?	?	?	?	?	?	?	?	?	?	?
Gigantophis garstini	?	?	?	?	?	?	?	?	?	?	?	?	?	?	?	?
Madtsoia pisdurensis	?	?	?	?	?	?	?	?	?	?	?	?	?	?	?	?
Gigantophis sp	?	?	?	?	?	?	?	?	?	?	?	?	?	?	?	?
Madtsoia camposi	?	?	?	?	?	?	?	?	?	?	?	?	?	?	?	?
Adinophis	?	?	?	?	?	?	?	?	?	?	?	?	?	?	?	?
Platyspondylophis	?	?	?	?	?	?	?	?	?	?	?	?	?	?	?	?
Menarana nosymena	?	?	?	?	?	?	?	?	?	?	?	?	?	?	?	?
Menarana laurasiae	?	?	?	?	?	?	?	?	?	?	?	?	?	?	?	?
Madtsoia madagascariensis	?	?	?	?	?	?	?	?	?	?	?	?	?	?	?	?
Madtsoia bai	?	?	?	?	?	?	?	?	?	?	?	?	?	?	?	?
Yurlunggur camfieldensis	1	0	?	1	0	?	?	?	1	?	?	?	?	?	?	?
Wonambi naracoortensis	1	?	?	1	0	?	?	?	1	?	?	?	?	?	0	?

Taxa/ Characters	433	434	435	436	437	438	439	440	441	442	443	444	445	446	447	448
Sanajeh indicus	?	?	?	?	?	?	?	?	0	0	?	?	?	1	?	?
Najash rionegrina	0	?	0	0	0	0	0	0	?	0	?	?	?	?	2	0
Nanowana godhelpi	?	?	?	?	?	?	?	?	?	?	?	?	?	?	?	?
Alamitophis tingamarra	?	?	?	?	?	?	?	?	?	?	?	?	?	?	?	?
Alamitophis elongatus	?	?	?	?	?	?	?	?	?	?	?	?	?	?	?	?
Nidophis	?	?	?	?	?	?	?	?	?	?	?	?	?	?	?	?
Patagoniophis	?	?	?	?	?	?	?	?	?	?	?	?	?	?	?	?
Nanowana schrenki	?	?	?	?	?	?	?	?	?	?	?	?	?	?	?	?
Powellophis	?	?	?	?	?	?	?	?	?	?	?	?	?	?	?	?
Vasuki indicus	?	?	?	?	?	?	?	?	?	?	?	?	?	?	?	?
Gigantophis garstini	?	?	?	?	?	?	?	?	?	?	?	?	?	?	?	?
Madtsoia pisdurensis	?	?	?	?	?	?	?	?	?	?	?	?	?	?	?	?
Gigantophis sp	?	?	?	?	?	?	?	?	?	?	?	?	?	?	?	?
Madtsoia camposi	?	?	?	?	?	?	?	?	?	?	?	?	?	?	?	?
Adinophis	?	?	?	?	?	?	?	?	?	?	?	?	?	?	?	?
Platyspondylophis	?	?	?	?	?	?	?	?	?	?	?	?	?	?	?	?
Menarana nosymena	?	?	?	?	?	?	?	1	?	?	?	?	?	?	?	?
Menarana laurasiae	?	?	?	?	?	?	?	?	?	?	?	?	?	?	?	?
Madtsoia madagascariensis	?	?	?	?	?	?	?	?	?	?	?	?	?	?	?	?
Madtsoia bai	?	?	?	?	?	?	?	?	?	?	?	?	?	?	?	?
Yurlunggur camfieldensis	0	?	?	0	1	1	1	?	?	1	?	?	?	?	?	1
Wonambi naracoortensis	0	?	?	0	1	1	1	?	0	1	?	?	?	1	?	1

Supplementary Dataset 2: continued

Taxa/ Characters	449	450	451	452	453	454	455	456	457	458	459	460	461	462	463	464
Sanajeh indicus	?	?	1	?	?	0	1	?	0	0	?	0	1	?	1	1
Najash rionegrina	1	?	1	0	0	0	1	1	0	0	?	0	1	1	1	1
Nanowana godhelpi	?	?	?	?	?	?	?	?	?	1	0	0	1	0	?	?
Alamitophis tingamarra	?	?	?	?	?	?	?	?	?	?	?	?	?	?	?	?
Alamitophis elongatus	?	?	?	?	?	?	?	?	?	?	?	?	?	?	?	?
Nidophis	?	?	?	?	?	?	?	?	?	?	?	?	?	?	?	?
Patagoniophis	?	?	?	?	?	?	?	?	?	?	?	?	?	?	?	?
Nanowana schrenki	?	?	?	?	?	?	?	?	?	1	0	0	1	0	?	?
Powellophis	?	?	?	?	?	?	?	?	?	?	0	0	1	0	?	?
Vasuki indicus	?	?	?	?	?	?	?	?	?	?	?	?	?	?	?	?
Gigantophis garstini	?	?	?	?	?	?	?	?	?	?	?	?	?	?	?	?
Madtsoia pisdurensis	?	?	?	?	?	?	?	?	?	?	?	?	?	?	?	?
Gigantophis sp	?	?	?	?	?	?	?	?	?	?	?	?	?	?	?	?
Madtsoia camposi	?	?	?	?	?	?	?	?	?	?	?	?	?	0	?	?
Adinophis	?	?	?	?	?	?	?	?	?	?	?	?	?	?	?	?
Platyspondylophis	?	?	?	?	?	?	?	?	?	?	?	?	?	?	?	?
Menarana nosymena	0	?	1	?	?	?	0	?	?	?	?	?	?	?	?	?
Menarana laurasiae	?	?	?	?	?	?	?	?	?	?	?	?	?	?	?	?
Madtsoia madagascariensis	?	?	?	?	?	?	?	?	?	?	?	?	?	?	?	?
Madtsoia bai	?	?	?	?	?	?	?	?	?	?	?	?	?	?	?	?
Yurlunggur camfieldensis	?	?	1	?	?	1	1	?	1	1	?	0	?	?	?	?
Wonambi naracoortensis	?	?	1	?	?	1	1	?	1	1	?	0	1	?	1	?

Taxa/ Characters	465	466	467	468	469	470	471	472	473	474	475	476	477	478	479	480
Sanajeh indicus	?	1	2	1	?	0	?	?	?	?	?	?	?	?	1	?
Najash rionegrina	1	1	2	2	0	0	0	0	0	0	?	?	1	0	1	?
Nanowana godhelpi	?	1	?	3	1	1	?	?	?	?	?	?	?	?	?	?
Alamitophis tingamarra	?	?	?	?	?	?	?	?	?	?	?	?	?	?	?	?
Alamitophis elongatus	?	?	?	?	?	?	?	?	?	?	?	?	?	?	?	?
Nidophis	?	?	?	?	?	?	?	?	?	?	?	?	?	?	?	?
Patagoniophis	?	?	?	?	?	?	?	?	?	?	?	?	?	?	?	?
Nanowana schrenki	?	1	2	3	2	1	?	?	?	?	?	?	?	?	?	?
Powellophis	?	?	?	?	?	?	?	?	?	?	?	?	?	?	?	?
Vasuki indicus	?	?	?	?	?	?	?	?	?	?	?	?	?	?	?	?
Gigantophis garstini	?	?	?	?	?	?	?	?	?	?	?	?	?	?	?	?
Madtsoia pisdurensis	?	?	?	?	?	?	?	?	?	?	?	?	?	?	?	?
Gigantophis sp	?	?	?	?	?	?	?	?	?	?	?	?	?	?	?	?
Madtsoia camposi	?	1	2	3	2	1	?	0	?	?	?	?	?	?	1	?
Adinophis	?	?	?	?	?	?	?	?	?	?	?	?	?	?	?	?
Platyspondylophis	?	?	?	?	?	?	?	?	?	?	?	?	?	?	?	?
Menarana nosymena	?	?	?	?	?	?	?	?	?	?	?	?	?	?	?	?
Menarana laurasiae	?	?	?	?	?	?	?	?	?	?	?	?	?	?	?	?
Madtsoia madagascariensis	?	?	?	?	?	?	?	?	?	?	?	?	?	?	?	?
Madtsoia bai	?	?	?	?	?	?	?	?	?	?	?	?	?	?	?	?
Yurlunggur camfieldensis	0	1	2	2	?	1	?	0	?	?	?	?	?	?	1	?
Wonambi naracoortensis	0	1	2	1	?	1	?	?	?	?	?	?	1	?	1	?

Taxa/ Characters	481	482	483	484	485	486	487	488	489	490	491	492	493	494	495	496
Sanajeh indicus	?	0	?	?	?	?	?	?	1	?	?	?	0	?	?	?
Najash rionegrina	0	0	0	1	0	0	1	1	1	?	1	?	0	1	1	1
Nanowana godhelpi	?	0	?	?	?	?	?	?	?	?	?	?	?	?	?	?
Alamitophis tingamarra	?	?	?	?	?	?	?	?	?	?	?	?	?	?	?	?
Alamitophis elongatus	?	?	?	?	?	?	?	?	?	?	?	?	?	?	?	?
Nidophis	?	?	?	?	?	?	?	?	?	?	?	?	?	?	?	?
Patagoniophis	?	?	?	?	?	?	?	?	?	?	?	?	?	?	?	?
Nanowana schrenki	?	?	?	?	?	?	?	?	?	?	?	?	?	?	?	?
Powellophis	?	?	?	?	?	?	?	?	?	?	?	?	?	?	?	?
Vasuki indicus	?	?	?	?	?	?	?	?	?	?	?	?	?	?	?	?
Gigantophis garstini	?	?	?	?	?	?	?	?	?	?	?	?	?	?	?	?
Madtsoia pisdurensis	?	?	?	?	?	?	?	?	?	?	?	?	?	?	?	?
Gigantophis sp	?	?	?	?	?	?	?	?	?	?	?	?	?	?	?	?
Madtsoia camposi	?	?	?	?	?	?	?	?	?	?	?	?	?	?	?	?
Adinophis	?	?	?	?	?	?	?	?	?	?	?	?	?	?	?	?
Platyspondylophis	?	?	?	?	?	?	?	?	?	?	?	?	?	?	?	?
Menarana nosymena	?	?	?	?	?	?	?	?	?	?	?	?	?	?	?	?
Menarana laurasiae	?	?	?	?	?	?	?	?	?	?	?	?	?	?	?	?
Madtsoia madagascariensis	?	?	?	?	?	?	?	?	?	?	?	?	?	?	?	?
Madtsoia bai	?	?	?	?	?	?	?	?	?	?	?	?	?	?	?	?
Yurlunggur camfieldensis	?	?	?	?	?	0	?	?	1	?	?	?	0	?	1	?
Wonambi naracoortensis	1	?	?	?	?	?	?	?	?	?	?	?	?	?	1	?

Supplementary Dataset 2: continued

Taxa/ Characters	497	498	499	500	501	502	503	504	505	506	507	508	509	510	511	512
Sanajeh indicus	0	?	?	0	1	?	0	2	0	?	1	?	?	?	1	?
Najash rionegrina	0	?	?	0	1	0	0	2	?	?	1	0	?	?	1	0
Nanowana godhelpi	?	?	?	?	?	?	?	?	?	?	?	?	?	?	?	?
Alamitophis tingamarra	?	?	?	?	?	?	?	?	?	?	?	?	?	?	?	?
Alamitophis elongatus	?	?	?	?	?	?	?	?	?	?	?	?	?	?	?	?
Nidophis	?	?	?	?	?	?	?	?	?	?	?	?	?	?	?	?
Patagoniophis	?	?	?	?	?	?	?	?	?	?	?	?	?	?	?	?
Nanowana schrenki	?	?	?	?	?	?	?	?	?	?	?	?	?	?	?	?
Powellophis	?	?	?	?	?	?	?	?	?	?	?	?	?	?	?	?
Vasuki indicus	?	?	?	?	?	?	?	?	?	?	?	?	?	?	?	?
Gigantophis garstini	?	?	?	?	?	?	?	?	?	?	?	?	?	?	?	?
Madtsoia pisdurensis	?	?	?	?	?	?	?	?	?	?	?	?	?	?	?	?
Gigantophis sp	?	?	?	?	?	?	?	?	?	?	?	?	?	?	?	?
Madtsoia camposi	?	?	?	?	?	?	?	?	?	?	?	?	?	?	?	?
Adinophis	?	?	?	?	?	?	?	?	?	?	?	?	?	?	?	?
Platyspondylophis	?	?	?	?	?	?	?	?	?	?	?	?	?	?	?	?
Menarana nosymena	?	?	?	?	?	?	?	?	?	?	?	?	?	?	?	?
Menarana laurasiae	?	?	?	?	?	?	?	?	?	?	?	?	?	?	?	?
Madtsoia madagascariensis	?	?	?	?	?	?	?	?	?	?	?	?	?	?	?	?
Madtsoia bai	?	?	?	?	?	?	?	?	?	?	?	?	?	?	?	?
Yurlunggur camfieldensis	0	?	?	?	?	?	?	?	?	?	?	?	?	?	?	?
Wonambi naracoortensis	?	?	?	?	?	?	?	?	?	?	?	?	?	?	1	?

Taxa/ Characters	513	514	515	516	517	518	519	520	521	522	523	524	525	526	527	528
Sanajeh indicus	?	?	?	?	0	0	1	?	?	?	?	?	?	0	?	?
Najash rionegrina	?	?	0	0	0	0	1	?	1	0	0	?	1	0	?	0
Nanowana godhelpi	?	?	?	?	?	?	?	?	?	?	?	?	1	1	1	?
Alamitophis tingamarra	?	?	?	?	?	?	?	?	?	?	?	?	?	?	?	?
Alamitophis elongatus	?	?	?	?	?	?	?	?	?	?	?	?	?	?	?	?
Nidophis	?	?	?	?	?	?	?	?	?	?	?	?	?	?	?	?
Patagoniophis	?	?	?	?	?	?	?	?	?	?	?	?	?	?	?	?
Nanowana schrenki	?	?	?	?	?	?	?	?	?	?	?	?	?	?	?	?
Powellophis	?	?	?	?	?	?	?	?	?	?	?	?	?	?	?	?
Vasuki indicus	?	?	?	?	?	?	?	?	?	?	?	?	?	?	?	?
Gigantophis garstini	?	?	?	?	?	?	?	?	?	?	?	?	?	?	?	?
Madtsoia pisdurensis	?	?	?	?	?	?	?	?	?	?	?	?	?	?	?	?
Gigantophis sp	?	?	?	?	?	?	?	?	?	?	?	?	?	?	?	?
Madtsoia camposi	?	?	?	?	?	?	?	?	?	?	?	?	?	?	?	?
Adinophis	?	?	?	?	?	?	?	?	?	?	?	?	?	?	?	?
Platyspondylophis	?	?	?	?	?	?	?	?	?	?	?	?	?	?	?	?
Menarana nosymena	?	?	?	?	?	?	?	?	?	?	?	?	?	?	?	?
Menarana laurasiae	?	?	?	?	?	?	?	?	?	?	?	?	?	?	?	?
Madtsoia madagascariensis	?	?	?	?	?	?	?	?	?	?	?	?	?	?	?	?
Madtsoia bai	?	?	?	?	?	?	?	?	?	?	?	?	?	?	?	?
Yurlunggur camfieldensis	?	?	?	?	?	?	?	?	?	?	?	?	?	0	?	?
Wonambi naracoortensis	1	1	0	0	0	0	1	?	1	0	0	?	?	0	?	0

Taxa/ Characters	529	530	531	532	533	534	535	536	537	538	539	540	541	542	543	544
Sanajeh indicus	?	?	?	?	?	?	?	?	?	?	?	?	2	3	?	0
Najash rionegrina	0	0	0	0	?	?	0	0	?	?	?	?	2	?	?	?
Nanowana godhelpi	?	0	0	?	1	?	?	?	?	?	?	?	?	?	?	?
Alamitophis tingamarra	?	?	?	?	?	?	?	?	?	?	?	?	?	?	?	?
Alamitophis elongatus	?	?	?	?	?	?	?	?	?	?	?	?	?	?	?	?
Nidophis	?	?	?	?	?	?	?	?	?	?	?	?	?	?	?	?
Patagoniophis	?	?	?	?	?	?	?	?	?	?	?	?	?	?	?	?
Nanowana schrenki	?	?	?	?	?	?	?	?	?	?	?	?	?	?	?	1
Powellophis	?	?	?	?	?	?	?	?	?	?	?	?	?	?	?	?
Vasuki indicus	?	?	?	?	?	?	?	?	?	?	?	?	?	?	?	?
Gigantophis garstini	?	?	?	?	?	?	?	?	?	?	?	?	?	?	?	?
Madtsoia pisdurensis	?	?	?	?	?	?	?	?	?	?	?	?	?	?	?	?
Gigantophis sp	?	?	?	?	?	?	?	?	?	?	?	?	?	?	?	?
Madtsoia camposi	?	?	?	?	?	?	?	?	?	?	?	?	?	?	?	?
Adinophis	?	?	?	?	?	?	?	?	?	?	?	?	?	?	?	?
Platyspondylophis	?	?	?	?	?	?	?	?	?	?	?	?	?	?	?	?
Menarana nosymena	?	?	?	?	?	?	?	?	?	?	?	?	?	?	?	?
Menarana laurasiae	?	?	?	?	?	?	?	?	?	?	?	?	?	?	?	?
Madtsoia madagascariensis	?	?	?	?	?	?	?	?	?	?	?	?	?	?	?	?
Madtsoia bai	?	?	?	?	?	?	?	?	?	?	?	?	?	?	?	?
Yurlunggur camfieldensis	?	?	?	?	?	?	?	?	?	?	?	?	2	3	0	?
Wonambi naracoortensis	1	0	?	?	?	?	0	?	?	?	0	?	2	3	0	0

Supplementary Dataset 2: continued

Taxa/ Characters	545	546	547	548	549	550	551	552	553	554	555	556	557	558	559	560
Sanajeh indicus	0	2	1	?	?	1	?	1	0	0	0	0	0	1	1	1
Najash rionegrina	0	?	?	?	2	?	?	?	?	0	0	?	?	1	?	?
Nanowana godhelpi	?	?	?	?	?	?	?	?	?	?	?	?	?	?	?	?
Alamitophis tingamarra	?	?	?	?	?	?	?	?	?	?	?	?	?	?	?	?
Alamitophis elongatus	?	?	?	?	?	?	?	?	?	?	?	?	?	?	?	?
Nidophis	?	?	?	?	?	?	?	?	?	?	?	?	?	?	?	?
Patagoniophis	?	?	?	?	?	?	?	?	?	?	?	?	?	?	?	?
Nanowana schrenki	?	?	?	?	?	?	1	1	1	?	?	?	?	?	?	?
Powellophis	?	?	?	?	?	?	?	?	?	?	?	?	?	?	?	?
Vasuki indicus	?	?	?	?	?	?	?	?	?	?	?	?	?	?	?	?
Gigantophis garstini	?	?	?	?	?	?	?	?	?	?	?	?	?	?	?	?
Madtsoia pisdurensis	?	?	?	?	?	?	?	?	?	?	?	?	?	?	?	?
Gigantophis sp	?	?	?	?	?	?	?	?	?	?	?	?	?	?	?	?
Madtsoia camposi	?	?	?	?	?	?	?	?	0	?	?	?	?	?	?	?
Adinophis	?	?	?	?	?	?	?	?	?	?	?	?	?	?	?	?
Platyspondylophis	?	?	?	?	?	?	?	?	?	?	?	?	?	?	?	?
Menarana nosymena	?	?	?	?	?	?	?	?	?	?	?	?	?	?	?	?
Menarana laurasiae	?	?	?	?	?	?	?	?	?	?	?	?	?	?	?	?
Madtsoia madagascariensis	?	?	?	?	?	?	?	?	?	?	?	?	?	?	?	?
Madtsoia bai	?	?	?	?	?	?	?	?	?	?	?	?	?	?	?	?
Yurlunggur camfieldensis	0	?	0	?	?	1	?	1	1	1	0	0	0	1	1	1
Wonambi naracoortensis	0	?	?	?	?	1	?	1	1	1	0	0	0	1	1	1

Taxa/ Characters	561	562	563	564	565	566	567	568	569	570	571	572	573	574	575	576
Sanajeh indicus	?	?	1	?	0	?	?	0	?	0	1	?	0	0	0	0
Najash rionegrina	?	?	1	?	?	1	?	0	?	?	?	?	?	?	?	?
Nanowana godhelpi	?	?	?	?	?	?	?	0	?	?	?	?	?	?	?	?
Alamitophis tingamarra	?	?	?	?	?	?	?	?	?	?	?	?	?	?	?	?
Alamitophis elongatus	?	?	?	?	?	?	?	?	?	?	?	?	?	?	?	?
Nidophis	?	?	?	?	?	?	?	?	?	?	?	?	?	?	?	?
Patagoniophis	?	?	?	?	?	?	?	?	?	?	?	?	?	?	?	?
Nanowana schrenki	?	?	?	?	?	?	?	1	?	?	?	?	?	?	?	?
Powellophis	?	?	?	?	?	?	?	?	?	?	?	?	?	?	?	?
Vasuki indicus	?	?	?	?	?	?	?	?	?	?	?	?	?	?	?	?
Gigantophis garstini	?	?	?	?	?	?	?	?	?	?	?	?	?	?	?	?
Madtsoia pisdurensis	?	?	?	?	?	?	?	?	?	?	?	?	?	?	?	?
Gigantophis sp	?	?	?	?	?	?	?	?	?	?	?	?	?	?	?	?
Madtsoia camposi	?	?	2	?	?	?	?	?	?	?	?	?	?	0	?	?
Adinophis	?	?	?	?	?	?	?	?	?	?	?	?	?	?	?	?
Platyspondylophis	?	?	?	?	?	?	?	?	?	?	?	?	?	?	?	?
Menarana nosymena	?	?	?	?	?	?	?	?	?	?	?	?	?	?	?	?
Menarana laurasiae	?	?	?	?	?	?	?	?	?	?	?	?	?	?	?	?
Madtsoia madagascariensis	?	?	?	?	?	?	?	?	?	?	?	?	?	?	?	?
Madtsoia bai	?	?	?	?	?	?	?	?	?	?	?	?	?	?	?	?
Yurlunggur camfieldensis	?	?	2	?	0	1	?	0	?	1	0	?	0	0	0	0
Wonambi naracoortensis	?	?	2	?	0	1	?	0	0	?	?	?	0	0	0	0

Taxa/ Characters	577	578	579	580	581	582	583	584	585	586	587	588	589	590	591	592
Sanajeh indicus	?	?	?	?	?	?	?	?	?	?	?	?	?	?	?	?
Najash rionegrina	?	?	?	?	?	?	?	?	?	?	?	?	?	?	?	?
Nanowana godhelpi	?	?	?	?	?	?	?	?	?	?	?	?	?	?	?	?
Alamitophis tingamarra	?	?	?	?	?	?	?	?	?	?	?	?	?	?	?	?
Alamitophis elongatus	?	?	?	?	?	?	?	?	?	?	?	?	?	?	?	?
Nidophis	?	?	?	?	?	?	?	?	?	?	?	?	?	?	?	?
Patagoniophis	?	?	?	?	?	?	?	?	?	?	?	?	?	?	?	?
Nanowana schrenki	?	?	?	?	?	?	?	?	?	?	?	?	?	?	?	?
Powellophis	?	?	?	?	?	?	?	?	?	?	?	?	?	?	?	?
Vasuki indicus	?	?	?	?	?	?	?	?	?	?	?	?	?	?	?	?
Gigantophis garstini	?	?	?	?	?	?	?	?	?	?	?	?	?	?	?	?
Madtsoia pisdurensis	?	?	?	?	?	?	?	?	?	?	?	?	?	?	?	?
Gigantophis sp	?	?	?	?	?	?	?	?	?	?	?	?	?	?	?	?
Madtsoia camposi	?	?	?	?	?	?	?	?	?	?	?	?	?	?	?	?
Adinophis	?	?	?	?	?	?	?	?	?	?	?	?	?	?	?	?
Platyspondylophis	?	?	?	?	?	?	?	?	?	?	?	?	?	?	?	?
Menarana nosymena	?	?	?	?	?	?	?	?	?	?	?	?	?	?	?	?
Menarana laurasiae	?	?	?	?	?	?	?	?	?	?	?	?	?	?	?	?
Madtsoia madagascariensis	?	?	?	?	?	?	?	?	?	?	?	?	?	?	?	?
Madtsoia bai	?	?	?	?	?	?	?	?	?	?	?	?	?	?	?	?
Yurlunggur camfieldensis	?	?	?	?	?	?	?	?	?	?	?	?	?	?	?	?
Wonambi naracoortensis	?	?	?	?	?	?	?	?	?	?	?	?	?	?	?	?

Supplementary Dataset 2: continued

**Taxa/ Characters**	**593**	**594**	**595**	**596**	**597**	**598**	**599**	**600**	**601**	**602**	**603**	**604**	**605**	**606**	**607**	**608**
Sanajeh indicus	?	0	0	0	0	0	?	?	?	?	?	?	0	1	0	?
Najash rionegrina	0	0	0	1	0	0	0	?	?	0	?	?	0	1	0	1
Nanowana godhelpi	?	?	?	?	?	?	?	?	?	?	?	?	0	1	0	1
Alamitophis tingamarra	?	?	?	?	?	?	?	?	?	?	?	1	?	1	0	1
Alamitophis elongatus	?	?	?	?	?	?	?	?	?	?	?	1	?	1	0	1
Nidophis	?	?	?	?	?	?	?	?	?	?	?	1	?	1	0	1
Patagoniophis	?	?	?	?	?	?	?	?	?	?	?	?	0	1	0	1
Nanowana schrenki	?	?	?	?	?	?	?	?	?	?	?	?	?	1	0	?
Powellophis	?	?	?	?	?	?	?	?	?	?	?	?	0	1	0	1
Vasuki indicus	?	?	?	?	?	?	?	?	?	?	?	?	0	1	0	1
Gigantophis garstini	?	?	?	?	?	?	?	?	?	?	?	?	0	1	0	1
Madtsoia pisdurensis	?	?	?	?	?	?	?	?	?	?	?	?	?	1	0	1
Gigantophis sp	?	?	?	?	?	?	?	?	?	?	?	?	?	1	0	1
Madtsoia camposi	?	?	?	?	?	?	?	?	?	?	?	1	0	1	0	1
Adinophis	?	?	?	?	?	?	?	?	?	?	?	?	?	1	0	1
Platyspondylophis	?	?	?	?	?	?	?	?	?	?	?	?	0	1	0	1
Menarana nosymena	?	?	?	?	?	?	?	?	?	?	?	?	?	1	0	1
Menarana laurasiae	?	?	?	?	?	?	?	?	?	?	?	?	?	1	0	?
Madtsoia madagascariensis	?	?	?	?	?	?	?	?	?	?	?	1	0	1	0	1
Madtsoia bai	?	?	?	?	?	?	?	?	?	?	?	?	0	1	0	?
Yurlunggur camfieldensis	1	1	1	?	0	0	?	0	1	?	?	1	0	1	0	1
Wonambi naracoortensis	1	1	1	?	0	0	?	?	1	?	0	1	0	1	0	?

**Taxa/ Characters**	**609**	**610**	**611**	**612**	**613**	**614**	**615**	**616**	**617**	**618**	**619**	**620**	**621**	**622**	**623**	**624**
Sanajeh indicus	?	?	?	?	0	0	1	1	?	?	?	0	0	1	?	?
Najash rionegrina	1	?	1	0	0	0	1	1	1	0	0	0	0	1	?	0
Nanowana godhelpi	1	1	1	?	0	0	1	1	1	?	?	0	0	?	?	1
Alamitophis tingamarra	1	1	1	?	0	0	1	1	?	?	?	0	0	?	?	?
Alamitophis elongatus	1	1	1	?	0	1	1	1	?	?	?	0	0	?	?	?
Nidophis	1	1	1	?	0	0	1	1	?	?	?	0	0	?	?	?
Patagoniophis	1	1	1	?	0	0	1	1	?	?	?	0	0	?	?	1
Nanowana schrenki	?	?	1	?	1	0	1	1	?	?	?	0	0	?	?	?
Powellophis	1	1	1	?	0	0	1	1	?	?	?	1	0	1	?	1
Vasuki indicus	1	1	1	?	0	1	0	1	?	?	?	0	0	?	?	0
Gigantophis garstini	0	1	1	?	0	1	0	1	?	?	?	0	0	?	?	0
Madtsoia pisdurensis	0	1	1	?	0	1	0	1	?	?	?	1	0	?	?	?
Gigantophis sp	?	1	1	?	?	?	1	1	?	?	?	0	0	?	?	?
Madtsoia camposi	1	1	1	?	0	1	1	1	1	?	?	0	0	?	?	1
Adinophis	?	1	1	?	0	1	1	1	?	?	?	0	?	?	?	?
Platyspondylophis	1	?	1	?	0	1	1	1	?	?	?	0	?	?	?	1
Menarana nosymena	1	1	1	?	0	0	1	1	?	?	?	?	0	?	?	?
Menarana laurasiae	?	1	1	?	0	1	1	1	?	?	?	0	0	?	?	?
Madtsoia madagascariensis	1	1	1	?	0	1	1	1	1	?	?	0	0	?	?	1
Madtsoia bai	1	1	1	?	0	1	1	1	?	?	?	0	0	1	?	0
Yurlunggur camfieldensis	1	1	1	0	0	1	1	1	?	?	?	0	0	1	0	1
Wonambi naracoortensis	1	1	1	?	0	1	1	1	1	?	?	0	0	1	?	1

**Taxa/ Characters**	**625**	**626**	**627**	**628**	**629**	**630**	**631**	**632**	**633**	**634**	**635**	**636**	**637**	**638**	**639**	**640**
Sanajeh indicus	1	1	?	1	0	2	?	?	?	?	?	?	1	?	?	?
Najash rionegrina	1	1	1	1	0	0	0	0	1	2	1	?	1	1	1	1
Nanowana godhelpi	1	1	1	1	0	1	1	1	1	1	0	?	1	1	1	1
Alamitophis tingamarra	1	1	1	1	0	2	0	1	1	1	0	?	1	1	2	1
Alamitophis elongatus	1	1	1	1	0	2	1	1	1	1	0	?	1	1	2	0
Nidophis	1	1	1	1	0	2	0	1	1	1	0	?	1	1	2	1
Patagoniophis	1	1	1	1	0	0	0	1	1	1	0	?	1	1	1	1
Nanowana schrenki	1	1	1	1	0	1	0	0	1	1	0	?	?	1	1	1
Powellophis	1	1	1	1	1	0	0	0	1	1	0	?	0	1	1	2
Vasuki indicus	1	1	1	1	1	0	0	0	0	1	0	?	1	1	1	2
Gigantophis garstini	1	1	1	1	1	1	0	1	0	1	0	?	1	1	1	2
Madtsoia pisdurensis	1	1	1	1	1	1	0	0	0	1	0	?	1	1	1	2
Gigantophis sp	?	1	1	?	?	0	0	?	?	1	0	?	?	?	1	2
Madtsoia camposi	0	1	1	1	1	2	0	0	0	1	0	?	?	1	1	2
Adinophis	0	1	1	1	1	?	1	0	0	1	0	?	1	1	1	2
Platyspondylophis	1	1	1	1	1	1	1	0	0	1	0	?	?	?	?	2
Menarana nosymena	1	1	1	1	1	0	1	0	0	1	0	?	?	0	2	2
Menarana laurasiae	1	1	1	1	1	0	1	0	0	1	0	?	0	0	1	2
Madtsoia madagascariensis	1	1	1	1	1	0	0	0	0	1	0	?	1	1	1	2
Madtsoia bai	1	1	1	1	1	0	1	0	0	1	0	?	?	0	2	2
Yurlunggur camfieldensis	1	1	1	1	1	1	0	0	0	1	0	0	1	0	1	2
Wonambi naracoortensis	1	1	1	1	1	1	1	0	0	1	0	?	1	0	1	2

Supplementary Dataset 2: continued

Taxa/ Characters	641	642	643	644	645	646	647	648	649	650	651	652	653	654	655	656
Sanajeh indicus	?	?	1	?	?	?	?	?	0	?	?	?	?	?	1	1
Najash rionegrina	1	1	1	1	0	2	1	0	0	1	0	1	0	?	0	1
Nanowana godhelpi	1	1	0	1	1	1	1	1	0	1	0	0	0	?	0	?
Alamitophis tingamarra	0	1	1	2	1	0	1	?	0	1	0	0	0	?	0	1
Alamitophis elongatus	0	1	0	0	0	1	1	1	1	1	0	0	0	?	0	?
Nidophis	0	1	0	2	0	2	1	1	1	1	0	0	0	?	0	1
Patagoniophis	0	1	0	2	0	1	1	1	1	1	1	0	0	?	0	1
Nanowana schrenki	0	1	0	2	0	1	0	1	1	?	1	0	0	?	0	?
Powellophis	1	0	0	1	1	0	1	1	0	1	0	0	0	?	1	1
Vasuki indicus	1	1	0	1	1	2	1	1	0	1	1	1	1	3	1	?
Gigantophis garstini	1	0	1	1	0	2	1	1	0	0	1	0	1	1	1	?
Madtsoia pisdurensis	1	1	1	1	1	0	1	0	0	?	0	1	1	2	1	?
Gigantophis sp	1	1	0	1	1	0	0	1	0	0	?	?	0	?	1	?
Madtsoia camposi	1	0	0	1	1	0	0	1	0	1	1	0	1	1	?	1
Adinophis	0	1	0	?	?	1	0	2	0	1	2	0	0	?	0	?
Platyspondylophis	1	1	0	1	1	2	0	1	0	0	2	0	0	?	0	?
Menarana nosymena	0	0	0	0	1	2	0	1	0	1	1	0	0	?	1	1
Menarana laurasiae	1	0	0	1	0	0	0	1	0	0	?	0	0	?	1	?
Madtsoia madagascariensis	1	0	2	1	1	2	0	0	0	0	1	1	1	0	1	1
Madtsoia bai	1	0	2	0	1	0	0	0	0	0	?	0	1	0	?	1
Yurlunggur camfieldensis	0	0	2	0	1	2	1	2	0	1	0	0	1	0	0	1
Wonambi naracoortensis	0	0	1	0	1	1	0	0	0	0	0	1	1	0	0	1
