# Supplementary material for: Largest known madtsoiid snake from warm Eocene period of India suggests intercontinental Gondwana dispersal
Source: Sci Rep. 2024 Apr 18;14:8054. doi: 10.1038/s41598-024-58377-0 (PMC11549349; doi:10.1038/s41598-024-58377-0)
Supplement: Supplementary file 5 — Supplementary Information 5. [file 41598_2024_58377_MOESM5_ESM.pdf]

Supplementary Information for

Largest known madtsoiid snake from warm Eocene period of India suggests intercontinental Gondwana dispersal

Debajit Datta[1,*] & Sunil Bajpai[1,*]

[1]Department of Earth Sciences, Indian Institute of Technology, Roorkee, Uttarakhand 247667, India; debajitdatta.pd@es.iitr.ac.in; debajitdatta9@gmail.com; sunil.bajpai@es.iitr.ac.in

ORCID

Sunil Bajpai: https://orcid.org/0000-0002-2279-445X

Debajit Datta: https://orcid.org/0000-0001-6078-9830

* Authors for correspondence

Supplementary Information

1. Supplementary Note 1: Geological setting of the Kutch Basin
2. Supplementary Note 2: List of characters used in the phylogenetic analysis 1
3. Supplementary Note 3: List of characters used in the phylogenetic analysis 2
4. Supplementary Table 1: List of specimens for the holotype of *Vasuki indicus*
5. Supplementary Table 2: Measurements of skeletal specimens of *Vasuki indicus*
6. Supplementary Table 3: Measurements of extant boine snakes from Head et al.[15] used in body length estimations.
7. Supplementary Table 4: Measurements of extant boine snakes from Head et al.[15] used in body length estimations.
8. Supplementary Table 5: Measurements of extant snakes from McCartney et al.[16] used in body length estimations.
9. Supplementary figures
10. Supplementary references

1. Supplementary Note 1: Geological Setting

The Kutch Basin, situated along the western continental margin of India (Fig. 1), is a pericratonic rift basin formed during the separation of India from Gondwana during the Jurassic[1–3]. It is bordered by the Precambrian inliers of Nagar Parker in the north, the Saurashtra uplift in the south, the Arabian Sea to the west and Radhanpur-Barmer Arch in the east[4]. The marine Tertiary sequences preserved in the basin is among the most well-preserved in the Indian subcontinent[1,5]. These sequences are exposed as crescentic belts in the south-western coastal plains of the mainland, along the periphery of the Mesozoic highs. The Paleogene succession of Kutch includes the Matanomadh, Naredi, Harudi, Fulra limestone and Maniyara Fort formations, in succeeding order[1]. The study was conducted in the Panandhro area of western Kutch which preserves the lignite bearing succession of the basin (Fig. 1). The lignite deposits overlie the Deccan volcanics and are assigned to the lower-middle members of the early Eocene Naredi Formation[1,5]. A back swamp marshy environment has been envisaged for the lignite deposits at Panandhro and vertebrate fossils including archaic whales (*Andrewsiphius*, *Kutchicetus*), rays and sharks (*Myliobatis*, *Galeocerdo*), bony fish including the catfish *Arius*, and crocodilians and snakes (*Pterosphenius*, madtsoiidae/boidae) have been reported from horizons stratigraphically above the lignites[6–9]. The fossil material described in the present work has also been recovered from a unit above the top lignite seam.

A late Palaeocene–early Eocene age was assigned to the Panandhro lignites by Saraswati and Banerjee[10], whereas later studies based mainly on foraminifers considered a middle Eocene age (Lutetian–early Bartonian) for the lignite succession and a Bartonian age for the overlying vertebrate-bearing unit[11,12]. However, more recently, Agrawal et al.[5] in a detailed study based on palynomorphs and dinoflagellate cysts considered the lignite succession to be of Ypresian age derived from characteristic early Eocene palynomorphs including *Acanthotricolpites* spp., *Neocouperipollis* spp. *Tripilaorites triangulus*, and

Meliapollis. The post-lignitic vertebrate-yielding unit was dated as early Lutetian based on age diagnostic dinoflagellate cysts such as *Homotryblium tasmaniense* and *Homotryblium floripes* (Fig. 1).

2. Supplementary Note 2: Characters used in the phylogenetic analysis 1. Following is the list of characters taken from Zaher et al.[13].

1. Premaxilla fused into single element: absent, paired premaxillae (0); present, fused premaxillae (1).

2. Premaxilla, palatal shelf (also called “vomerine processes”) shape: not bifid posteriorly (0); bifid posteriorly (1).

3. Premaxilla, maxillary process size: prominent (0); reduced (1); absent (2).

4. Premaxilla, maxillary process length relative to level of palatine-maxilla suture: premaxilla medial to contact (0); premaxilla lateral to contact (1).

5. Premaxilla, maxillary process shape: broad distally, with squared-off margin in dorsal view (0); tapers to point distally (1).

6. Premaxillary-maxillary fenestra: absent (0); present (1).

7. Premaxilla body, anterior ethmoidal foramina number: two (0); four or more (1).

8. Premaxilla body, anterior ethmoidal foramina exit via: external naris (0); premaxillary notch (1); premaxillary body (2); between premaxilla and maxilla (3) in maxilla (4).

9. Premaxilla body, ventral ethmoidal foramen (= ventral palatal foramen): present (0); absent (1).

10. Premaxilla body, ventral ethmoidal foramen (= ventral palatal foramen) size: small (0); large (1).

11. Premaxilla-maxilla suture, degree of contact: firm (0); loose (1).

12. Premaxilla, position relative to maxilla: maxilla converges to lateral edge of premaxilla in continuous line, even if not in contact, reaching same level anteriorly as body of premaxilla (0); maxilla tips converge well posterior to premaxilla body (tip of the maxilla displaced posteriorly and not exactly aligned with or separated by a large gap from the premaxilla) (1).

13. Premaxilla internasal process, length: less than half nasal length (0); more than half nasal length (1); extending nearly to, or articulates with, frontal (2).

14. Premaxilla internasal process, shape in cross section: subtriangular (0); compressed (1); depressed (2).

15. Premaxilla internasal process, position relative to nasal descending lamina: at same level as nasals (0); clasped between nasals (1); narrowly clasped (2); lamina abuts base of premaxilla (3); loss lamina-premaxilla contact (4).

16. Premaxilla internasal process, shape in anterior view: tapers or parallel sided (0); widens across nares (1).

17. Premaxilla internasal process, size: well developed (0); very reduced or absent (1).

18. Premaxilla internasal process, shape in lateral view: single (0); bifid, with dorsal ramus joined by a deeper ventral ramus (premaxillary keel of Lee & Scanlon, 2002) extending posteriorly off base of internasal process (1).

19. Premaxilla, maxillary process with conspicuous vertical margin: absent (0); present (1).

20. Premaxilla, alveolar margin, shape in rostral view: horizontal in anterior view (0); arched for passage of tongue during protrusion with mouth closed (i.e., lingual arch), at least below base of internasal ramus as ethmoidal nerve enters (1).

21. Nasals fused: absent (paired) (0); present (1).

22. Nasals, anterior width relative to nasofrontal joint width: exceeds (0); subequal (1).

23. Nasal-prefrontal suture: present (0); absent (1).

24. Nasal-maxilla suture: present (0); absent (1).

25. Nasal, descending lamina: absent (0); present, extending below level of nasal-maxilla suture (1).

26. Nasal supranarial process in dorsal view, development**:** prominent (0); reduced or absent (1).

27. Nasal-maxilla suture, anterior portion configuration in cross section: maxilla overlaps nasal (0); nasal partly overlaps maxilla (1); nasal abuts maxilla (2); nasal underlaps maxilla to floor of narial chamber (3).

28. Nasals, extent of ventral contact beneath premaxillary internasal process: broad (0); narrowly at apex only (if at all) (1).

29. Nasals, dorsal contact over premaxilla internasal process: absent (0); at apex only (1); broadly in contact dorsally (2).

30. Nasals, size: well developed (0); reduced to narrowly elliptic elements plastered to either side of premaxilla internasal process (1).

31. Nasal, shape: not small and cruciform (0); small and cruciform (1).

32. Nasal, length relative to frontal length: nasal shorter (0); nasal longer (1).

33. Nasal, anterior extent: extends anterior to maxillary tooth row or vomer (0); terminates posterior to end of maxillary tooth row or vomer tip (1).

34. Naso-frontal fontanelle: absent (0); present (1).

35. Naso-frontal suture, shape: without V-shaped nasal process into frontal midline (0); with prominent V-shaped nasal process into frontal midline (1).

36. Nasal-frontal, dorsal articulation extent: present, broad anteroposteriorly (0); present, narrow anteroposteriorly (1).

37. Naso-frontal joint: absent, descending laminae of the nasal insert conformably between frontal pillars (0); present, nasal with discrete ventral pegs that pivot on base of frontal pillars (1).

38. Nasal descending laminae, frontal pillar articulation extent: contact over at least dorsal half of frontal pillar (0); ventrally only (1); dorsally only (2); broadly separated (3).

39. Nasal descending lamina, shape at ventral contact with frontal: abutting (0); reduced to point contact (1).

40. Nasal, configuration of medial articulation to frontal on skull roof: nasal laps over frontal near midline (0); frontal laps over nasal near midline (1); no overlap (0).

41. Nasal, lateral articulation to frontal: nasal beveled ventrally over lateral frontal articulation (0); nasal not beveled laterally (1); nasal broadly separated from frontal laterally (2); nasal beveled dorsally at frontal overlap (3).

42. Nasal, supranarial excavation development: weak (0); prominent (1).

43. Frontals, degree of fusion: paired (0); fused (1).

44. Frontal-maxilla suture: absent, frontal separated from maxilla by nasal-prefrontal contact (0); present, frontal contacts maxilla, separating nasal from prefrontal (1).

45. Frontal subolfactory processes, development: absent (0); arch beneath brain but do not contact (1); arch beneath brain to articulate on ventral midline (2); arch beneath brain and fuse on ventral midline (3).

46. Frontal subolfactory process, depth from skull roof to palatine: 25–35% (0); 42–53% (1); 58–68% (3); >89% (4).

47. Frontal subolfactory process-parasphenoid body suture: absent (0); present (1).

48. Frontal subolfactory process, descending lamina (= medial frontal pillars): absent (0); present (1).

49. Frontal subolfactory process, descending laminae: does not clasp parasphenoid rostrum dorsal margin (0); clasps parasphenoid rostrum dorsal margin (1).

50. Frontal subolfactory process descending lamina, position relative to one another: broadly separated (0); narrowly separated (0).

51. Frontal subolfactory processes delimit deep narrow canal across most of orbit: absent (0); present (1).

52. Frontal subolfactory process prefrontal lamina, development: absent (0); knob-like process at anteromedial rim of subolfactory process with prefrontal facet (1); conspicuous

descending lamina off subolfactory process articulating just behind prefrontal (2); produced into shelf supporting prefrontal ventrally (3).

53. Frontal subolfactory process, anterior end shape: unmodified (0); forms thickened anterolaterally projecting flange (1).

54. Medial frontal pillar, relationship to subolfactory process: separated by gap (0); sutured (1); fused (2).

55. Frontal, medial flange separating olfactory tracts, orientation: vertically positioned (0); slants forward (anterior margin of subolfactory process in front of anterior margin of frontal dorsal lamina) (1); slants backwards (anterior margin of subolfactory process behind anterior margin of frontal dorsal lamina) (2).

56. Frontal descending process-parietal contact, configuration in horizontal section: no contact (0); parietal overlaps frontal laterally (1); frontal descending process abuts parietal (2); frontal descending process overlaps parietal, at least in part (3).

57. Frontal interorbital width/frontoparietal suture width I: 14–19% (0); 20–22% (1); 24–26% (2); 28–34% (3); 36–40% (4).

58. Frontal interorbital width/frontoparietal suture width II: <44% (0); 44–47% (1); 50–53% (2); 55–58% (3); 60–63% (4).

59. Frontal, degree of dorsal overlap with prefrontal: narrow (0); broad (1).

60. Frontal, supraorbital shelf: absent (0); present (1); demarcated medially by narrow shallow longitudinal furrow often bearing row of foramina (2).

61. Frontal, anterior margin shape: mainly trends anteromedially (0); broadly transverse (1).

62. Frontal, blunt process on lateral surface of subolfactory process: absent (0); present, extending into prefrontal socket (1).

63. Frontal and parietal, articular margin shape: flat (0); frontal convex and parietal concave in sagittal section (1).

64. Frontoparietal suture, degree of fusion: separate (0); fused (1).

65. Frontoparietal suture, degree of interdigitation: none, frontal overlaps parietal dorsally (0); lightly interdigitating or simple abutment (1); moderate interdigitation (2).

66. Frontoparietal suture, dorsal outline shape: bowed anteriorly or inverted U (0); roughly transverse (1); shallow U or W bowed posteriorly (2); deeply bowed posteriorly U or W (3); frontal postero -dorsolateral corner protrudes posterolaterally (4).

67. Frontoparietal suture, lateral overlap: frontal extensively overlaps parietal (0); frontal barely overlaps parietal (1); frontal underlaps parietal (2).

68. Frontoparietal fontanelle in adult: absent (0); present (1).

69. Suture between frontal and parietal in medial wall of orbit, inclination: strongly anteriorly inclined (1); vertical or slightly anteriorly inclined (2).

70. Frontal suboptic shelf: absent (0); present (1).

71. Frontal suboptic shelf, degree of parietal contact below optic foramen: close to or in contact (1); widely separated (1).

72. Frontal suboptic shelf, depth below optic foramen: shallow, extending just beyond top of sphenoid rostrum (0); deep, more than halfway to ventral margin of sphenoid rostrum (1).

73. Postfrontal: present (as separate element throughout ontogeny) (0); absent (1); fused to postorbital (2); fused to frontal (3).

74. Postfrontal parietal process length relative to frontal process: triradiate (notched distally or not), with subequal frontal and parietal processes wrapping around frontoparietal suture (0); parietal process much shorter than frontal process (1); parietal process absent, postfrontal subtriangular (2).

75. Postfrontal, distal shape: tapering to point (passing anterior to postorbital) (0); bifid (clasps postorbital) (1).

76. Postfrontal, nature of overlap with parietal table: ventrolateral (0); dorsal overlap present (1); dorsal overlap extensive (2).

77. Postfrontal and jugal, relative positions: widely separated (0); nearly in contact (1); in contact (2).

78. Postfrontal supratemporal shelf, development: absent (0); present as thin shelf extending over anterodorsal corner of supratemporal fenestra (1); extending posteriorly further than laterally across upper temporal fenestra (2); occludes upper temporal fenestra (3).

79. Postfrontal enlarged and flat: not (0); often very broad, always antero-posteriorly extensive and flat, with postorbital (1); with a shaft that is club-shaped distally (2).

80. Postorbital: present (0); absent (1).

81. Postorbital, discrete tab underlaping postfrontal anteriorly near jugal articulation: absent (0); present (1).

82. Postorbital, shape: widens anteriorly (0); narrows anteriorly (1).

83. Postorbital and parietal, degree of contact: postorbital entirely distal, separated by postfrontal from parietal (0); partly posterior to postfrontal (1); to contact parietal ventrolaterally at frontoparietal suture (2).

84. Postorbitofrontal, orbital rim development: deep (0); shallow (1).

85. Postorbital, small compressed tab at apex passing across frontoparietal suture: absent (0); present (1).

86. Postorbital, degree of occlusion of upper temporal fenestra (UTF): none, postorbital tapers to a tip (0); partly occludes UTF, as postorbital expands medially posteriorly (1); enlarged postorbital nearly completely occludes UTF (2).

87. Postorbital, exclusion of squamosal contribution to upper temporal fenestra: absent (0); present (1).

88. Postorbital and squamosal, degree of overlap: postorbital overlaps laterally in V-shaped recess in squamosal (0); postorbital overlaps dorsomedially as slender, tapering rod attached superficially (1); postorbital overlaps dorsally (2).

89. Postorbital-squamosal suture, tightness of contact: firm, suture no wider than those among surrounding elements (0); loose, sutural gap wider than that between postorbital and postfrontal or postorbital and jugal (1).

90. Postorbital, degree of contact with skull roofing bones (postfrontal or parietal): substantial contact firmly sutured to skull roofing bones (0); postorbital barely underlaps parietal at frontoparietal suture (1); postorbital tapers to blunt tip that does not contact parietal (2).

91. Postorbital-ectopterygoid contact: absent (0); present (1).

92. Postorbital, jugal ramus length: extends deep to quadrate head (0); extends to level of quadrate head (1); extends to or above quadrate head (2).

93. Postorbital-jugal suture: long, firm, immobile suture, with jugal largely ventrolateral to postorbital (0); shorter suture, with jugal reduced to tab-like dorsal tip that still lies distal to postorbital (1); jugal tapers smoothly to apex, which is loosely joined to lateral face of postorbital (2).

94. Postorbital spreads onto dorsal surface of postfrontal: absent (0); present (1).

95. Postorbital, posterior extent: to end of parietal table (or less) (0); posterior to parietal table (1).

96. Parietals, degree of fusion: paired (0); fused (1).

97. Parietal ventral lappet, development: poorly developed or absent (1); developed into a prominent V-shaped, flat process (1).

98. Parietal, location of origin of temporal musculature: dorsally on parietal table and supratemporal process of parietal (0); ventrally on parietal table and dorsally on supratemporal process (1); ventrally on parietal table and supratemporal process (2).

99. Parietal, extent of temporal musculature (evidenced by shape of temporal fossa): temporal muscles originate dorsally across entire parietal table, all the way to the frontal anteriorly (at least laterally) (0); anterolateral corner of temporal fossa terminates posteriorly; dorsal and ventral margins of temporal fossa converge behind frontal, so parietal table extends as flat surface toward orbital margin, and temporal muscles are confined laterally (1).

100. Parietal sagittal crest, development: absent (0); present (1); projecting dorsally (2).

101. Parietal, nuchal fossa width: narrow (0); wide (1); overgrown by parietal (nearly) to midline (1).

102. Parietal, postparietal projection near midline (bifid distally or not): absent (0); present (1).

103. Parietal and supraoccipital, nature of contact: absent (0); parietal overlaps supraoccipital on midline (1); abuts supraoccipital on midline (2); dorso-ventral parasagittal abutment (3).

104. Parietal bifid supraoccipital process: absent (0); present (1).

105. Parietal descending lamina, articulation with supraoccipital ascending lamina: absent (0); present (1).

106. Parietal, extent of braincase coverage in dorsal view: does not cover occiput (0); covers nearly all of occiput (1); with emarginate lateral fossae (2).

107. Parietal posterior margin, shape (dorsal view): does not form an elongate, slender and pointed posterior process (0); does form an elongate, slender and pointed posterior process (1).

108. Parietal, distinct ventrolateral ridge that extends posterior to prootic and forms a prominent, ventrally concave, shelf-like crest: absent (0); present (1).

109. Parietal supratemporal process, length: well-developed (0); reduced, less than 25% of parietal width (1); absent (2).

110. Parietal supratemporal process, orientation: directed laterally (0); directed posterolaterally (1); directed posteriorly (2).

111. Parietal, contribution to back of the upper temporal fenestra: short supratemporal process, parietal only forms about half of the upper temporal fenestra posterior arch, with supratemporal forming distal half (0); long parietal supratemporal process extends distally to near the quadrate head (1).

112. Parietal foramen: present (0); absent (1).

113. Parietal foramen, position: within parietal (0); at frontoparietal suture (1); within frontal (2).

114. Parietal supraorbital process: absent (0); present (1); deeply clasping frontal orbital margin (2).

115. Parietal postorbital process: absent; parietal barely, if at all, laps behind postorbital apex in horizontal section (0); present; parietal has a vertically-oriented lappet extends laterally to overlap postorbital to form anteromedial margin of upper temporal fenestra (1).

116. Parietal epipterygoid process: absent (0); present as a distinct process (1); present and reaches alar process of prootic (2).

117. Parietal and prootic, nature of contact: absent (0); contact at apex of alar process (1); extensive conformable contact with parietal overlapping prootic laterally throughout length (2); discrete ventral process of parietal overlaps prootic alar process laterally (3).

118. Parietal enclosing brain laterally: uninflated (0); inflated (1).

119. Medial parietal pillar, degree of separation of telencephalon and mesencephalon: absent (0); present, with separation at about mid-height of auditory chamber (1); present, with pronounced ridge separating the telencephalon and mesencephalon (2).

120. Parietal ventral triangular downgrowths of temporal muscle origin overlaps prootic laterally, with latter abutting former medially, just anterior to supraoccipital: absent (0); present (1).

121. Maxilla (post-) premaxillary process contact: absent (0); present, or nearly so, but always excluding premaxilla from vomer dorsally (1); present and vertically expanded (2).

122. Maxilla premaxillary process, shape of dorsal surface: not grooved or enclosed (0); grooved or enclosed for passage of a deeper and more internally placed ramus of the subnarial artery (1).

123. Maxilla and vomer, contact at anterior margin of fenestra exochoanalis: absent (0); present (1).

124. Maxilla facial process, length as a percent of maxilla length: 10–15% (0); 16–23% (1); 25–36% (2); 38–55% (3); >56% (4).

125. Maxilla, facial process height: tall, extending to skull roof (0); reduced (1); absent (2); columnar process received in longitudinal concavity on anterior face of prefrontal (3).

126. Maxilla, facial process apical surface orientation: lateral (0); dorsolateral (1); anterodorsal (2); dorsal, sharply set off from nearly vertical external surface of facial process (3); medial, roofing (more or less) external naris from canthal crest (4).

127. Maxilla, shape of medial face of facial process: smooth (0); posterodorsally trending ridge demarcating the anterior limits of a shallow oval fossa bordered by the lacrimal and infraorbital canals posteriorly (1).

128. Maxilla, supradental shelf development: present, with acute medial margin throughout length (0); reduced anterior to palatine process (1).

129. Maxilla, orientation of narial margin: rises at high angle (0); rises at low angle (1).

130. Maxilla, bears discrete narial margin on facial process bordering external naris: present (0); absent (1).

131. Maxilla, prominent medially projecting palatine process: absent (0); present (1).

132. Maxilla and palatine, articulation: firmly sutured or interlocked (0); separated by fibrous gap (1); loosely ligamentous connection via finger-like lateral maxillary process of palatine and medially projecting palatine process of maxilla (2); maxilla free of palatine, suspended from prefrontal (2).

133. Maxilla pivots on prefrontal to erect fang: absent (0); present (1).

134. Medial (palatine) process of the maxilla, base lies below the orbit: absent (lies in front of the orbit) (0); base less than half exposed (lies mostly below the prefrontal) (1); base more than half exposed (lies mostly below the orbit) (1).

135. Maxilla-palatine joint angle in ventral view: less than 45 degrees (0); more than 45 degrees posterior margin of maxilla palatine process (1); palatine process of maxilla produced into shelf forming incomplete bony secondary palate (2); tongue and groove maxillary-palatine joint (3).

136. Maxilla, accessory foramen posterior to palatine process: absent (0); present (1).

137. Maxilla, superior alveolar foramen position: positioned near middle of palatine process and opening posterodorsally (0); positioned near anterior margin of palatine process and opening medially or posteromedially (1).

138. Maxilla, nature of superior alveolar foramen: enclosed in canal that opens over base of palatine process on supradental shelf of maxilla (0); runs in open channel on dorsal surface of maxillary supradental shelf before becoming enclosed in maxilla (1); two smaller foramina, one above base of palatine process, and another foramen well anterior to that (2).

139. Maxilla, position of superior alveolar foramen relative to anterior base of palatine process: dorsal (0); ventral (1).

140. Maxilla, superior alveolar foramen position relative to ophidian palatine process: superior alveolar foramen partly enclosed in maxilla palatine process (0); entirely anterior to maxilla palatine process (1).

141. Maxilla, suborbital ramus posterior extent: to roughly mid-orbit (or anterior) (0); to posterior quarter of orbit (1); to posterior edge of orbit (2); posterior to orbit (or frontoparietal suture) (3).

142. Maxilla, suborbital process shape below ectopterygoid: tapers posteriorly (0); widens below articulation (ectopterygoid flange present) (1).

143. Maxilla-jugal, depth below orbit: shallow (0); deep (1).

144. Maxilla, suborbital process tip shape at jugal articulation: suborbital margin slopes smoothly to tip (0); with distinct step or V-shaped notch distally at jugal articulation (1).

145. Maxilla, posterior process extension: to midorbit or longer (0); to anterior half of orbit (1).

146. Maxilla, prominent fossa dorsally above fangs: absent (0); present (1).

147. Intramaxillary joint: absent (0); present (1).

148. Prefrontal, degree of reduction: not reduced (0); reduced (1); absent (2).

149. Prefrontal broadly overlaps frontal posterodorsally: absent (0); present (1).

150. Prefrontal orbitonasal margin, orientation in x-section: slopes ventrolaterally (0); vertical (1); slopes ventromedially (2); extends beneath subolfactory processes (3); extends to near contact with its opposite on midline (4).

151. Prefrontal, posterior extent along orbital margin: terminates in anterior half of orbit (0); extends to mid-orbit (1); extends posterior to mid-orbit (2).

152. Prefrontal boss, development: absent (0); present (1); projecting as canthal crest (2).

153. Prefrontal nasolacrimal cornu: absent (0); present (1).

154. Prefrontal, position of articulation with the frontal: prefrontal articulates with the frontal mainly on its lateral surface (0); prefrontal articulates at anterolateral corner of frontal (mainly on its preorbital ridge) (1); with process extending medially across frontal anterior margin between nasal and frontal (2).

155. Prefrontal, medial extent onto frontal anterior width: extends only 50% or less across (0); extends 65% or less across (1); extends 75% or less across (2); extends 85% or more across; approaches midline (3).

156. Prefrontal, anteromedial accessory nasal process: absent (0); present (1).

157. Prefrontal-frontal suture in x-section: prefrontal arcs gently about anterolateral frontal margin along entire anteroposterior length (0); prefrontal strongly bifid, clasps frontal posteriorly then spreads dorsally and reduced ventrally anteriorly (1); prefrontal fits across frontal width anteriorly into V-shaped notch above prefrontal flange (2).

158. Prefrontal, length relative to height: long anteroposteriorly (0); short anteroposteriorly (1).

159. Prefrontal, anterior margin deeply emarginated, with concave anterior margin in lateral view: absent (0); present (1).

160. Prefrontal-maxilla articulation: prefrontal narrowly (or not at all) in contact with maxilla lateral to palatine (0); prefrontal broadly contacts maxilla supradental shelf lateral to palatine (1); prefrontal has mobile contact with maxilla (2); rod-like prefrontal arched dorsally, bifid at each end, with mobile joints at (3).

161. Prefrontal, shape of mobile articulation with maxilla: without distal notch (0); distal notch fits onto maxillary tab (1).

162. Prefrontal, ventral margin of lateral surface: articulates along its entire length with the dorsal surface of the maxilla (0); retains only posterior contact with dorsal surface of maxilla (1).

163. Prefrontal arcs about orbitonasal fenestra, with posteroventromedial corner curving inwards toward palatine in x-section: absent (0); present (1).

164. Prefrontal, supramaxillary shelf development: absent (0); present, roofs maxillary fossa (1); present, projects lateral to frontal articulation (2).

165. Lacrimal: present (0); absent (1).

166. Lacrimal, position relative to lacrimal duct: lacrimal with broad exposure laterally, reaching from lateral floor of lacrimal duct up the medial face of the maxilla to contact a lateral process of the prefrontal that roofs the lacrimal duct in cross-section (0); lacrimal arches over the lacrimal duct to replace the prefrontal dorsally, broadly floors the lacrimal duct with a medial process posteriorly passing up the lateral face of the prefrontal (1); lacrimal reduced to floor of lacrimal duct and lingual surface of maxilla, and barely, if at all, exposed laterally (2); lacrimal bone reduced ventrally, confined mainly to dorsolateral corner of lacrimal duct (3).

167. Lacrimal foramen, size: not large (0); large (1).

168. Lacrimal foramen, number: (0); two, divided on orbital surface (1); two, divided through to olfactory surface (2).

169. Lacrimal duct position: between prefrontal and lacrimal (0); enclosed in prefrontal, except ventrally (1); enclosed entirely in prefrontal (2).

170. Jugal: present (0); absent (1).

171. Postfrontal, contribution to posterior orbital margin: < 38% (0); 39-52% (1); 53-66% (2); 67-80% (3); >80% (4).

172. Jugal, shape at skull roof contact: jugal tapers to tip, confined to parietal, sometimes just touching frontal (0); jugal with long process off apex that broadly overlaps the frontal (1).

173. Jugal, anterior extent with respect to tooth row: jugal broadly overlaps level of posterior maxillary tooth row (0); jugal does not reach anterior to level of the last maxillary tooth (1); jugal fails to reach most posterior maxillary tooth (2).

174. Postfrontal, dorsomedial head shape: undivided (0); Y-shaped (divided into two heads) (1); T-shaped (expanded anteroposteriorly) (2).

175. Jugal-frontal contact: on parietal only (0); contacts frontal (1).

176. Jugal, anterior extent relative to the prefrontal: broadly separated from prefrontal (0); reaches level of prefrontal (1).

177. Jugal-lacrimal, nature of overlap: jugal lateral to lacrimal (0); jugal medial to lacrimal (1); jugal ventral to lacrimal (2).

178. Jugal-maxilla articulation, shape in cross section: rounded ventral margin of jugal and shallow and more rounded contour of the maxilla supradental shelf (0); acute ventral margin of jugal lies in narrow longitudinal groove on dorsal surface of maxillary supradental shelf (1).

179. Jugal, lateral extent over maxilla in cross section: maxilla suborbital border wraps dorsally around jugal external margin (0); jugal laps over external suborbital margin (1).

180. Jugal with inverted v-shaped notch clasping suborbital edge of maxilla: absent (0); present (1).

181. Jugal, lateral exposure below orbit: absent (0); partly exposed above orbital margin of maxilla (1); entirely exposed above orbital margin of maxilla (2).

182. Jugal, suborbital ramus height: shallow (0); deep (1).

183. Jugal suborbital boss: absent (0); present (1).

184. Jugal, postorbital ramus development: complete bony postorbital bar (0); incomplete bony postorbital bar (1); bony postorbital bar absent (2).

185. Jugal, postorbital ramus shape in lateral outline: narrow (0); wide (1).

186. Jugal-squamosal contact: present (0); absent (1).

187. Jugal, posterior process development: complete lower temporal bar (0); reduced to a discrete bony posterior process (1); lost entirely (2).

188. Jugal, posterior process orientation: more posterior in orientation (0); more ventral in orientation (1).

189. Jugal, medial ridge development: medial ridge weak, jugal lateral to ectopterygoid at base in dorsal view (0); medial ridge pronounced, base of medial ridge projects behind ectopterygoid base in dorsal view (1).

190. Jugal, shape in cross section at level of ectopterygoid: subtriangular (0); depressed (1).

191. Jugal-parietal contact: absent (0); present (1).

192. Jugal, suborbital process: present (0); absent (1).

193. Jugal-maxilla suture, development: firmly sutured (0); weakly sutured via ligamentous connection (1).

194. Jugal, position of articulation relative to parietal lateral wings: dorsal (0); ventral (1); both, jugal clasps lateral wing (2).

195. Jugal-parietal overlap: present (0); absent, jugal entirely lateral to parietal (1).

196. Squamosal: present (0); absent (1).

197. Squamosal supratemporal ramus, length relative to epipterygoid position: does not extend anterior to level of epipterygoid (0); extends anterior to level of epipterygoid (1).

198. Squamosal and parietal, degree of contact via squamosal temporal ramus: temporal ramus diverges from parietal supratemporal process (0); temporal ramus broadly contacts parietal supratemporal process (1).

199. Squamosal and parietal, degree of contact via base of squamosal temporal ramus: diverges from parietal (0); base lies against parietal (1).

200. Squamosal, temporal ramus width: slender (0); widens anteriorly with medial shelf along parietal that roofs posterior end of upper temporal fenestra (1).

201. Squamosal, temporal ramus shape: compressed (0); depressed (1).

202. Squamosal, ascending process development: fully developed (0); reduced to nub (1); absent (2).

203. Squamosal, distinct transverse facet for quadrate head: present (0); absent, abutting at tip only (1).

204. Supratemporal: present (0); absent (1).

205. Supratemporal shortens: supratemporal longer than squamosal-parietal contact (0); supratemporal shorter than squamosal-parietal contact (1); supratemporal very small (2).

206. Supratemporal lengthens: stops short of level of parietal notch (0); subequal in anterior extent with level of parietal notch (1); anterior to level of parietal notch (2).

207. Supratemporal anterior suture with parietal shape: supratemporal lies flat against supratemporal process of parietal (0); enters slot in supratemporal process of parietal (1).

208. Supratemporal position on parietal: partly ventral (0); partly ventrolateral (1); all lateral (2); dorsal (on either parietal or braincase alone) (3).

209. Supratemporal, nature of articulation to skull: firm osseous connection (0); loose fibrous connection (1).

210. Supratemporal, position of anterior terminus (Tchernov et al., 2000): posterior to level of trigeminal nerve exit (0); anterior to level of trigeminal nerve exit (1).

211. Supratemporal, degree of overlap onto parietal table (only in snakes without supratemporal process of the parietal): supratemporal does not extend anterior of posterior border of parietal table (0); supratemporal extends anteriorly to overlap parietal table (not scored in snakes with reduced or absent supratemporal) (1).

212. Supratemporal, orientation from quadrate head: anterior to quadrate head (0); dorsal to quadrate head (1).

213. Supratemporal, posterior extension: supratemporal ends near attachment to braincase (0); supratemporal extends freely posterior to otooccipital (1).

214. Supratemporal, exposure in dorsal view: partly exposed dorsally on lateral side of parietal supratemporal process (0); hidden completely from view by parietal-squamosal contact dorsally (1).

215. Supratemporal, posterior exposure on parietal supratemporal process: narrow or absent (0); present broadly (1).

216. Quadratojugal: present (0); absent (1).

217. Quadrate head, nature of attachment to skull: tapering peg-like head loosely attached in socket formed largely by squamosal (0); quadrate head pivots on slender tapering tip of squamosal (1); bluntly abuts supratemporal and squamosal (2).

218. Quadrate head, suspension: separated from braincase by supratemporal and squamosal (except narrowly beneath tip of supratemporal) (0); quadrate head abuts braincase ventral to supratemporal (1); quadrate head broadly contacts braincase anteriorly (2).

219. Quadrate suprastapedial process: absent (0); present (1).

220. Quadrate, shape: slender (0); broad with a rectangular shape (1).

221. Quadrate lateral conch: present (0); absent (1).

222. Quadrate and pterygoid, degree of overlap: extensive (0); short overlap or small lappet (1); very narrow overlap or lappet absent (2); no overlap, ligamentous connection only (3).

223. Quadrate, accessory process arising off anteromedial edge near quadrate head: absent or poorly developed (0); well-developed, abuts braincase in region of horizontal semicircular canal (1).

224. Quadrate stylohyal process on medial face of quadrate, development: absent (0); present as oval disc (1); present as narrow cylindrical ridge (2).

225. Quadrate, height as a percentage of braincase depth (measured near the quadrate head): less than 50% (0); 50–59% (1); 60–69% (2); 70–74% (3); more than 79% (4).

226. Quadrate, degree of bowing (i.e., “pythonomorph”): bowed slightly, but not in both lateral and posterior views (0); strongly bowed anteriorly in lateral view and laterally in posterior view, throughout length; with prominent ventrally directed suprastapedial process forming cavum tympani (1).

227. Quadrate foramen, size: large (0); small (1); tiny (2).

228. Quadrate slopes anteroventrally (more than 90˚ = anterior slope from quadrate head): vertical to posterior slope (0); 94–107 degrees (1); 108–121 degrees (2); 122–135 degrees (3); greater than or equal to 136 degrees (4).

229. Quadrate slopes posteroventrally (less than 90˚ = posterior slope from quadrate head): vertical to anterior slope (87-93 degrees) (0); 86–68 degrees (1); 67–49 degrees (2); 48–31 degrees (3); less than 30 degrees (4).

230. Quadrate, proximal head with prominent anterior process: absent (0); present (1).

231. Quadrate, lateral crest off head boarders ventral fossa: absent (0); present (1).

232. Quadrate, rod-like shape: absent (0); present, rod-like (1); present, rod-like with triangular dorsal process distally (2).

233. Stapes, foramen for stapedial artery: absent (stapes imperforated) (0); present (stapes perforated) (1).

234. Stapes, shaft length: longer than diameter of stapedial footplate (0); equal to or shorter than stapedial footplate (1).

235. Stapes, shape of shaft at base: straight (0); bent (1).

236. Stapedial footplate, size: small (0); large (1).

237. Stapedial footplate, size relative to fenestra ovalis: does not fill fenestra ovalis (0); fills fenestra ovalis (1).

238. Stapedial footplate orientation at base of shaft: lateral edge of footplate well anterior to medial (0); lateral edge of footplate nearly in same cross-sectional plane as medial edge (1).

239. Fenestra ovalis, orientation: opens directly laterally (0); opens anterolaterally (1); opens ventrolaterally (2); opens posterolaterally (dorsolaterally) (3).

240. Extracolumella: present (0); absent (1).

241. Septomaxilla: present (0); absent (1).

242. Septomaxilla dorsolateral contacts: no contacts (0); abuts laterally with prefrontal and nasal (1); abuts laterally with nasal only (2); abuts laterally with prefrontal only (3).

243. Septomaxilla, anterior end: meets maxilla in immovable joint (0); mobile, septomaxilla not contacting maxilla (1).

244. Septomaxilla, position relative to vomeronasal organ: occupies a lateral position, not contributing to nasal cavity or to roofing of vomeronasal organ (0); occupies a more medial position, contributing to nasal cavity and roofing vomeronasal organ (1).

245. Septomaxilla, dorsal expansion: flat or weakly convex, vomeronasal organ small (0); expanded and convex, reflecting large size of vomeronasal organ (1).

246. Septomaxilla, dorsal exposure: septomaxilla covered by nasal or only slightly exposed dorsally lateral to nasal margin (0); septomaxilla broadly exposed dorsally below nasal (1).

247. Septomaxilla, contact with dorsal surface of palatal shelf of maxilla: absent (0); present (1).

248. Septomaxilla divides vomeronasal organ: absent (0); present (1).

249. Septomaxilla, development of long posterodorsally-directed, blade-like process (medial flange) nearly to frontal: absent (0); present, but nasal intercedes between septomaxilla and

frontal (1); extends to frontal beneath nasal (2); develops an expanded faceted articulation with the frontal (3).

250. Septomaxilla, development of lateral flange: absent (0); present (1); reaches well above roof of vomeronasal organ (2).

251. Septomaxilla lateral flange apex widely separated from lateral margin of nasal in dorsal view: absent (0); present (1).

252. Septomaxilla lateral flange, position relative to vomeronasal opening: posterior base of lateral flange located at or posterior to palatal opening of vomeronasal organ (0); posterior base of lateral flange distinctly anterior to vomeronasal organ (1).

253. Septomaxilla medial flange: absent (0); present (1).

254. Septomaxilla lateral flange base length relative to lateral margin length: broad-based, more than one third lateral margin (0); narrow-based anteriorly (1); narrow-based posteriorly (2).

255. Septomaxilla, anterolateral tip shape: tapers smoothly to tip (0); with conspicuous hook-like process (1).

256. Septomaxilla, length of median flange along skull midline: short, not reaching level of prefrontal (0); long, extends posteriorly to anteroposterior level of anterior margin of prefrontal (1).

257. Septomaxilla, length of posterior process on laterally ascending flange: short or absent (0); long, extends posteriorly deep to prefrontals (1).

258. Nervus ethmoidalis medialis, position: above septomaxilla (0); enclosed in septomaxilla anteriorly (1); in anterior half of septomaxilla (2); enclosed posteriorly in septomaxilla (3).

259. Vomeronasal organ, development of fungiform body: simple diverticulum of nasal capsule (0); completely separated from nasal capsule, with fungiform body (1).

260. Vomerine concha that supports the mushroom body: unossified except at base (0); ossified fully (1).

261. Vomerine concha mushroom body ossification degree: weak (0); prominent (1).

262. Vomerine concha, extent of ossification posteriorly relative to vomeronasal opening: terminates two-thirds or less of length of vomeronasal opening (0); reaches to level of posterior one-quarter or more of vomeronasal opening (1).

263. Vomer lateral wall of capsule anterior extent relative to vomerine concha: lateral capsular wall terminates posterior to level of vomerine concha (0); lateral capsular wall extends anterior to level of base of vomerine concha (1).

264. Vomer, anteromedial extent of vomeronasal organ fossa relative to vomeronasal opening: to medial apex of vomeronasal notch (0); anterior to medial apex of vomeronasal notch (1); to anteriormost margin of vomeronasal opening (2).

265. Vomer, premaxillary process tip shape: short projection (0); tipped with finger-like process (1); approaches end of septomaxilla (2).

266. Vomer capsule ventral margin extends further laterally: absent (0); present (1).

267. Cupola for vomeronasal organ (vomer-septomaxilla medial contact): fenestrated medially, even if only narrowly (0); closed medially (1).

268. Vomeronasal organ and mushroom body, enclosure: not fully enclosed by septomaxilla and vomer only (0); fully enclosed by septomaxilla and vomer only (1).

269. Vomer, degree of fusion: paired (0); partly fused (1); fully fused (2).

270. Vomer, length: vomer short, caudal end extends posteriorly no further than anteriormost contact of palatine with maxilla (0); vomer long, caudal end extends posteriorly beyond anteriormost contact of palatine with maxilla (or anterior base of palatine process of maxilla when the two do not articulate closely) (1).

271. Vomer, shape: main portion plate-like (0); main portion rod-like (1).

272. Vomer (when looking at skull in ventral view) overlaps (dorsally) the palatal shelf of the maxilla behind posterior margin of opening of vomeronasal organ: absent (0); present (1).

273. Vomer and maxilla palatal shelf, nature of contact: does not establish any sutural contact with the palatal shelf of the maxilla behind the incisura Jacobsoni (0); establishes narrow contact with the palatal shelf of the maxilla behind the incisura Jacobsoni (1); establishes broad overlap with the palatal shelf of the maxilla (2); vomer-maxilla contact formed mainly by tab off palatal shelf of maxilla (3).

274. Vomer, position relative to vomeronasal organ: vomer ventral to vomeronasal organ (0); encapsulates vomeronasal organ posteriorly and medially (1); with margins of enclosing posterior wall sloping ventrolaterally (2); further expanded laterally to completely encapsulate vomeronasal organ posteriorly (3).

275. Vomer meets septomaxilla: at posterior margin of opening of vomeronasal organ (0); at lateral margin of opening of vomeronasal organ (1).

276. Vomer, shape of margin at opening of vomeronasal organ: flat (0); curled downwards (1).

277. Vomeronasal nerve exit, position: dorsal to vomer (0); via canals dorsally on vomer (1); via foramen at back end of vomer (2); via sieve-like arrangement of foramina through back of vomer (3).

278. Vomer, degree underlap of palatine: just at tips (0); extending posteriorly to level of maxilla-ectopterygoid first contact (1).

279. Vomer, ventral longitudinal ridges: absent (0); present, long longitudinal ridges converging toward midline, well-developed below vomeronasal nerve exit from septomaxilla (1); present, short parasagittal ridges anteriorly on vomer at level of vomeronasal duct opening (2).

280. Vomer septum (vertical lamina), transverse fenestration: absent (0); present near mid-line of the vomer septum (1); present at posteroventral corner of vomer septum (2).

281. Vomer, septum (vertical lamina) height: low, not forming septum (0); partly separating olfactory chambers (1); nearly completely separating olfactory chambers along with septomaxilla and nasal (2); only ventral edge of septum remains (3); V-shaped notch separates dorsal and ventral rami of vomer septum (4).

282. Vomer, expanded hollow flange on posterodorsal margin: absent (0); present (1).

283. Vomer, dorsal trough: absent (0); present (1).

284. Vomer, transverse flange rises vertically to meet septomaxilla and encloses vomeronasal organ posteriorly: absent (0); present (1).

285. Vomer, contact with subolfactory process of frontal: absent (0); present (1).

286. Vomer, descending tubercle (or ridge) on vomer at vomero-palatine junction: absent (0); present (1); ridge tubercle present on vomer and or adjacent palatine (2).

287. Vomer, foramina near midline of palatal surface: paired (0); single (1).

288. Vomerine teeth: present (0); absent (1).

289. Palatine, degree of contact on midline: separated (0); anterior contact only (1); contact extends to midpoint, or beyond (2).

290. Palatine, position relative to maxilla-lacrimal-jugal articulation: palatine sits medial to lacrimal and or jugal and maxilla in cross section (0); palatine inserts between lacrimal and or jugal and maxilla in cross section (1).

291. Palatine, dorsal canal development: shallow longitudinal sulcus (0); upturned lateral and medial edges of palatine demarcate deep narrow canal ending in enclosed fossa (1).

292. Palatine, vomerine process dorsally on vomer: vomer attaches over entire face of palatine vomerine process (0); narrow slender tip of palatine loosely attached to vomer (1); long slender palatine process clasped in groove on dorsal surface of vomer (2); ventral edge

of palatine vomerine (choanal) process attached ligamentously between bifid vomer palatine processes (3).

293. Palatine, shape of vomerine process buttressing vomer: palatine vomerine process tapers anteromedially (0); splays laterally at tips to buttress vomer posteriorly (1).

294. Palatine, vomerine process passes vomer: medial to vomer tines (0); lateral to vomer tines (posteromedial process of vomer) (1).

295. Palatine, ventral projections from anterior end of palatine, near palatine-vomer suture: absent (0); present (1).

296. Palatine, ventromedial extension from maxillary process of palatine (choanal process of palatine): present but not descending ventromedially (0); present but descending medioventrally to reach in between (or close to) posterior tips of vomers (1).

297. Palatine maxillary process, position with respect to posterior end of palatine: anterior to posterior end of palatine (last tooth position posterior to main axis process) (0); at posterior end of palatine (last tooth position anterior to main axis process) (1).

298. Palatine-maxilla articulation, extent: broad (0); narrow suture at anterolateral margin (1).

299. Palatine, orientation with respect to pterygoid: lies in same plane (0); palatine diverges strongly anterodorsally (1).

300. Palatine, maxillary process-prefrontal contact above level of maxillary tooth row: absent (0); present (1).

301. Palatine, contribution to suborbital fenestra: reduced posteromedially; pterygoid broadly exposed in suborbital fenestra (0); palatine extends posteriorly along lateral edge of pterygoid so that pterygoid narrowly enters suborbital fenestra (1); palatine fully excludes pterygoid from border of suborbital fenestra (2).

302. Palatine-pterygoid, overlap: palatine overlaps pterygoid at tip and ectopterygoid near base, otherwise lateral in position (0); palatine overlaps pterygoid dorsally from lateral to near

medial margin of pterygoid, with loose abutment laterally (1); palatine barely overlaps pterygoid laterally and pterygoid does not extend well anterior to ectopterygoid-jugal-maxilla juncture (2); palatine barely overlaps pterygoid, joint nearly horizontal (3); complex pattern of clasping projections (4).

303. Palatine, slot dorsally that accommodates vertical flange from pterygoid: absent (0); present (1).

304. Palatine, medial overlap with pterygoid: short (0); long (1).

305. Palatine, anterior "dentigerous" process: absent (0); present only as short extension of palatine anterior to maxillary process (1); present (with teeth) (2).

306. Palatine dentigerous process (or edentulous process anterior to maxillary and choanal processes), ventral overlap with the vomer and/or septomaxilla posterolateral to the opening of Jacobson's organ: present (0); absent (palatine dentigerous process does not ventrally overlap the vomeronasal capsule) (1).

307. Palatine, dentigerous process length: long, bearing six or more teeth (0); short, bearing five or fewer teeth (1).

308. Infraorbital canal, anterior division in palatine: absent, single foramen anteriorly (0); present, double foramina anteriorly, with a small medial palatine ramus and a large lateral ramus (1).

309. Infraorbital canal, position: lateral, between palatine and dorsal surface of supradental shelf of maxilla (0); medial, entirely in palatine (1).

310. Palatine foramen: absent (0); present, enters palatine dorsally toward its anterior end to pass anteroventrolaterally into the infraorbital canal (1).

311. Palatine, choanal process: curves medially and meets the vomer in a well-defined articular facet (0); touches or abuts the vomer without articulation, or remains separated from vomer (1).

312. Palatine, choanal process: forms an extensive concave surface dorsal to the ductus nasopharyngeus (0); narrows to form a curved finger-like process (1); forms a short vertical or horizontal lamina that does not reach the vomer (2).

313. Palatine, condition (sensu Rieppel et al., 2008): simplicipalatinate (0); incipient duplicipalatinate (1); intermediate (2); fully duplicipalatinate (3).

314. Palatine, choanal fossa development: absent (0); present anteriorly on palatine (1); extending about half way back on palatine (2); fully developed to end of element (3).

315. Palatine, subchoanal process medial edge shape in ventral view: present only on anterior one-third of palatine (0); roughly arcuate (1); roughly parasagittal (2).

316. Palatine, extent of posterior emargination of anterodorsal margin of choanal fossa: anterior to anteroposterior midpoint of palatine-maxilla suture (0); extends posterior to anteroposterior midpoint of palatine-maxilla suture (1).

317. Palatine, posteromedial process length: long, overlaps at least two pterygoid teeth (0); short, overlaps no more than one pterygoid tooth (1); absent (2).

318. Palatine, discrete planar surface extends along ventrolateral margin between pterygoid and maxillary sutures lateral to choanal fossa: absent (0); present (1).

319. Palatine teeth: present (0); absent (1).

320. Palatine teeth, size: small, conical denticles (0); enlarged, but smaller than marginal teeth (1); highly enlarged, similar in size to marginal teeth (2).

321. Pterygoid, sutural contact on midline: present (0); absent (palatal rami fully separated) (1).

322. Pterygoid, separation on midline: pterygoids narrowly separated for most of their length (0); broad separation at base, narrow anteriorly (1); broad separation at base, but not as narrowly separated anteriorly (2); broad separation throughout length (3).

323. Pterygoid, palatine ramus: contacts vomer (0); does not contact vomer (1).

324. Pterygoid-palatine joint, length of complex pattern of projections (Tchernov et al., 2000): long (0); medium (1); short (2).

325. Pterygoid, palatine ramus clasps pterygoid ramus of palatine: absent (0); present (1).

326. Pterygoid transverse process and ectopterygoid (pterygoideus muscle insertion) nearly as deep as mandible (at least 80% mandible depth), and ectopterygoid transversely broad, covering most of transverse process of pterygoid in anterior view: absent (0); present (1).

327. Pterygoid, posterior extent: pterygoid does not reach level of occipital condyle (0); pterygoid reaches level of occipital condyle (1); pterygoid reaches well posterior to level of occipital condyle (2).

328. Pterygoid, transverse process development: distinct, well-defined lateral projection (0); gently curved lateral expansion or absent (1).

329. Pterygoid, quadrate ramus short and small, tightly wrapping around posteromedial (ventromedial if quadrate horizontally oriented) surface of quadrate: absent (0); present (1).

330. Pterygoid, quadrate ramus shape (Tchernov et al., 2000): robust, rounded or triangular in cross section, but without groove (0); blade-like, with distinct longitudinal groove for the insertion of the protractor pterygoidei muscle (1).

331. Pterygoid, ventral flange (wing-shaped extension) of quadrate ramus: absent (0); present (1).

332. Pterygoid quadrate ramus, longitudinal ventral fossa: absent (0); ventral longitudinal fossa present and set off by ventral ridges at lateral and medial margins (1).

333. Pterygoid quadrate ramus, lateral edge curves dorsally: absent (0); present (1).

334. Pterygoid quadrate ramus, dorsolateral longitudinal ridge: absent (0); present (1).

335. Pterygoid quadrate ramus dorsal longitudinal fossa orientation: faces dorsomedially (0); faces medially, C-shaped (1).

336. Pterygoid quadrate ramus notch near posteroventromedially: absent (0); present as distinct step along margin (1); present and deeply emarginate (2).

337. Pterygoid teeth: present (0); absent (1).

338. Pterygoid teeth, development: small conical denticles (0); enlarged, but smaller than marginal teeth (1); highly enlarged, similar in size to marginal teeth (2).

339. Pterygoid teeth, regional extent: restricted to palatal ramus of pterygoid (0); extend posteriorly onto quadrate ramus of pterygoid (1).

340. Ectopterygoid: present (0); absent (1).

341. Ectopterygoid, size and restriction of suborbital fenestra: ectopterygoid relatively slender, fenestra widely open (0); ectopterygoid enlarged medially, restricting suborbital fenestra (1); ectopterygoid highly enlarged medially, closing suborbital fenestra (2).

342. Ectopterygoid, angulation in dorsal view: nearly orthogonal rami (0); obtuse angle (1).

343. Ectopterygoid, anterior length: well separated from palatine above maxilla (0); near to or in contact with palatine (1).

344. Ectopterygoid, with slot laterally for maxilla articulation: absent (0); present (1).

345. Ectopterygoid, degree of overlap anteriorly across dorsal surface of maxilla: narrow overlap dorsally (0); broad but short dorsal overlap (1); extensive dorsal overlap (2).

346. Ectopterygoid-maxilla suture, nature of contact: ectopterygoid lies dorsally, at least in part, along supradental shelf of maxilla (0); ectopterygoid abuts posteromedial corner of maxilla (1); ectopterygoid overlapping maxilla more ventrally than dorsally (2); interdigitating suture (3).

347. Ectopterygoid maxillary process, shape in dorsal view: tapers or parallel-sided (0); widens anteriorly at maxilla overlap (1); widens anteriorly to more than three times width of ectopterygoid shaft (2).

348. Ectopterygoid, maxillary process anterior notch: tapers forward of maxilla contact (notch absent in maxillary process) (0); notched anteriorly (1); with large, rectangular, lateral ramus produced directly laterally (2).

349. Ectopterygoid, orientation of lateral edge of maxillary ramus at maxilla margin: slopes medially (0); straight (1).

350. Ectopterygoid-maxilla, posterior process suture: ectopterygoid articulates primarily with maxilla (0); ectopterygoid contacts jugal only (1).

351. Ectopterygoid, prefrontal and palatine relations: ectopterygoid does not underlap palatine posteriorly below prefrontal (0); ectopterygoid underlaps palatine below prefrontal (1).

352. Ectopterygoid-palatine, ventral articulation: palatine-maxilla contact excludes ectopterygoid (0); ectopterygoid anterior process largely separates palatine from maxilla posteriorly (1).

353. Ectopterygoid hooked posterior process flat and exposed dorsally, ventrally and laterally: absent (0); present (1).

354. Ectopterygoid, posterior process, development: prominent (0); small lateral knob (1); absent (2).

355. Ectopterygoid, posterior process length: does not extend past coronoid apex (0); extends past coronoid apex (1).

356. Ectopterygoid, dorsal process height: tall (0); short (1); absent (2).

357. Ectopterygoid, prefrontal contact: does not contact prefrontal (0); contacts prefrontal at base of orbit (1).

358. Ectopterygoid, nature of overlap with pterygoid: ectopterygoid embraces pterygoid anteriorly (0); ectopterygoid overlaps pterygoid dorsally (1); ectopterygoid abuts pterygoid laterally (2); ectopterygoid underlaps pterygoid ventrally (3).

359. Ectopterygoid-pterygoid overlap, position of former relative to latter at overlap: overlap dorsolateral in position, well lateral to pterygoid teeth (0); broad flange spreads onto dorsomedial face of pterygoid, just above tooth row (1); caps pterygoid in prominent inverted V-shaped groove near posterior apex (2).

360. Ectopterygoid distal tip buttressed by medial pterygoid flange: absent (0); present (1); prominent (2).

361. Ectopterygoid, pterygoid process length: short (0); longer, but still anterior to trigeminal foramen (1); longest, extending posterior to trigeminal foramen (2).

362. Ectopterygoid, extent of overlap of pterygoid: short (0); long (1).

363. Epipterygoid: present (0); absent (1).

364. Epipterygoid, resting position: located lateral to prootic (even if only narrowly so) (0); located entirely anterior to prootic (1).

365. Epipterygoid, position relative to alar process of prootic: epipterygoid anterolateral to prootic alar process (0); epipterygoid abuts anteroventral tip of alar process (1).

366. Epipterygoid, length: long, reaches nearly to level of top of braincase, or above quadrate head, or more than half the distance between pterygoid and parietal table (0); short, reaches only to level of quadrate head, barely to semicircular canal, or half or less the distance between pterygoid and parietal table (1).

367. Epipterygoid-parietal, contact: absent (0); present, epipterygoid overlaps parietal temporal muscle origin (1).

368. Epipterygoid, shape: expanded dorsoventrally and ventrally (0); columelliform (1).

369. Braincase, degree of fusion: unfused in adult (0); opisthotic and prootic fused in adult (1); complete braincase fusion in adult (2).

370. Processus ascendens of synotic tectum: absent (0); present (1).

371. Supraoccipital, number of elements in adults: single (fused supraoccipitals) (0); paired (1).

372. Supraoccipital, origin of temporal muscles: restricted to parietal (0); spread onto supraoccipital, contacting nuchal crest in roughly T-shaped outline (1); spread onto supraoccipital to form Y-shaped crest (2); temporal muscles spread onto braincase dorsally, but sagittal and nuchal crests join to form roughly anchor-shaped outline (3).

373. Supraoccipital nuchal crest, lateral extent: absent (0); present on supraoccipital only (1); present on supraoccipital and otoccipital (2).

374. Supraoccipital crest: absent (0); present (1); meets ventral parietal (2).

375. Supraoccipital, position relative to otooccipital on midline: overlaps otooccipital laterally (0); overlaps otooccipital on midline as part of sagittal crest (1); overlaps otoccipital on midline and capped by the parietal; all three bones visible in cross section (2).

376. Supraoccipital, contribution to internal sidewall of neurocranium: participates in sidewall (0); absent, only dorsal plate remains (1); dorsal plate absent (2).

377. Epiotic foramen: absent (0); present (1).

378. Prootic, alar process: small or absent (0); prominent (1).

379. Prootic, supratrigeminal process development: absent (0); weakly developed, not projecting beyond cupola anterior (1); present; finger-like projection above trigeminal notch, projecting beyond cupola anterior (2).

380. Crista prootica (i.e., ridge on lateral surface of the prootic, overhanging facial foramen), development: well-developed lateral flange (0); reduced to weak ridge (1); absent (2).

381. Crista prootica, length relative to basipterygoid process: does not extend onto basipterygoid process (0); extends onto basipterygoid process forming open or closed bony canal (1).

382. Crista prootica, aliform shape in ventral view (“extended butterfly shape”): absent (0); present, aliform outline in ventral view (1); present, prominent, extending further laterally (2).

383. Crista tuberalis and crista prootica, degree of contact: separate (0); combined to surround stapedial footplate and lateral aperture of recessus scalae tympani (1).

384. Juxtastapedial recess defined by crista circumfenestralis: absent (0); present but open posteriorly (1); present and closed posteriorly (2).

385. Prootic, anterior rim of crista circumfenestralis, posterior extent relative to medial margin of stapedial footplate: former well anterior to latter (0); former roughly on same level as latter in dorsal view at level of shaft (1).

386. Crista circumfenestralis, degree of enclosure of stapedial footplate: footplate mostly exposed (0); edges of crista converge upon stapes to conceal most of footplate (1).

387. Crista interfenestralis: prominent (0); reduced (1); absent (2).

388. Crista interfenestralis deeply overlaps stapedial footplate along its lateral margin: absent (0); present (1).

389. Crista tuberalis: prominent (0); reduced (1); absent (2).

390. Facial foramen (i.e., lateral exit on prootic for cranial nerve VII), number of openings: single (0); double (1).

391. Exit foramen for the hyomandibular branch of the facial nerve is located inside the opening for the mandibular branch of the trigeminal nerve: outside via long canal (0); inside via long canal (1); facial nerve exits into trigeminal fossa via very short canal (2).

392. Prootic, participation in medial aperture of the recessus scala tympani (MARST): absent (0); prootic forms part of MARST (1).

393. Posterior auditory foramen, bordering bones: bordered by opisthotic (otooccipital) posteromedially (0); enclosed entirely in prootic (1).

394. Orbitosphenoid, calcified/ossified: absent (0); present (1); expanded to floor the braincase (2).

395. Orbitosphenoid, size: well developed (0); reduced (1).

396. Orbitosphenoid, number of elements in adults: paired (0); single (fused ventrally) (1).

397. Optic foramen: present (0); absent (1).

398. Optic foramen, bone(s) forming margin: not fully enclosed by bone (0); enclosed partly by frontals (1); entirely within orbitosphenoid (2); entirely within parietal (3); entirely within frontal (4).

399. Optic foramen, size: small or medium-sized foramen (0); large fenestra (1).

400. Trigeminal foramen or foramina, bone(s) forming margin: anterior margin not enclosed in bone (0); anterior margin enclosed by descending flange of parietal (1); anterior margin enclosed by orbitosphenoid (2); enclosed by prootic (3).

401. Trigeminal nerve maxillary branch foramen, bone(s) forming margin: pierces the lateral (maxillary) process of the palatine (0); passes dorsally between the palatine and the prefrontal (1).

402. Ophidiosphenoid (equals "laterosphenoid" or "pleurosphenoid") ossification in braincase: absent (0); present (1).

403. Retractor pits in dorsum sellae: present (0); absent, retractor bulbi muscles absent (1).

404. Dorsum sellae, shape in longitudinal cross-section: crista sellaris forms posterior wall, usually low and vertically disposed with more or less anterior slope (0); dorsum sellae poorly differentiated with, at most, shallow fossa with low crista sellaris (1); enclosed in distinct fossa expressed as a cup-like depression walled laterally and ventrally by the basisphenoid and anteriorly by the parasphenoid rostrum (2); completely enclosed tube-like dorsum sellae (3).

405. Dorsum sella, development of fossa roofed posteriorly by crista sellaris (not scored in species with reduced/absent crista sellaris): fossa only modestly roofed by crista sellaris (0); roofing more extensive over deep fossa (1).

406. Sphenoidal keel, development: absent (0); present below dorsum sellae (1); deep keel (2).

407. Sphenoid rostrum, shape of ventrolateral margin at distal tip: tapers to a point (0); distinctly bifid (1).

408. Sphenoid rostrum, prominent ventrolaterally directed alae: absent (0); present (1).

409. Sphenoid rostrum, position of trabeculae relative to midline: trabecular bases wide set (0); close set (1).

410. Sphenoid rostrum, distribution of trabecular grooves: poorly delimited along fronto-parasphenoid contact (0); prominently delimited over most of rostrum (1).

411. Sphenoid rostrum, dorsal margin shape: not arrow-shaped (0); arrowhead-shaped where clasped by suboptic ridges on subolfactory processes (1).

412. Sphenoid rostrum broadly exposed dorsally between frontal subolfactory processes: absent (0); present (1).

413. Sphenoid rostrum dorsal surface flat against base of frontal subolfactory processes between descending laminae: absent (0); present (1).

414. Sphenoid rostrum behind optic foramen: broad (0); narrow (1).

415. Sphenoid-basioccipital suture transversely crested: absent (0); present (1).

416. Lateral wings of the basiphenoid: absent (0); present (1).

417. Crista trabeculares, development on expanded sphenoid rostrum: short and or indistinct (0); elongate and distinct in lateral view (1).

418. Cultriform process (i.e., sphenoid rostrum), length: long (0); short (1); absent (2).

419. Vidian canal rostral opening: roofed by parietal (0); exits via parasphenoid rostrum only (1).

420. Vidian canal, number anterior openings in main ramus: one (0); two (1).

421. Vidian canal, intracranial course anteriorly, running atop lateral margin of rostrum to exit between parietal and sphenoid: absent (0); present (1).

422. Right Vidian canal, posterior size: small (0); large (1).

423. Left Vidian canal, posterior size: small (0); large (1).

424. Trabeculae cranii: tropibasic (0); platybasic (1).

425. Basipterygoid process (and synovial palatobasal articulation): present, formed by ossified basitrabecular process (0); present, formed by outgrowth from parabasisphenoid (no basitrabecular process known; synovial palatobasal articulation absent) (1); basipterygoid process absent (2).

426. Basipterygoid process, length: long, i.e., projecting far beyond the body of the basisphenoid (0); short, i.e., not projecting very far beyond the body of the basisphenoid (1).

427. Basipterygoid process: not expanded at distal end (0); distal end expanded (1).

428. Sesamoid bone at basipterygoid-pterygoid articulation: absent (0); present (1).

429. Vidian canal formed by the basisphenoid enclosing the internal carotid artery, and the base of the palatine artery, as they pass over the basipterygoid process: absent (0); present (1).

430. Vidian canal, location of posterior opening: within basisphenoid (0); anterior margin at basisphenoid-prootic suture (1); entirely within prootic (2); the dibamid-amphisbaenian condition (3).

431. Vidian canal, posterior entrance position relative to lateral margin of sphenoid: laterally placed, near sphenoid lateral edge or further posteriorly (0); medially placed, far from sphenoid lateral edge (1).

432. Carotid artery exits anterior end of Vidian canal: at same level (or slightly above) as the remnant of the embryonic neurocranial trabeculae (0); below the level of the remnant of the embryonic neurocranial trabeculae (1).

433. Parietal margin of the optic foramen: straight (0); concave (i.e. the parietal is notched by the optic foramen) (1).

434. Basal tubera, position: posterolateral, with apex on lateral edge of basioccipital just behind base of prootic-opisthotic suture (0); anteromedial, with apex at lateral juncture of sphenoid and basioccipital, anterior and medial to prootic-opisthotic suture (1).

435. Apophyseal ossification (Element X) caps basal tubera: absent (0); present (1).

436. Occipital condyle: posterior surface of condyle straight in ventral view (0); posterior surface of condyle concave in ventral view (1).

437. Basioccipital: contributes to ventral border of foramen magnum (0); excluded from ventral border of foramen magnum by contact of exoccipitals (1).

438. The basioccipital meets the sphenoid in a suture located at the level of: fenestra ovale (behind facial foramen) (0); facial foramen (1); trigeminal foramen (foramina) (2).

439. Occipital condyle, shape in cross section: not circular, with deep notochordal depression dorsally (0); more circular in section, with shallow notochordal notch dorsally (1); circular, without notch dorsally (2).

440. Basioccipital ventral keel: absent (0); crest (1); keel (2).

441. Medial aperture of the recessus scalae tympani, position: between basioccipital and opisthotic (0); entirely in opisthotic (1).

442. Recessus scalae tympani floor: formed by basioccipital (basioccipital expanded laterally to form the floor of the recessus scalae tympani) (0); formed by otooccipital (basioccipital excluded from the floor of the recessus scalae tympani by the otooccipital (1).

443. Cranial nerve IX exits braincase via: medial aperture of the recessus scala tympani (MARST) internally and lateral aperture of recessus scala tympani (LARST) externally (0); exits dorsal to MARST then out LARST (1).

444. Cranial nerve IX exits braincase via: foramen magnum (0); laterally via LARST (1); posteriorly via vagus (=jugular) foramen (2).

445. Medial aperture of the recessus scalae tympani subdivided, cranial nerve IX exits posteriorly: absent (0); large oval MARST undivided, with cranial nerve IX exiting at posterodorsal end (1); MARST divided into anterior and posterior openings by bony process, with cranial nerve IX exiting via posterodorsal foramen (2).

446. Vagus foramen (jugular foramen in other amniotes) far from medial aperture of the recessus scalae tympani: with hypoglossal foramina lying below and between them medially (0); vagus foramen closer to MARST, with hypoglossal foramina extending posterior to vagus (1).

447. Hypoglossal (XII) foramina exit(s) relative to vagus (X–XI) foramen on external surface of braincase: hypoglossal foramina separated from vagus (0); at least one hypoglossal foramen emerges from the same fossa as the vagus foramen (1); only one hypoglossal foramen still exits separately from the vagus foramen fossa (2); all three hypoglossals emerge from the same fossa as the vagus foramen (3).

448 Vagus (jugular) foramen, concealed in lateral view by crista tuberalis: absent (0); present (1).

449. Lateral aperture of recessus scalae tympani, size: open (0); small (1); closed (2).

450. Perilymphatic foramen faces: ventrally (0); medially (1); laterally (2); posteriorly (3).

451. Opisthotic-exoccipital fusion to form otoccipital: incompletely fused or separate in adult (0); completely fused early in post-hatching ontogeny (1).

452. Otooccipitals (exoccipital part) contact above foramen magnum to exclude supraoccipital: absent (0); present, supraoccipital excluded from border of foramen magnum (1).
453. Otooccipitals, broadly in contact, covering occipital condyle in dorsal view: absent (0); present, covering occipital condyle (1); present, but instead of being transverse, the otooccipital posterior margin slopes anteriorly to re-expose occipital condyle (2).
454. Supratemporal facet on otooccipital is sculptured and delineated with projecting posterior rim that overhangs exoccipital: absent (0); present (1).
455. Otooccipital, posterolateral processes development: short and narrow, do not extend toward posterior margin of occipital condyle (0); wider than condyle and long, combine with crista tuberalis to extend to approximate posterior margin of occipital condyle (1).
456. Metotic fissure: open (0); subdivided by contact of basal plate and otic capsule (1).
457. paroccipital process of otooccipital projection: paroccipital process well developed and laterally projected (0); reduced to a short projection (1); vestigial or absent (2).
458. Mandibular symphysis: present, anterior tips of dentary with distinct flat symphyseal area (0); absent, anterior tips of dentary smoothly rounded and without distinct symphyseal area (1).
459. Dentary, orientation in cross section: more or less vertical (0); more nearly horizontal (1).
460. Mandible, curvature in lateral view: straight to weakly bowed (0); conspicuously bowed (1).
461. Dentary, shape of anterodorsal edge of dental parapet at tip: straight (0); tipped medially (1).
462. Dentary, degree of ventral bowing along long axis: straight to slightly bowed (0); distinctly bowed ventrally (1).

463. Dentary, overlap with postdentary bones laterally: extensive (0); reduced (1).

464. Dentary suspended from: overlapping parts of coronoid, surangular, prearticular, splenial and angular (0); surangular (1); prearticular (2).

465. Dentary, relative length of rami forming kinetic notch: ventral ramus conspicuously shorter than dorsal (by at least 4 tooth positions) (0); with dorsal and ventral rami subequal in length posteriorly (1); ventral ramus conspicuously longer than dorsal ramus (2).

466. Dentary subdental shelf/gutter, development in anterior part of dentary: subdental shelf absent (0); weakly developed subdental shelf (1); pronounced subdental gutter (2).

467. Dentary, depth of lingual margin of subdental surface below gutter: narrow throughout length (0); deep anteriorly, narrow posteriorly (1); deep anteriorly and posteriorly (2).

468. Dentary, number of mental foramina on lateral surface: none (0); one (1); two (2); three (3); four or more (4).

469. Dentary, size of posteriormost mental foramen: same size as others (0); enlarged relative to others (1); smaller relative to others (2).

470. Dentary, mental foramen position: near tip of dentary (0); displaced posteriorly (1); displaced further posteriorly (2). [Modified]

Modifications were introduced to increase the range of scoring of this character.

471. Dentary coronoid process posterior termination: below (or anterior) to level of coronoid apex (0); just behind level of coronoid apex (1); well posterior to level of coronoid apex (2).

472. Dentary subdental shelf hooks around anterior rim of the anterior inferior alveolar foramen: absent (0); present (1).

473. Dentary surangular process, nature of articulation with surangular: lies flat against the dorsolateral face of the surangular below the coronoid (0); set in a posterodorsally trending groove, open dorsally, that supports it from below on the dorsolateral face of the surangular

below the coronoid (1); set in deep V-shaped, laterally-facing recess on dorsolateral face of surangular behind coronoid (2).

474. Dentary coronoid process posterodorsal extension: absent or with only small dorsal extension (0); large, but extending between lateral and medial processes of coronoid (1); large, but extending dorsally to overlap most of anterolateral surface of coronoid (2); extremely well developed, covering almost entire lateral surface of coronoid (3).

475. Dentary angular process reduced: angular process extends to or past coronoid apex (0); anterior to coronoid apex (1); anterior to level of coronoid bone (2).

476. Dentary, position of angular process termination relative to splenio-angular joint: anterior to splenio-angular joint (0); posterior to splenio-angular joint (1); well posterior to splenio-angular joint (2).

477. Dentary, broad, flat angular process lies beneath mandible, nearly horizontally disposed in vivo: absent (0); present (1).

478. Dentary posterior termination on lateral face of mandible: below (or anterior to) level of coronoid apex (0); just posterior to coronoid apex (1); well posterior to level of coronoid apex (2); nearly to posterior surangular foramen (3).

479. Dentary, V-shaped kinetic surangular notch: absent (0); present (1).

480. Dentary, angular process prominently bifid: absent (0); present (1).

481. Dentary, ventromedial flange: absent (0); present (1).

482. Meckel’s canal: opens medially for most of length (0); opens ventrally anterior to anterior inferior alveolar foramen (1).

483. Dentary, degree of restriction of Meckel’s canal: does not restrict or enclose Meckelian canal (0); lower dentary border of Meckel's canal folds up to approach closely upper border to restrict canal (1); upper and lower borders form sutural contact anterior to splenial (2); Meckel's canal closed and fused anterior to splenial (3).

484. Splenial, attachment to dentary above Meckel's canal: close throughout length (0); loose, with dorsal dentary suture confined to posterodorsal corner of splenial (1).

485. Splenial, extent of contact to dentary posterodorsally (posterior to anterior inferior alveolar foramen): broadly articulated posteriorly to anterior inferior alveolar foramen (0); narrowly articulated posteriorly (1); little or no contact posteriorly (2).

486. Splenial: present as separate element (0); fused to prearticular (1); fused to dentary (2); lost (3).

487. Splenial, anterior extent relative to dentary tooth row: about one-third (or less) (0); about one-half (1); about two-thirds (2); three-fourths (or more) (3).

488. Splenial, posterior extent relative to apex of coronoid: extends posteriorly to or beyond apex of coronoid (0); does not extend posteriorly to apex of coronoid (1).

489. Splenial-angular articulation: splenial overlaps angular (0); with ball on splenial (below level of posterior mylohyoid foramen) fitting into socket on angular (1); with ball on angular fitting into socket on splenial (2); flat, abutting joint (3).

490. Splenial anterior inferior alveolar foramen, position relative to dentary: enclosed entirely in splenial (0); between splenial and dentary (1).

491. Splenial, anterior inferior alveolar foramen: present (0); absent (1).

492. Splenial anterior inferior alveolar foramen, position relative to anterior mylohyoid foramen: anterodorsal (0); dorsal to posterodorsal (1).

493. Angular: present (0); absent (1).

494. Angular, posterior extent: reaches mandibular condyle (0); does not reach mandibular condyle (1).

495. Angular length posteriorly: more than half way to mandibular condyle (0); one half or less of distance to mandibular condyle (1); one third or less distance to mandibular condyle (2).

496. Angular taller anteriorly, closely approaching coronoid (or, if coronoid absent, tooth-bearing margin of dentary above Meckelian canal): absent, angular broadly separated from coronoid (0); present (1); with finger-like process over-arching Meckel's canal (2).

497. Angular, medial exposure (relative degree of medial exposure scored with the teeth pointing straight up): broad (0); reduced (1); narrow (2).

498. Posterior mylohyoid foramen, position: absent (0); medial (1); ventral (2); lateral (3).

499. Posterior mylohyoid foramen, position relative to coronoid apex: below (0); posterior (1); anterior (2).

500. Coronoid eminence, development: present (0); reduced (1); absent (2).

501. Coronoid eminence, composition: formed by both surangular and coronoid (0); formed exclusively by coronoid (1); formed exclusively by surangular (2).

502. Coronoid anteromedial process fits into sulcus beneath tooth-bearing border of dentary (at or behind end of tooth row): absent (0); present (1); wraps around ventral margin of dentary tooth-bearing border at apex posteriorly (2).

503. Coronoid bone, development: present, well developed (0); present, small and straplike (1); absent (2).

504. Coronoid-surangular articulation: coronoid restricted to medial aspect of surangular/mandible (0); coronoid extends onto dorsal surface of surangular (1); coronoid arches over dorsal margin of mandible to reach lateral face of surangular (2).

505. Coronoid, anteromedial process: present (0); absent (1).

506. Coronoid, anteromedial ventral margin (at/behind end of tooth row): overlapped by splenial (0); abuts splenial (1); does not contact splenial (2).

507. Coronoid, posteromedial process: absent (0); present (1).

508. Coronoid, anterolateral dentary process development: absent (0); present (1); overlaps dentary tooth row deeply (2).

509. Coronoid, anterolateral dentary process shape: extends anteroventrally and smoothly tapers into dentary (0); extends anteriorly, with dorsal and ventral margins more parallel sided, terminating in a blunt edge anteriorly (1).

510. Surangular, insertion into dentary lateral to the intramandibular septum, entering the intramandibular canal: absent (0); inserts lateral to the intramandibular septum, slightly entering the intramandibular canal (1); inserts lateral to the intramandibular septum, deeply entering the intramandibular canal (2).

511. Surangular-dentary articulation, position of posterior tooth row: relatively narrow posterodorsal tooth-bearing portion of dentary clasps acute dorsal margin of compressed surangular (0); relatively broad posterodorsal tooth-bearing portion of dentary sits atop flat dorsal surface of surangular (1).

512. Surangular, anterior extent in dentary kinetic notch: extends to no more than four tooth positions from end of tooth row (0); extends to at least six tooth positions from end of tooth row (1); extends to at least nine tooth positions from end of dentary (2).

513. Surangular, number of external foramina: two foramina, anterior and posterior (0); single foramen (1).

514. Surangular, anterior surangular foramen size: small (0); large (1).

515. Surangular adductor fossa on external face of mandible: shallow and extends ventrally no more than halfway down (0); deep and extends ventrally more than halfway down (nearly to angular bone) (1).

516. Surangular, adductor fossa posterior extent: large, shallow depression broadly separates deep part of adductor fossa from rim of mandibular cotyle (0); adductor fossa extends posteriorly to near the mandibular cotyle (1).

517. Surangular, distinct crest marks edge of lateral jaw adductor, especially posteriorly: absent (0); present (1).

518. Surangular, orientation of dorsal margin relative to level of tooth crowns: nearly horizontal, set below level and rising somewhat toward the coronoid (0); rises steeply anterodorsally to coronoid, with apex reaching above (1).

519. Prearticular and surangular, fusion in adults: absent (0); present (1).

520. Prearticular and surangular, nature of contact: absent (0); present, broad contact behind posteromedial process of coronoid, restricting mandibular adductor fossa anteriorly (1).

521. Prearticular, extent of margin on border of adductor fossa: medial wall of adductor fossa no higher than lower half of glenoid fossa (0); prearticular wall reaches to at least top of glenoid fossa (1); prearticular ala arched margin rises well above glenoid buttress (2).

522. Prearticular fossa: absent (0); present (1).

523. Prearticular tubercle: absent (0); present (1).

524. Prearticular crest: absent (0); present (1).

525. Mandibular cotyle, shape: relatively flat (0); deep and arched transversely (saddle shaped cotyle) (1).

526. Retroarticular process, length relative to cotyle length: knob-like (0); subequal to mandibular cotyle length (1); longer than mandibular cotyle length (2).

527. Retroarticular process, length relative to cotyle width at midpoint: much longer than mandibular cotyle is wide at midpoint (0); shorter than cotyle width (1); absent (2).

528. Retroarticular process very long: more or less prominent (0); more than twice as long as mandibular cotyle length (1); nearly 10x as long as mandibular cotyle length (2).

529. Retroarticular process, orientation: extends posteriorly (0); inflected medially (1).

530. Retroarticular process, orientation in lateral (or posterior) view: extends posteriorly (0); inflected ventrally (1).

531. Retroarticular process, dorsal edge orientation: horizontal (0); inclined posterodorsally (1).

532. Retroarticular process emarginate distally: absent (0); present (1).

533. Retroarticular process, rotation: both margins of retroarticular process on same plane (0); rotated about long axis, so inner edge is lower than outer edge (1).

534. Retroarticular process, lateral notch forming waist proximally: absent (0); present (1).

535. Retroarticular process breadth (greatest width) relative to mandibular condyle (glenoid): narrower (0); wider (1).

536. Retroarticular process, prearticular pterygoideus process (i.e., angular process of Oelrich, 1956): absent (0); present (1).

537. Retroarticular process, pterygoideus process shape: narrow based (0); crest connects process to tip (1).

538. Premaxillary teeth (apart from median tooth): similar size or larger than anterior maxillary teeth (0); distinctly smaller than anterior maxillary teeth (1).

539. Median premaxillary tooth: absent (0); present (1).

540. Enlarged median tooth on fused premaxilla: median tooth same size as other premaxillary teeth (0); slightly enlarged median premaxillary tooth (1); greatly enlarged median premaxillary tooth (2).

541. Maxillary tooth row, posterior extent: to roughly midorbit (or anterior) (0); to posterior third of orbit (1); posterior to orbit (2).

542. Maxillary tooth, crown height: constant throughout tooth row (0); sinuous occlusal surface (1); convex occlusal surface (2); length decreases posteriorly (3); length increases posteriorly (4).

543. Maxilla, enlarged teeth (fangs) relative to adjacent teeth: absent (0); present on anterior maxilla (1); present on posterior maxilla (2).

544. Dentary teeth, conspicuously enlarged anteriorly: absent (0); present (1); caniniform dentary tooth (one or two) (2).

545. Maxilla tooth row length: to or behind midorbit (0); anterior to midorbit (1); anterior to orbit (2).

546. Premaxillary tooth count: none (0); one to three (1); four to six (2); seven to nine (3); 10 or more (4).

547. Premaxilla teeth: absent (0); present (1).

548. Maxillary tooth count: 0 (0); 2–5 (1); 7–15 (2); 16–27 (3); 31 or more (4).

549. Dentary tooth count: 0 (0); 4–9 (1); 10–20 (2); 21–35 (3); 36 or more (4).

550. Marginal teeth, degree of curvature: all vertical (0); all recurved (1); anterior teeth recurved and posterior teeth vertical (2).

551. Dentary tooth orientation: dorsal (0); dorsolateral (1).

552. Tooth crown, sharp flexure just distal to parapet of jaw: absent (includes simple recurve, teeth relatively short conical and upright) (0); sharp flexure of tooth shaft just above jaw parapet (at least in some teeth) (1).

553. Tooth crowns, with long, slender, needle-like tips: absent (0); present (1).

554. Position of marginal teeth relative to tooth-bearing element: on medial side of tooth-bearing element (when present, subdental shelf narrow) (0); near to or on apical margin of tooth-bearing element (when present, subdental shelf broad) (1).

555. Fusion of marginal teeth: unfused to each other (0); fused to each other (1).

556. Teeth, tightness of attachment: teeth ankylosed to tooth-bearing elements (at tooth maturity) (0); nearly all mature teeth fail to ankylose, held in place by fibrous connective tissue leaving conspicuous gap around tooth bases (1).

557. Bases of marginal teeth infolding: smooth, dentine and enamel not infolded (0); dentine and enamel infolded into pulp cavity, longitudinal grooves externally at base of teeth (1).

558. Bases of marginal teeth expanded: absent (0); present (1).

559. Marginal tooth, spacing: crowns closely spaced (0); crowns separated by large gaps (1).

560. Position of replacement teeth: lingual (0); posterolingual (1).

561. Tooth replacement, number of replacement generations per tooth position: single replacement tooth per tooth position (0); two replacement teeth per tooth position (1).

562. Tooth replacement, number of tooth positions with replacement teeth: no more than half of tooth positions with replacement teeth (0); nearly all tooth positions with replacement teeth (1).

563. Teeth, interdental ridges separate tooth sockets: absent (0); interdental ridges present separating tooth sockets, but socket still open lingually (1).

564. Orientation of replacement teeth: erupt upright, growing straight upwards into functional position (0); erupt horizontally, and then rotate through 90" about the base into functional position (1).

565. Tooth replacement: present (0); absent (1).

566. Resorption pits: present (0); absent (1).

567. Development of resorption pits: at base of teeth (0); on bony tooth pedicel (1).

568. Tooth alveolus shape: wider transversely than anteroposteriorly (0); nearly circular (1).

569. Dentary, tooth-bearing border termination relative to anterior surangular foramen (asf): tooth-bearing border approaches vicinity anterior surangular foramen to variable degree (0); tooth-bearing border broadly separated from anterior surangular foramen by distance roughly equivalent to tooth row length (1).

570. Palatal teeth: constant in size across palatal tooth row (0); decrease in size posteriorly (1).

571. Palatal teeth relative size at palatine-pterygoid transition: palatine teeth larger than adjacent pterygoid teeth (0); adjacent palatine and pterygoid teeth subequal (1); pterygoid teeth larger than adjacent palatine teeth (2).

572. Diastema between palatine and pterygoid tooth rows: absent (0); present (1).

573. Cusps on posterior teeth: unicuspid (0); bicuspid (1); tricuspid (2).

574. Venom groove on anteromedial surface of teeth: absent (0); present, unenclosed (1); present, enclosed tube (2).

575. V-shaped wear facets of maxillary teeth incised on lateral face of dentary: absent (0); present (1).

576. Teeth swollen, set off from tooth shafts above jaw parapet: absent (0); present (1).

577. Basihyal: present (0); absent (1).

578. Basihyal, relationship to skull (when mouth is closed): anterior to braincase (0); ventral to braincase (1); posterior to braincase (2).

579. Hyoid, lingual process length: short (0); medium (1); long (2).

580. Hyoid, distal part of lingual process: not detached (0); detached (1).

581. Free epibranchials (second epibranchial): absent (0); present (1).

582. Free epibranchial: simple (0); complex (has hooks or processes, and or doubles back on itself) (1);

583. First epibranchial: shorter than first ceratobranchial (0); longer than or nearly equal to first ceratobranchial (1).

584. First ceratobranchial (in lateral view): no dorsolateral angulation (0); weak dorsolateral angulation (has a distinct bend) (1); strong dorsolateral angulation (90˚ or more) (2); entire element straight or oriented vertically (3).

585. Second ceratobranchials: present (0); absent (1).

586. Second ceratobranchial: shorter than first ceratobranchial (0); nearly equal to or longer than first ceratobranchial (1).

587. Second ceratobranchial apposed on midline: absent (0); present (1).

588. Large, wing-like hyoid cornu: absent (0); present (1).

589. Hyoid cornu: less than the length of the epihyal (0); greater than or equal to the length of the epihyal (1).

590. Epihyal: meets hyoid cornu at (or near) its distal end (0); meets hyoid cornu along its body (1).

591. Epihyal: expansion or elaboration at proximal end absent (0); simple expansion at proximal end present (1); hook-like elaboration at proximal end present (2); lateral flange at proximal end present (3); medial flange at proximal end present (4).

592. Lateral flange at midpoint of epihyal: absent (0); present (1).

593. Presacral vertebrae number reduction: 24 or more presacrals (0); 23 presacrals (1); fewer than 23 presacrals (2).

594. Presacral vertebrae, number increase I: 24 or fewer (0); 25 (1); 26 (2); 27 (3); 28 or more (4).

595. Presacral vertebrae, number increase II: 32 presacrals or fewer (0); 33 to 39 (1); 50 to 55 (2); 61 to 84 (3); 89 or more (4).

596. Presacral vertebrae, number increase III: less than 104 (0); 118 to 132 (1); 144 to 156 (2); 168 to 180 (3); 184 or more (4).

597. Presacral vertebrae, number increase IV: less than 193 (0); 197 to 209 (1); more than 219 (2).

598. Cervical vertebrae, number reduction: six or more (0); five (1); four (2); three (3); two (4).

599. Cervical vertebrae, number increase: six or fewer (0); seven (1); eight or more (2).

600. Cervical intercentra: present, not fused to the centrum of the vertebrae, suturally connected at most (0); absent (1).

601. Cervical intercentrum, position: intercentral (0); posterior end of preceding centrum (1); anterior end of following centrum (2).

602. Cervical rib ossified portion shape: widens distally, at least in last cervical (0); tapers or does not widen distally (1).

603. Cervical ribs, start on vertebra number: two (0); three (1); four (2); five (3); six (4).

604. Cervical intercentrum length relative to pedicle length: intercentrum longer than pedicle (0); intercentrum shorter than pedicle (1).

605. Cervical pedicle (outgrowth of pleurocentrum to which intercentrum may attach): absent (0); present, projecting ventrally with discrete fore and aft margins (1).

606. Vertebral pedicle (i.e., “hypapophysis”), posterior extent: in anterior half of vertebral column (0); throughout vertebral column (1).

607. Vertebral anterior pedicle, length relative to respective centrum: about 50% of centrum length (0); subequal to or longer than centrum length (1).

608. Vertebral centrum, articulation: amphicoelous (and notochordal) (0); procoelous (1).

609. Zygosphene-zygantrum accessory intervertebral articulations: absent (0); dorsolaterally directed facet continuous with prezygapophyseal articulation located just up edge of neural arch (1); tall, laterally directed facet continuous with prezygapophyseal articulation and extending as high as top as neural canal (2); separate facet set on distinct pedicle and facing ventrolaterally (3).

610. Neural spine on mid-trunk vertebrae: prominent (0); reduced or absent (1).

611. Vertebrae, anterior neural spine shape: anterior trunk vertebral neural spines undifferentiated from those more posteriorly (0); very long, anteroposteriorly narrow, and posterodorsally directed neural spines present (1).

612. Vertebrae, arcual ridges: absent (0); present (1).

613. Vertebrae, centrum shape in ventral view: narrowly subtriangular (0); broadly subtriangular (1).

614. Vertebrae, posterior margin of the neural arch with a deep V-shaped embayment in dorsal view, exposing much of the centrum in front of the condyle: absent (0); present (1).

615. Vertebrae, paracotylar foramina well-developed and consistently distributed throughout vertebral column: absent (0); present (1).

616. Vertebrae, prezygapophyseal accessory processes; absent (0); present (1).

617. Vertebrae, parazygantral foramina: absent (0); present (1).

618. Vertebral synapophyses in relation to lateral edge of prezygapophyses: at the same level or slightly more projected laterally than the lateral edge of the prezygapophyses (0); clearly medial to the edge of the prezygapophyses (1).

619. Vertebral synapophysis shape: undivided more or less dorsoventrally elongate convexity (0); divided, hemispherical oval facet dorsally for primary rib articulation (diapophysis) and flatter inset facet ventrally for rib accessory process articulation (parapophysis) (1).

620. Vertebrae, subcentral paralymphatic fossa: absent (0); present (1).

621. Vertebrae, condyle delimitation: condyle confluent with the centrum ventrally (0); condyle distinctly separated from centrum by a groove at base of centrum and a raised rim about anterior edge of condyle (1).

622. Mid-thoracic vertebrae, pachyostotis: absent (0); present (1).

623. Sacral rib attachment on ilium relative to acetabulum: 1st sacral rib overlaps acetabulum (0); or is entirely posterior to level of acetabulum (1).

624. Sacral vertebrae: present (0); absent (1).

625. Lymphapophyses: absent (0); present (1).

626. Lymphapophyses, articulated lymphapophyses (bifurcated ribs): absent (0); present (1).

627. Caudal autotomic septum, position relative to caudal rib: within caudal rib (0); anterior to caudal rib (1); posterior to caudal rib (2); absent (3).

628. Caudal rib (transverse process) shape: single rib without basal foramen (0); foramen passing through base of rib (1); divergent bifid ribs on some caudals (2).

629. Posterior caudal vertebrae, groove on dorsal surface of neural spines: absent (0); present (1).

630. Caudal vertebrae, pterapophysis: absent (0); present (1).

631. Caudal vertebrae, distal tip of anterior zygapophyses: undifferentiated (0); elaborated into a horizontal blade (1).

632. Caudal haemal arch (intercentrum) position: intercentral, pedicles feeble absent (0); contacting mainly condyle but also distinct pedicles beneath preceding centrum (1); mainly contacting pedicles on preceding centrum but still bordering condyle (2); well forward of condyle on preceding centrum (3).

633. Caudal haemal arch pedicle length: short (0); long (1).

634. Vertebra whose rib first attaches to sternum: seventh (or more anteriorly) (0); eighth (1); ninth (2).

635. Trunk ribs pachyostotic: absent; slender, cancellous ribs (0); present; thick, dense ribs (1).

636. Postcloacal bones: absent (0); present (1).

637. Sternum: present (0); absent (1).

638. Sternal fontanelle: absent (0); present (1).

639. Sternal fontanelle, number: single (0); double (1).

640. Number of rib attachment points to sternum (including attachment of xiphisternum): five (0); four (1); three (2); two or fewer (3).

641. Xiphisternum: present (0); absent (1).

642. Xiphisternal fontanelle: absent (0); present (1).

643. Number of xiphisternal rib attachment points: none (0); one (1); two (2); three (3); four (4).

644. Number of postxiphisternal (or poststernal) inscriptional ribs united along the ventral midline to form continuous chevron shaped structures: 0 (0); 1-4 (1); 5–11 (2); 12–30 (3); >31 (4).

645. Scapulocoracoid: large (0); reduced (1); absent (2).

646. Scapula: short and wide (0); elongate and narrow (1).

647. Suprascapular cartilage: present (0); absent (1).

648. Suprascapula: large (0); small (1).

649. Scapula, emargination on anterodorsal edge (scapular fenestra): absent (0); present (1).

650. Scapulocoracoid emargination: absent (0); present (1).

651. Scapulocoracoid emargination: closed by cartilage (0); open (1); closed by scapula and coracoid (2).

652. Coracoid, anterior (primary) emargination (fenestra): absent (0); present (1).

653. Coracoid, posterior (secondary) emargination (fenestra): absent (0); present (1).

654. Coracoid size: enlarged, extending anteriorly to level of clavicles (0); not enlarged, not extending anteriorly to level of clavicles (1).

655. Epicoracoid cartilage extent: contacts mesoscapula and suprascapula (0); does not contact mesoscapula and suprascapula (1).

656. Clavicle: present (0); absent (1).

657. Clavicle: no notch or fenestration present (0); notch present (1); fenestration present (2).

658. Clavicle: rod-like (0); greatly expanded proximally (1).

659. Clavicular angulation: simple curved rod, following contour of scapulocoracoid (0); strongly angulated, curving anteriorly away from scapulocoracoid (1).

660. Distal clavicle articulation: with scapula (0); with suprascapula (1); no distal articulation (2).

661. Clavicles, medial contact: clavicles do not meet on midline (0); clavicles meet on midline (1).

662. Interclavicle: present (0); absent (1).

663. Interclavicle lateral process: present (0); absent (1).

664. Interclavicle anterior process (extending beyond lateral process): absent (0); present (1).

665. Interclavicle anterior process, length (as ratio of interclavicle length): 0.01–0.20 (0); more than 0.20 (1).

666. Interclavicle, anterior end: ventral to clavicles (0); dorsal to clavicles (1); abuts clavicles (2); lies posterior to clavicles (3).

667. Pubis: present (0); absent (1).

668. Pubis, symphyseal process orientation in ventral view: medially directed (0); anteromedially directed (1).

669. Pubis, symphyseal process: thick (0); thin (1).

670. Pubis, symphyseal process, distal breadth: expanded distally (0); tapered, not expanded distally (1); tapered, disconnected distally (2).

671. Pectineal (pubic) tubercle: closer to acetabulum than to symphysis (0); closer to symphysis than to acetabulum (or equal distance) (1).

672. Pubis, tubercle orientation in ventral view: anteriorly directed (0); ventrally directed (1).

673. Ischium: present (0); absent (1).

674. Ischial tubercle: present (0); absent, or continuous with hypoischial cartilage (1).

675. Hypoischium: well developed (expanded at distal end) (0); vestigial (no expansion at distal end) (1); absent (2).

676. Hypoischial foramen: absent (0); present (1).

677. Ilium: present (0); absent (1).

678. Ilium, tubercle: present (0); absent (1).

679. Ilium, blade orientation: slopes posterodorsally (0); oriented vertically (1); oriented anteriorly (2).

680. Ilium, dorsal ends blades: not compressed laterally, and do not expand or converge dorsomedially (0); compressed laterally, suprailiac cartilages expanded into triangular plates that converge dorsomedially (1).

681. Pelvic elements (i.e., ilium, ischium, pubis), nature of contact: in close sutural contact throughout postnatal ontogeny and co-ossified into a single pelvic bone late in postnatal ontogeny (0); distinct elements weakly united in non-sutural contacts (1).

682. Hyperischium: present (0); absent (1).

683. Hyperischial foramen: absent (0); present (1).

684. Epiphyses on long bones with separate ossification centers: present (0); absent (1).

685. Proximal forelimb long bones (humerus, radius and ulna): present (0); absent (1).

686. Ratio of radius/ulna to humerus: 0.50–0.61 (0); 0.62–0.97 (1); 0.98–1.10 (2).

687. Ectepicondylar foramen: present (0); absent (1).

688. Ulnar patella: present (0); absent (1).

689. Ulna, development of olecranon process on proximal epiphysis: prominent (0); short or absent (1).

690. Ulna, enlarged distal epiphysis that is nearly hemispherical in profile and fits into a concomitantly enlarged depression on the ulnare: absent (0); present (1).

691. Radius, styloid process: absent (0); present on posterolateral surface of distal epiphysis (1).

692. Carpal intermedium: large (0); medium small (1); small (2); tiny (3); absent (4).

693. Ball and socket joints in wrist and ankle: absent (0); present formed by large central carpal or lateral centrale (ball) and radiale, ulnare and pisiform (socket) and by distal tarsal 4 (ball) and astragalocalcaneum (socket) (1).

694. Lateral centrale in hand: separated from second distal carpal (0); contacting second distal carpal (1).

695. Proximal end of first metacarpal: separated from medial centrale (0); contacting medial centrale (1).

696. Palmar sesamoid: absent (0); present (1).

697. Metacarpals and metatarsals II to IV: longer than proximal phalanges (0); shorter than proximal phalanges (1).

698. Metacarpals, sesamoids ventral to distal heads: absent (0); present (1).

699. Phalangeal count, reduction in manus digits II to IV: three, four, five (0); reduced to three in digits III and IV (1); reduced to four in digit IV (2); reduced to three in digit III and four in digit IV (3).

700. Phalangeal count, digit V of manus: three (0); two (1); four (2).

701. Hyperphalangy in manus: absent (0); present in more than one digit (1); present only in digit 1 (2); present only in digit 5 (3).

702. Opposing digits: digits nonopposing (0); digits 1–3 opposing digits 4–5 in manus and digits 1–2 opposing digits 3–5 in pes (1).

703. Penultimate phalanges in hand: shorter than or equal to antepenultimate (0); longer than antepenultimate (1).

704. Sesamoids dorsal to distal heads of penultimate phalanges (manus): present (0); absent (1).

705. Femur: present (0); absent (1).

706. Femur: curved in dorsoventral plane (0); not curved in dorsoventral plane (1).

707. **Femur, internal trochanter:** well developed as a prominent, distinct head (0); poorly developed or absent (1).

708. Tibial patella: present (0); absent (1).

709. Tibial lunula: present (0); absent (1).

710. Fibular lunula: present (0); absent (1).

711. Dorsal and ventral tibiofemoral lunulae: both present and separate (0); ventral present, dorsal absent (1); both absent (2); both present and fused (3); dorsal present, ventral absent (4).

712. Tibia, notching of distal epiphysis: notch not present, epiphysis gently convex for astragalocalcaneal articulation (0); distinct notch present, fitting onto a ridge on the astragalocalcaneum (1).

713. Fibulo-astragalar joint: occupies less than half of distal end of fibula (0); involves most of distal end of fibula (1).

714. Tibia and fibula: widely separated at distal ends (0); very close or in contact at distal ends (1).

715. Third distal tarsal: present (0); absent (1).

716. Second distal tarsal: present (0); absent (1).

717. Astragalus and calcaneum: fused with no suture visible in adult (0); co-ossified with suture visible (1).

718. Sesamoid between metatarsal I and astragalocalcaneum (ventrally): present (0); absent (1).

719. Metatarsal V: hooked (0); broad proximally, but not hooked (1).

720. Metatarsals, sesamoids ventral to distal heads: absent (0); present (1).

721. Phalangeal counts, reduction in pes: two, three, four, five, four (0); reduced to three phalanges in digits III, IV and V (1); reduced to four phalanges in digit IV and three

phalanges in digit V (2); reduced to two phalanges in digit V (3); reduced to three phalanges in digit V (4).

722. Hyperphalangy in digits of pes: absent (0); present in more than one digit (1); present only in digit V (2).

723. Sesamoids dorsal to distal heads of penultimate phalanges: present (0); absent (1).

724. Osteoderms on body (and/or tail): not imbricate (0); imbricate, with gliding surface anteriorly (1); imbricate anteroposteriorly, but interdigitate laterally (2)

725. Imbricate body osteoderm overlap pattern: overlap primarily anteroposteriorly, slightly laterally (0); anteroposteriorly and deeply laterally (1).

726. Osteoderm ornamentation: vermiculate or smooth (0); tuberculate (1).

727. Dermal skull bone ornamentation: smooth (0); lightly rugose about frontoparietal suture (1); present over dorsum (2); present on jugal postorbital bar (3).

728. Palpebral osteoderm below supraorbital scales (and their osteoderms): absent (0); present (1).

729. Supracilliary osteoderm (pierced vertically by foramina)**:** absent (0); present (1).

730. Supracilliary osteoderm contacts parietal: present (0); absent (1).

731. Osteoderms inside supraorbital scales: absent (0); single (1); compound (2).

732. Supraorbital osteoderms inserting into sulcus along frontal supraorbital margin: absent (0); present (1).

733. Osteoderms in cheek scales: absent (0); single (1); compound (2).

734. Osteoderms in gular scales: absent (0); single (1); compound (2).

735. Osteoderms in dorsal scales: absent (0); single (1); compound (2).

736. Osteoderms in ventral scales: absent (0); single (1); compound (2).

737. Osteoderms in skull roof scales: single (0); compound (1).

738. Osteoderms in imbricate caudal scales: absent (0); present (1).

739. Mineralised cranial scales hinges: absent (0); present (1).

740. Scleral ossicle count: 16 or more (0); 14–15 (1); 12–13 (2); 11 or fewer (3).

741. Scleral ossicles: complex and irregular in shape (0); square or rectangular in shape (1).

742. Interorbital septum: present (0); absent (1).

743. Statolithic masses: absent (0); present (1).

744. Calcified endolymph: absent (0); present, but confined to occiput (1); present, extends posteriorly into neck (2).

745. Foretongue retracts into hind tongue: absent (0); present (1); tongue can be retracted entirely into buccal cavity below larynx (2).

746. Tongue tip notching, as percentage of tongue length**:** no notch (0); less than 10% (1); 10–20% (2); 20–40% (3); more than 45% (4).

747. Tongue papilla crenellated: continuous smooth distal edges of papilla (0); crenellated distal edge (1).

748. Tongue papilla shape: long, filamentous, and densely packed papilla (0); shorter, larger, somewhat compressed and tipped posteriorly (scale-like papilla) (1); deeply imbricate flat scales (2).

749. Hindtongue epithelium: discrete papilla (filamentous or scale-like) (0); transverse plicae confined to lateral margins of posterior lobes (1); transverse plicae extend across hindtongue (2); and into the anterior half of the tongue (3).

750. Infralingual folds: absent (0); present (1).

751. Papilla on ventrolateral margins of entire tongue: papillose (0); plicate (transverse scale rows) (1).

752. Tongue papilla arrangement: not in oblique rows (0); arranged in regular oblique rows (1).

753. Hypoglossal muscle: paired and smooth ventrally (0); multiple, and with fine transverse grooves beneath each muscle bundle (1).

754. Facial tongue wiping (tongue acts as an accessory eyelid): absent (0); present (1).

755. Foretongue surface: papillose (0); smooth (1).

756. Foretongue filamentous epithelium anterior extent: extends to tongue tip as long filaments (0); those overlying chemosensory part of tongue are depressed to varying degree (1).

757. Arrowhead tongue tip: tongue lateral margins continuous at tip (0); notched just behind tip (1).

758. Tongue width across posterior notch/maximum tongue length: 50–60% (0); 40–44% (1); 30–35% (2); 22–25% (3); less than 12% (4).

759. Hind tongue papilla: not sharply pointed (0); sharply pointed (1).

760. Prey prehension: crickets (or larger animals) taken primarily with tongue (0); primarily with jaws (1).

761. Amniote penis: absent (0); hemipenis present (1).

762. Hemipenis mineralisations: absent (0); comb-like (1); sleeve-like (2); spine-like (3).

763. GKMRB 607 Rectus abdominis muscles: not attached to belly skin (0); attached to hinges between ventral transverse scale rows (1).

764. Ulnar nerve pathway: superficial to limb muscles (0); deep to limb muscles (1).

765. Dorsal shank muscle innervation: peroneal nerve (0); interosseous nerve (1).

766. Ovipary vs. ovovivipary/vivipary: ovipary (0); ovovivipary to vivipary (1).

767. Ectopterygoid, extent of anterior end: restricted to posteromedial edge of maxilla (0); invades the dorsal surface of the maxilla (1).

768. Pterygoid, orientation of anterior palatine articular facet: mediolaterally expanded articular facet (0); dorsoventrally oriented articular facet (1).

769. Basipterygoid fossa on the pterygoid: present, clearly excavated to receive the basipterygoid process (0); absent (1).

770. Otooccipital, contact between distal portion of the paroccipital process and quadrate: present, contact below the supratemporal (0); absent, paroccipital process does not contact the quadrate (1).

771. Maxilla, development of posterior border of the palatine process: inconspicuous or rounded (convex), not projecting posteromedially (0); concave or notched and forming a posteromedially directed finger-like process (1).

772. Zygosphene articular facet, position with respect with the prezygapophyseal articulation surface: not distinctly projecting above the prezygapophyseal articulation in lateral view (0); distinctly projecting above the prezygapophyseal articulation in lateral view (1).

773. Basal plate: present (0); absent (1).

774. Nares, posterior elongation: absent (0); present, extending between prefrontal and nasal to reach the margin of the frontal (1).

775. Optic fenestra formed by the frontal, parietal and parasphenoid (Underwood, 1967): absent (0); present (1).

776. Spines on the hemipenial body: absent (0); present (1).

777. Neural spine, shape and length: robust posteriorly expanding lamina that does not invade the tectum of the zygosphene (0); formed by a thin, anteroposteriorly uniform lamina that invades the tectum of the zygosphene (1).

778. Pubis, orientation: dorsoventrally or horizontally directed (0); anterodorsally directed (1).

779. Caudal vertebrae, posteroventral projections: with chevron bones (caudal haemal arch) (0); with haemapophyses (1); smooth (no processes) (2).

780. Posterolateral corners of the basisphenoid: strongly ventrolaterally projected (well-developed sphenoccipital tubercle) (0); not projected (1).

781. Coronoid bone, posterior extent: contributes to the anterior margin of the adductor fossa (0); does not reach the anterior margin of the adductor fossa (1).

782. Facial carotid artery, position relative to the mandibular branch of the trigeminal nerve (V3): passes ventral to V3 (0); passes dorsal to V3 (1).

783. Muscle adductor mandibulae externus temporalis (AMET sensu Zaher, 1994a): absent (0); present (1).

784. Muscle adductor mandibulae externus medialis pars anterior (sensu Zaher, 1994a): present (0); absent (1).

785. First ceratobranchial, disposition of the cornua (Groombridge, 1979): divergent cornua (0); parallel cornua (1).

3. Supplementary Note 3: Characters used in the phylogenetic analysis 2.

Characters 1–602 taken from Zaher et al.,[13] and characters 603–656 from Garberoglio et al.[14].

The last character state of each character is followed by the citation of the original author.

The citations are abbreviated as follows: Z, Zaher et al.,[13]; G, Garberoglio et al.[14]; associated numeral with each abbreviation indicates the character number given by the original author.

Characters 1–592 are the same as those in Supplementary Note 2 and hence not repeated here.

The remaining characters are as follows:

593. Ectopterygoid, extent of anterior end: restricted to posteromedial edge of maxilla (0); invades the dorsal surface of the maxilla (1). [Z767]

594. Pterygoid, orientation of anterior palatine articular facet: mediolaterally expanded articular facet (0); dorsoventrally oriented articular facet (1). [Z768]

595. Basipterygoid fossa on the pterygoid: present, clearly excavated to receive the basipterygoid process (0); absent (1). [769]

596. Otooccipital, contact between distal portion of the paroccipital process and quadrate: present, contact below the supratemporal (0); absent, paroccipital process does not contact the quadrate (1). [Z770]

597. Maxilla, development of posterior border of the palatine process: inconspicuous or rounded (convex), not projecting posteromedially (0); concave or notched and forming a posteromedially directed finger-like process (1). [Z771]

598. Basal plate: present (0); absent (1). [Z773]

599. Nares, posterior elongation: absent (0); present, extending between prefrontal and nasal to reach the margin of the frontal (1). [Z774]

600. Optic fenestra formed by the frontal, parietal and parasphenoid (Underwood, 1967): absent (0); present (1). [Z775]

601. Posterolateral corners of the basisphenoid: strongly ventrolaterally projected (well-developed sphenoccipital tubercle) (0); not projected (1). [Z780]

602. Coronoid bone, posterior extent: contributes to the anterior margin of the adductor fossa (0); does not reach the anterior margin of the adductor fossa (1). [Z781]

603. Chevrons: present (0); absent (1). [G204]

604. Hemapophyses: absent (0); present, short (1); present, long (2). [G205]

Najash and *Dinilysia* were rescored with "?" following Zaher et al.[13]

605. Hypapophyses, distribution: restricted to anterior-most precloacal vertebrae (0); present throughout precloacal skeleton (1). [G206]

Dinilysia was rescored with "?" and *Sanajeh* with "0" following Zaher et al.[13]

606. Para-diapophysis: confluent (0); separated into dorsal and ventral facet (1). [G207]

607. Prezygapophyseal accessory processes: absent (0); present (1). [G208]

Dinilysia was rescored with "1" following Zaher et al.[13]

608. Subcentral paralymphatic fossae on middle/posterior precloacal vertebrae: absent (0); present (1). [G209; Modified]

Modifications were introduced to increase the range of scoring of this character. *Dinilysia* was rescored with "?" following Zaher et al.[13]

609. Subcentral foramina: absent (0); present, consistently small (1); present, of variable size (2). [G210]

610. Well-developed paracotylar foramina: absent (0); present (1). [G211]

Najash was rescored with "?" and *Dinilysia* with "0" following Zaher et al.[13]

611. Ventral margin of centra: smooth (0); median prominence from cotyle to condyle (1). [G212]

612. Axis intercentrum articulation: not fused to anterior region of axis centrum (0); fused (1). [G213]

613. Neural spine height: well-developed process (0); low ridge or absent (1) [G214]

614. Posterior margin of neural arch: shallowly concave in dorsal view (0); with deep V-shaped embayment in dorsal view (1). [G215]

Sanajeh was rescored with “0” following Zaher et al.[13]

615. Cotyle shape of precloacal vertebrae: oval (0); circular (1). [G216]

616. Parazygantral foramen: absent (0); present (1). [G217]

617. Lymphapophyses: absent (0); present (1). [G218]

Dinilysia was rescored with “?” following Zaher et al.[13]

618. Lymphapophyses: three or fewer (0); three lymphapophyses and one forked rib (1); more than three lymphapophyses and one forked rib (2). [G219]

619. Sacral vertebrae: present (0); absent (1). [G220]

620. Position of synapophyses in relation to lateral edge of prezygapophyses: at same level or slightly more projected laterally (0); clearly medial to edge of prezygapophyses (1). [G221]

621. Pachyostotic vertebrae: absent (0); present (1). [G222]

622. Precloacal vertebrae number: fewer than 100 (0); more than 100 (1). [G223]

623. Caudal vertebrae number: greater than 50% of precloacal number (0); approximately 10% or less than precloacal number (1). [G224]

624. Hypapophyses of anterior precloacals: short, about 50% length of centrum (0); long, subequal to or longer than centrum (1). [G225]

Sanajeh was rescored with “?” following Zaher et al.[13]

625. Dorsolateral ridges (= parasagittal ridges) of neural arch: absent (0); present (1). [G226]

626. Vertebral centrum: narrow in ventral view (0); broad and subtriangular in shape (1); broad and square (2). [G227]

Sanajeh was rescored with “1” following Zaher et al.[13]

627. Arterial grooves: absent in neural arch (0); present (1). [G228]

628. Posterior condyle: confluent with centrum ventrally (0); distinctly separated from centrum by groove/constriction between centrum and condyle (1). [G229]

Sanajeh was rescored with "1" following Zaher et al.[13]

629. Vertebrae, width: narrow, width across zygapophyses not significantly greater than distance from prezygapophyses to postzygapophyses (0); vertebrae wide, width across zygapophyses 150% of length or more (1). [G230]

630. Zygosphene anterior margin: deeply concave anterior edge (0); shallowly concave anterior edge (1); straight or slightly sinuous anterior edge (2). [G231]

631. Zygosphene width, expressed as ratio of zygosphene width to cotyle width, in anterior view: wide, ratio close to or more than 1 (0); narrow, ratio significantly less than 1 (1). [G232]

632. Vertebrae constriction index, expressed as neural arch minimal width to total width, measured at the level of the prezygapophyseal lateral edge: slight constriction, ratio equal to or more than 0.67 (0); marked constriction, ratio less than 0.67 (1). [G233]

633. Narrow and sharp haemal keel on middle precloacal vertebrea: absent (0); present (1). [234]

634. Vertebrae, cotyle size, expressed as ratio of cotyle width to total width (measured as the interdiapophyseal width): big cotyle, ratio more than 0.5 (0); middle-sized cotyle, ratio between 0.5 and 0.3 (1); small cotyle, ratio less than 0.3 (2). [G235]

635. Small lateral ridge on precloacal vertebrae extending from the parapophyses, below lateral foramen: absent (0); present (1). [G236]

636. Unfused intercentra in precloacal vertebrae posterior to the axis: present (0); absent (1). [G237]

Najash was rescored with "?" following Zaher et al.[13]

637. Arqual ridges on middle precloacal vertebrae: absent (0); present (1) [G238]

638. Condyles of middle precloacal vertebrae, orientation: facing very dorsally, ventral edge (at most) of condyle surface exposed in ventral view (0); facing posteriorly, or posterodorsally, much of condyle surface exposed in ventral view (1). [G239]

639. Orientation of zygapophyses of middle precloacal vertebrae: steeply inclined medially, 30° or more from the horizontal (0); moderately inclined medially, between 15-30° from the horizontal (1); not inclined medially, <15° from horizontal (2). [G240]

640. Vertebrae elongation index, expressed as ratio of minimal neural arch width to centrum length: ratio <09 (0); ratio close to 1 (1); ratio >1.2 (2). [G241]

641. Neural canal shape: vaulted (0); trifoliate (1). [G242]

642. Neural canal relative size: small, cross-sectional area about half or less than that of cotyle (0); large, cross-sectional area close to that of cotyle (1). [G243]

643. Prezygapophyseal articular facet projection: long axis antero-laterally oriented (<60° from sagittal plane) (0); moderately laterally oriented, long axis diverging between 60- 80° from sagittal plane (1); laterally everted, long axis diverging 80° or more from sagittal plane (2). [G244]

644. Zygosphene facet inclination from vertical: slightly inclined, angle <25° (0); moderately inclined, angle between 25-35° (1); markedly sloping, angle >35° (2). [G245]

645. Zygosphene shape index, expressed as zygosphene width to zygosphene roof thickness ratio: thin, ratio >3.5 (0); thick, ratio <3.5 (1). [G246]

646. Zygosphene roof morphology: dorsally concave (0); dorsally convex (1); horizontal (2). [G247]

647. Parapophysis ventral margin: high, placed dorsal to the ventral margin of cotyle (0); ventrally projected, level with or below ventral margin of cotyle (1). [G248]

648. Absolute size of neural spine, expressed as neural spine height (measured from dorsal edge of zygosphene) to total height of vertebra: high, >30% (0); moderate, between 15-30% (1); low, less than 15% (2). [G249]

649. Posterior tubercle on neural spine: absent (0); present (1). [G250]

650. Lateral foramina, dorsal to subcentral ridges: absent (0); present (1). [G251]

651. Position of the dorsal margin of the diapophysis relative to the neural canal in middle/posterior precloacal vertebrae: at or below the neural canal floor (0); up to and including the dorsoventral midpoint of the neural canal (1); above the midpoint of the neural canal (2). [G252; Modified]

Modifications were introduced to increase the range of scoring of this character.

652. Posteriorly canted neural spine: absent (0); present (1). [G253]

653. Process on the posterior haemal keel: absent (0); present (1). [G254]

654. Type of process on the haemal keel: bifid (0); arrow shaped (1); triangular (2); chisel shaped (3). [G255; Modified]

Modifications were introduced to adequately cover the variation in morphology, as in taxa such as *Vasuki* the posterior process of the hemal keel is chisel shaped.

655. Fossae on dorsolateral surface of neural arch: absent (0); present (1). [G256]

656. Ribs, tuber costae: absent from ribs (0); present (1). [G257]

3. Supplementary Table 1: List of specimens for the holotype of *Vasuki indicus* gen. et sp. nov. Specimen number in articulated vertebrae is designated by roman numerals where 'I' is towards the anterior direction.

Sl no.	Material	Registration number
1	Partial anterior trunk vertebra	**IITR/VPL/SB 3102-1**
2	Nearly complete anterior trunk vertebra	**IITR/VPL/SB 3102-2**
3	Partial anterior trunk vertebra	**IITR/VPL/SB 3102-3**
4	Nearly complete mid-trunk vertebra	**IITR/VPL/SB 3102-4**
5	Complete anterior trunk vertebra	**IITR/VPL/SB 3102-5**
6	Complete posterior anterior trunk vertebra	**IITR/VPL/SB 3102-6**
7	Two articulated anterior trunk vertebrae	**IITR/VPL/SB 3102-7I–II**
8	Two articulated mid-trunk vertebrae	**IITR/VPL/SB 3102-8I–II**
9	Two articulated mid-trunk vertebrae	**IITR/VPL/SB 3102-9I–II**
10	Two articulated complete posterior anterior trunk/ mid-trunk vertebrae	**IITR/VPL/SB 3102-10I–II**
11	Three articulated mid-trunk vertebrae	**IITR/VPL/SB 3102-11I–III**
12	Partial precloacal vertebra	**IITR/VPL/SB 3102-12**
13	Partial precloacal vertebra	**IITR/VPL/SB 3102-13**
14	Partial condyle	**IITR/VPL/SB 3102-14**
15	Partial condyle	**IITR/VPL/SB 3102-15**
16	Partial condyle	**IITR/VPL/SB 3102-16**
17	Partial condyle	**IITR/VPL/SB 3102-17**
18	Partial condyle	**IITR/VPL/SB 3102-18**
19	Partial mid-trunk vertebra	**IITR/VPL/SB 3102-19**
20	Partial mid-trunk vertebra	**IITR/VPL/SB 3102-20**
21	Partial mid-trunk vertebra	**IITR/VPL/SB 3102-21**

4. Supplementary Table 2: Measurement of skeletal specimens of *Vasuki indicus* gen. et sp. nov. (IITR/VPL/SB 3102). All measurements are in mm; *c.* indicates estimated measurement; *bk.* indicates measurement of broken regions. When added to the anatomical abbreviations, the suffix α indicates angle; H indicates height, L indicates length and W indicates width.

**Reg no.**	**cL**	**coH**	**coW**	**cnH**	**cnW**	**dW**	**ncH**	**ncW**	**naW**	**nsH**	**nsL**	**pofL**	**pofW**
**3102-1**	37.5	23.9	26.8	23.7	22.3	59.1	8.5	17.6	50.3	-	7.9	16.7	13.9
**3102-2**	41.3	24.3	28	25.6	25.2	66.2	9.4	19.6	56.3	*bk* 6.6	15.1	18.9	16.2
**3102-3**	48.2	29.9	30.2	28.1	30.3	84.1	9.3	27.5	68.7	15.3	18.6	21.2	18.1
**3102-4**	59.4	37.1	46.9	33.9	41.1	118.6	13.9	42.4	88.8	*bk.* 20	21.7	30.5	26.7
**3102-5**	55.43	35.7	37.8	34.2	38.4	109.2	10.4	32.4	84.9	*bk.*12.7	15.7	27.9	19.7
**3102-6**	59.9	38.7	41.9	37.8	41.9	121.1	11.8	38.2	90.3	16.4	17.9	31.9	22.7

**Reg no.**	**poW**	**poα**	**prfL**	**prfW**	**prW**	**prα**	**tvH**	**zsfL**	**zsfW**	**zsH**	**zsW**	**zsα**
**3102-1**	66.8	12°	17.5	15.7	62.4	23°	61.4	16.9	13.6	15.3	30.1	125°
**3102-2**	73.3	16°	25.4	17.1	*c.*76	20°	69.9	23.8	15.39	17.3	32.6	128°
**3102-3**	85.5	16°	28.7	16.7	86.2	28°	90.7	22.8	20.8	23.03	41.36	125°
**3102-4**	*c.*104.3	26°	34.5	27.7	109.8	27°	~107.2	27.3	23.9	31.2	43.6	121°
**3102-5**	99.78	21°	30.8	23.8	96.9	24°	~98.1	25.9	25.4	28.1	44.1	118°
**3102-6**	102.8	26°	32.6	27.1	102.6	28°	110.02	26.1	24.8	29.7	45	124°

Abbreviations: c, centrum; co, cotyle; cn, condyle; d, diapophysis; na, neural arch; nc, neural canal; ns, neural spine; pof, postzygapophyseal facet; po, postzygapophysis; prf, prezygapophyseal facet; pr, prezygapophysis; tv, total vertebra; zsf, zygosphene facet; zs, zygosphene. Measured parameters shown in Supplementary Fig. 2.

Supplementary Table 2: continued

Reg no.	cL	coH	coW	cnH	cnW	dW	ncH	ncW	naW	nsH	nsL	pofL	pofW
**3102-7A**	~44	31.6	34.3	-	~31.8	~92.3	8.7	31.7	79.2	-	-	-	~17.2
**3102-7B**	54.3	32.9	34.9	31.8	34.3	98.2	11.7	28.3	78.7	-	-	-	-
**3102-8I**	~49.3	~34.2	49.5	-	46.6	*c*.121.5	13.5	41.1	94.6	-	-	-	-
**3102-8II**	61.8	-	50.3	38	48.9	128.2	13.6	38.2	96.2	-	-	33.6	27.2
**3102-9I**	~54.3	37.5	44.5	-	36.5	113.3	15.3	39.1	76.5	-	-	24.6	
**3102-9II**	62.7	~39	42.7	35	40.5	112.9	15.6	39.2	81.3	-	-	26.1	21
**3102-10I**	~53.5	34.7	41.1	-	40.1	110.6	11.7	38	84.5	23.4	16.5	-	-
**3102-10II**	57.4	-	41.2	34.7	41.5	112.3	13.3	38.5	90.5	23.4	16.9	30	23.9

Reg no.	poW	poα	prfL	prfW	prW	prα	tvH	zsfL	zsfW	zsH	zsW	zsα
**3102-7I**	92	-	28.8	21.8	92.5	27°	-	23.9	22.4	26	43.2	122°
**3102-7II**	-	-	~22.1	~29.9	93.4		-	-	-	-	41.6	
**3102-8I**	107.3	-	36.4	28.6	109.3	27°	-	-	28.6	31.8	-	
**3102-8II**	110.9	22°	-	-	111.4	-	-	-	-	-	48.4	-
**3102-9I**	89.8	-	19	22.8	92.1	25°	-	-	-	23.6	*c*.29.6	
**3102-9II**	*c*.97.7	23°	25.9	20	91.8	-	-	-	-	-	41.5	-
**3102-10I**	99.1	-	29.8	25.1	102	24°	103.7	25.7	25.4	29.7	42.7	139°
**3102-10II**	102.1	16°	-	-	101.2	-	108.7	-	-	-	-	-

Abbreviations: c, centrum; co, cotyle; cn, condyle; d, diapophysis; na, neural arch; nc, neural canal; ns, neural spine; pof, postzygapophyseal facet; po, postzygapophysis; prf, prezygapophyseal facet; pr, prezygapophysis; tv, total vertebra; zsf, zygosphene facet; zs, zygosphene. Measured parameters shown in Supplementary Fig. 2.

Supplementary Table 2: continued

Reg no.	cL	coH	coW	cnH	cnW	dW	ncH	ncW	naW	nsH	nsL	pofL	pofW
**3102-11I**	*bk*.45	-	-	-	*c*.45	-	11.7	37.6	-				
**3102-11II**	~45.5	-	48.8	-	~41.8	*bk*.113.6	-	-	93.2	25.6	19.4		
**3102-11III**	60.4	-	48.9	*bk*.36.5	44.1	*bk*.118	12.3	40	95.5	31.9	18.8	27.2	29.1
**3102-12**	-	30.1	33.1	-	-	91	-	-	~73.9	-	-	-	-
**3102-13**	-	33.5	*bk*.38	-	-	-	-	-	-	-	-	-	-
**3102-14**	-	-	-	36.1	41.4	-	-	-	-	-	-	-	-
**3102-15**	-	-	-	38.2	38	-	-	-	-	-	-	-	-
**3102-16**	-	-	-	37.1	41.9	-	-	-	-	-	-	-	-
**3102-17**	-	-	-	35.7	38.9	-	-	-	-	-	-	-	-
**3102-18**	-	-	-	*bk*.20	27.8	-	-	-	-	-	-	-	-
**3102-19**	71.2	-	47.3	35.2	43.7	-	14.8	41.5	-	-	-	-	-
**3102-20**	57.4	-	29.9	26.3	27.2	-	-	-	63.2	-	-	-	-
**3102-21**	72.4	35.3	-	-	-	-	-	-	-	-	-	-	-

Reg no.	poW	poα	prfL	prfW	prW	prα	tvH	zsfL	zsfW	zsH	zsW	zsα
**3102-11I**	*bk*.104	-	-	-		-	-	-	-	-	-	-
**3102-11II**	*bk*.111.6	-	-	22.5	106.7	-	107.2	-	-	-	-	-
**3102-11III**	*bk*.95.9	-	-	22.9	109.6	-	109.6	-	-	-	-	-
**3102-12**	-	-	27.9	20.8	90.5	26°	-	-	-	-	-	-
**3102-13**	-	-	~25.6	23.9	-	~20°	-	-	-	-	-	-
**3102-19**	-	-	31.1	27.7	-	25°	-	-	23.5	-	-	-
**3102-20**	-	-	14.1	16.3	79.6	20°	-	-	17	-	36.6	-

Abbreviations: c, centrum; co, cotyle; cn, condyle; d, diapophysis; na, neural arch; nc, neural canal; ns, neural spine; pof, postzygapophyseal facet; po, postzygapophysis; prf, prezygapophyseal facet; pr, prezygapophysis; tv, total vertebra; zsf, zygosphene facet; zs, zygosphene. Measured parameters shown in Supplementary Fig. 2.

5. Supplementary Table 3: Measurements of extant boine snakes from Head et al.[15] used in body length estimations. The data are from vertebrae 60% posteriorly along the vertebral column. All measurements are in mm.

Sl no.	Extant taxa	Specimen	TBL	poW
1	*Acrantophis dumerili*	USNM 497683	1535	6.38
2	*Acrantophis dumerili*	ROMV-R 7864	2040	19.66
3	*Acrantophis dumerili*	ROMV-R 7833	2480	23.5
4	*Boa constrictor*	USNM-348597	1606	12.92
5	*Boa constrictor*	ROMV-R 7182	3220	27.69
6	*Boa constrictor*	USNM 220299	3434	28.2
7	*Candoia carinata*	USNM 348502	867	11.22
8	*Corallus caninus*	ROMV-R7498	1450	12.2
9	*Corallus enhydris*	ROMV-R 4075	1734	10.79
10	*Epicrates anguilifer*	ROMV-R 7842	2330	17.61
11	*Epicrates cenchria*	ROMV-R 7902	1380	11.54
12	*Epicrates cenchria*	ROMV-R 5345	1210	10.65
13	*Epicrates inornatus*	ROMV-R 7900	1700	11.66
14	*Epicrates striatus*	ROMV-R 7901	1770	11.2
15	*Epicrates striatus*	UF63866	2250	15.09
16	*Epicrates subflavus*	UF69268	1720	10.8
17	*Eunectes murinus*	ROMV-R 7340	2470	17.1
18	*Eunectes murinus*	ROMV-R 7285	3320	24.32
19	*Eunectes notaeus*	ROMV-R 7307	2510	17.67
20	*Eunectes notaeus*	ROMV-R 7286	2690	24.33
21	*Sanzinia madagascarensis*	USNM 220313	1760	16.15

Anatomical abbreviations: poW, postzygapophyseal width; TBL, total body length.

Institutional abbreviations: UF, University of Florida; ROMV-R, Royal Ontario Museum, Recent Collection; USNM, United States National Museum, Smithsonian Institution.

6. Supplementary Table 4: Measurements of extant snakes from Head et al.[15] used in body length estimations. The data are from vertebrae 65% posteriorly along the vertebral column. All measurements are in mm.

Sl no.	Extant taxa	Specimen	TBL	poW
1	*Acrantophis dumerili*	USNM 497683	1535	6.53
2	*Acrantophis dumerili*	ROMV-R 7864	2040	19.04
3	*Acrantophis dumerili*	ROMV-R 7833	2480	21.42
4	*Boa constrictor*	USNM-348597	1606	12.64
5	*Boa constrictor*	ROMV-R 7182	3220	27.08
6	*Boa constrictor*	USNM 220299	3434	28.01
7	*Candoia carinata*	USNM 348502	867	10.96
8	*Corallus caninus*	ROMV-R7498	1450	11.8
9	*Corallus enhydris*	ROMV-R 4075	1734	10.54
10	*Epicrates anguilifer*	ROMV-R 7842	2330	17.97
11	*Epicrates cenchria*	ROMV-R 7902	1380	11.27
12	*Epicrates cenchria*	ROMV-R 5345	1210	10.22
13	*Epicrates inornatus*	ROMV-R 7900	1700	11.58
14	*Epicrates striatus*	ROMV-R 7901	1770	10.85
15	*Epicrates striatus*	UF63866	2250	15.08
16	*Epicrates subflavus*	UF69268	1720	10.66
17	*Eunectes murinus*	ROMV-R 7340	2470	17.26
18	*Eunectes murinus*	ROMV-R 7285	3320	23.99
19	*Eunectes notaeus*	ROMV-R 7307	2510	17.24
20	*Eunectes notaeus*	ROMV-R 7286	2690	23.12
21	*Sanzinia madagascarensis*	USNM 220313	1760	15.69

Anatomical abbreviations: poW, postzygapophyseal width; TBL, total body length.

Institutional abbreviations: UF, University of Florida; ROMV-R, Royal Ontario Museum, Recent Collection; USNM, United States National Museum, Smithsonian Institution

7. Supplementary Table 5: Measurements of extant snakes from McCartney et al.[16] used in body length estimations. All measurements are in mm.

Sl no.	Extant taxa	Specimen	TBL	prW
1	*Afrotyphlops punctatus*	NJK Tpl 05-1	567	4.02
2	*Boa constrictor*	YPM R12323	2382.1	21.63
3	*Eunectes murinus*	CM 145331	4267	40.42
4	*Eryx colubrinus*	NJK NN	520	5.5
5	*Eryx miliarius*	NJK Em 05-1	416	4.06
6	*Drymarchon couperi*	YPM R10715	1905	16.71
7	*Gyalopion canum*	NJK Gc 05-1	266	2.81
8	*Phyllorhynchus browni*	NJK Pb 05-1	342	3.25
9	*Naja naja*	CM 37154	1520	9.95
10	*Ophiophagus hannah*	YPM R17287	3403	21.02
11	*Loxocemus bicolor*	NJK lb07-D2	940	6.6
12	*Malayopython reticulatus*	AMNH 77082	6165	42.69
13	*Malayopython reticulatus*	AMNH R144528	6197.5	45.41
14	*Malayopython reticulatus*	AMNH R144529	5615	45.62
15	*Malayopython reticulatus*	CM 30467	6705	44.69
16	*Python bivitattus*	UMA R 1363	5384	43.47
17	*Python brongersmai*	NJK Pb08-1	1650	23
18	*Python molurus*	YPM R12545	3813	29.14
19	*Python regius*	AMNH 137178	755	9.21
20	*Python sebae*	CM 33987	3911	28.53
21	*Tropidophis haetianus*	NJK Th 06-2	535	5.43

Anatomical abbreviations: prW, prezygapophyseal width; TBL, total body length.

Institutional abbreviations: CM, Carnegie Museum of Natural History, Pittsburgh, PA, U.S.A.; NJK, specimens housed in collections at Stony Brook University, Stony Brook, NY, U.S.A.; UA, Université d’Antananarivo, Antananarivo, Madagascar; YPM, Yale Peabody Museum of Natural History, New Haven, CT, U.S.A.

Supplementary Figures

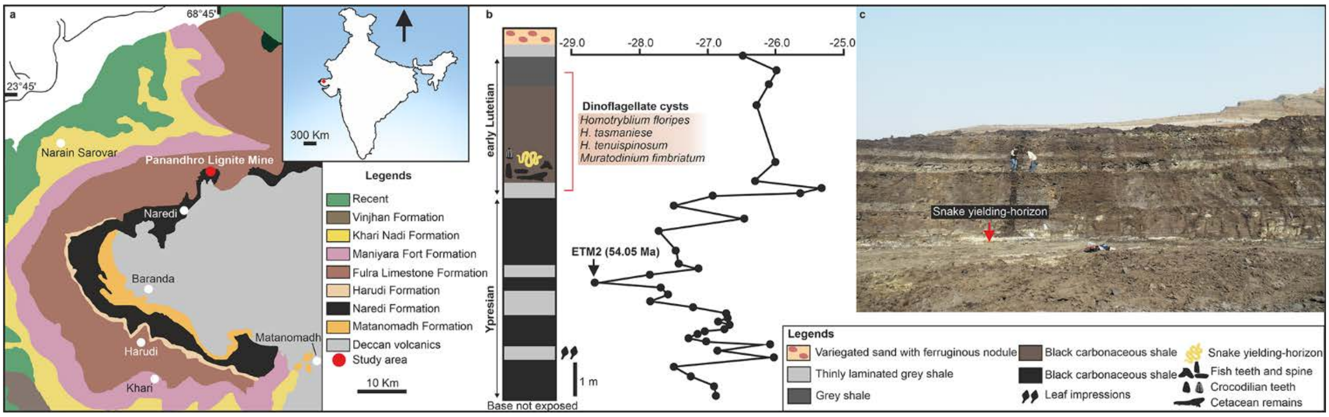

Figure 1. Geological map of Kutch on-land basin showing fossil locality (a); stratigraphic column at Panandhro Lignite Mine showing the position of madtsoiid snake-yielding horizon with age diagnostic dinoflagellate cyst assemblage and $\delta^{13}C$ curve marking hyperthermal event ETM2 (modified after Agrawal et al.[5]) (b); panoramic view of the fossil site. Map and stratigraphic column were drawn by D.D. using CorelDRAW 2019 (Version number: 21.0.0.593, URL link: http:// www. corel.com/ en/). ETM2 age estimate after Westerhold et al.[16].

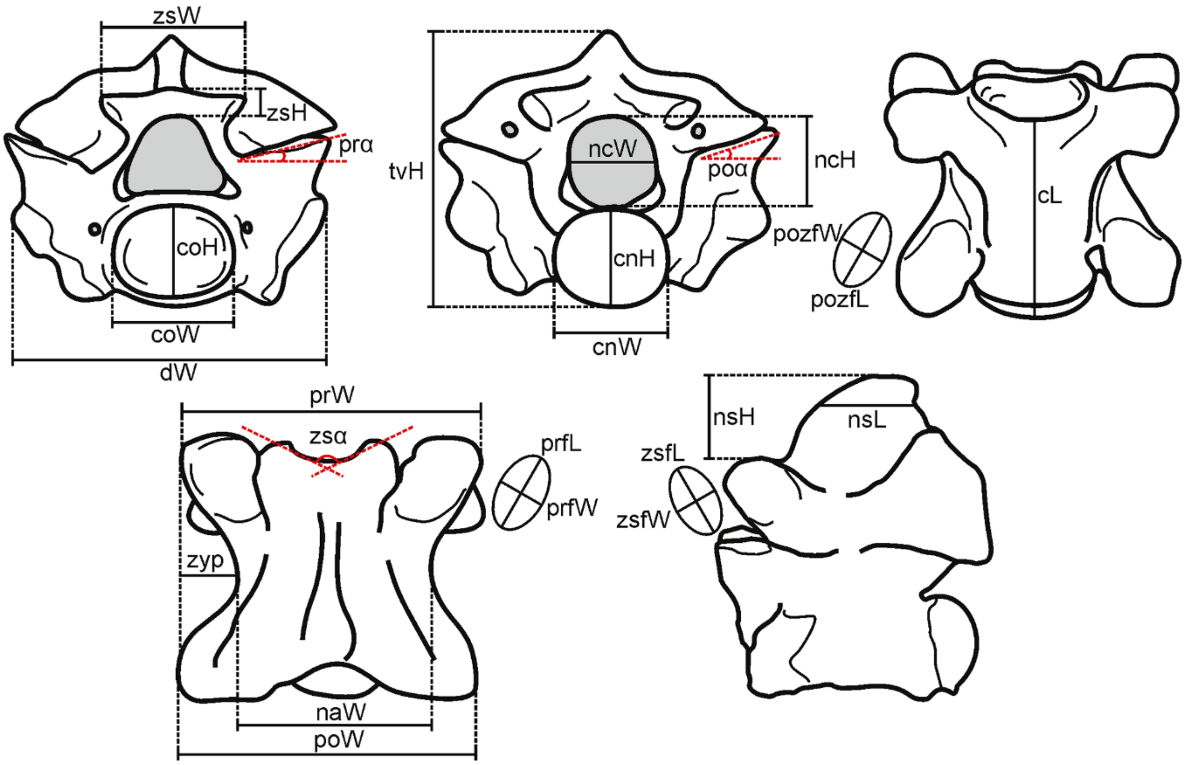

Supplementary Fig. 2. Index of measured parameters shown on the schematic representation of a basic snake vertebral morphology in: anterior (a) and lateral (b) views. When added to the anatomical abbreviations, the suffix α indicates angle; H indicates height, L indicates length and W indicates width. Abbreviations: c, centrum; co, cotyle; cn, condyle; na, neural arch; nc, neural canal; ns, neural spine; pof, postzygapophyseal facet; po, postzygapophysis; prz, prezygapophysis; prf, prezygapophyseal facet; tv, total vertebra; zsf, zigosphene facet; zs, zygosphene. Schematic diagrams not to scale and after McCartney and Seiffert[17].

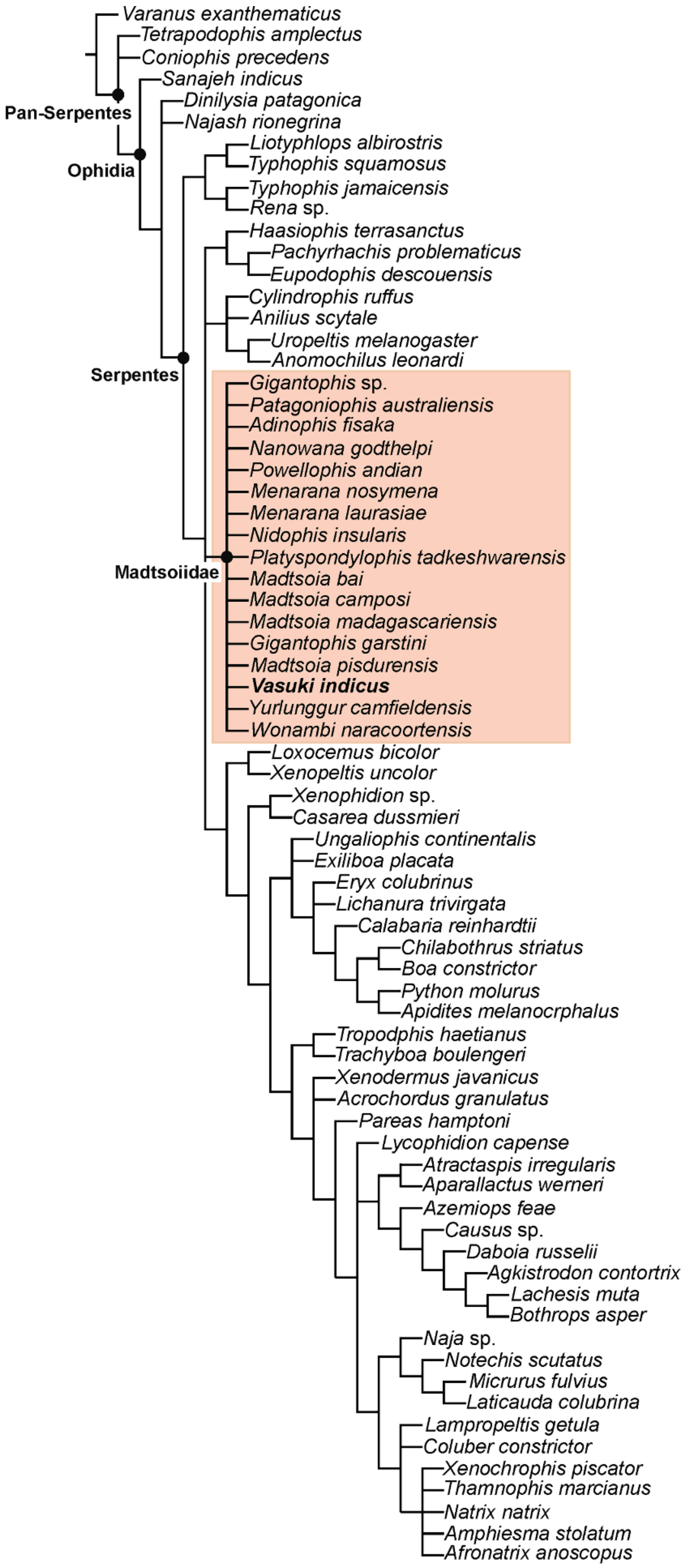

Supplementary Fig. 3. Phylogenetic position of *Vasuki indicus* gen. et sp. nov. IITR/VPL/SB 3102 in strict consensus tree of Analysis 1. Clade comprising Vasuki indicus highlighted in pink.

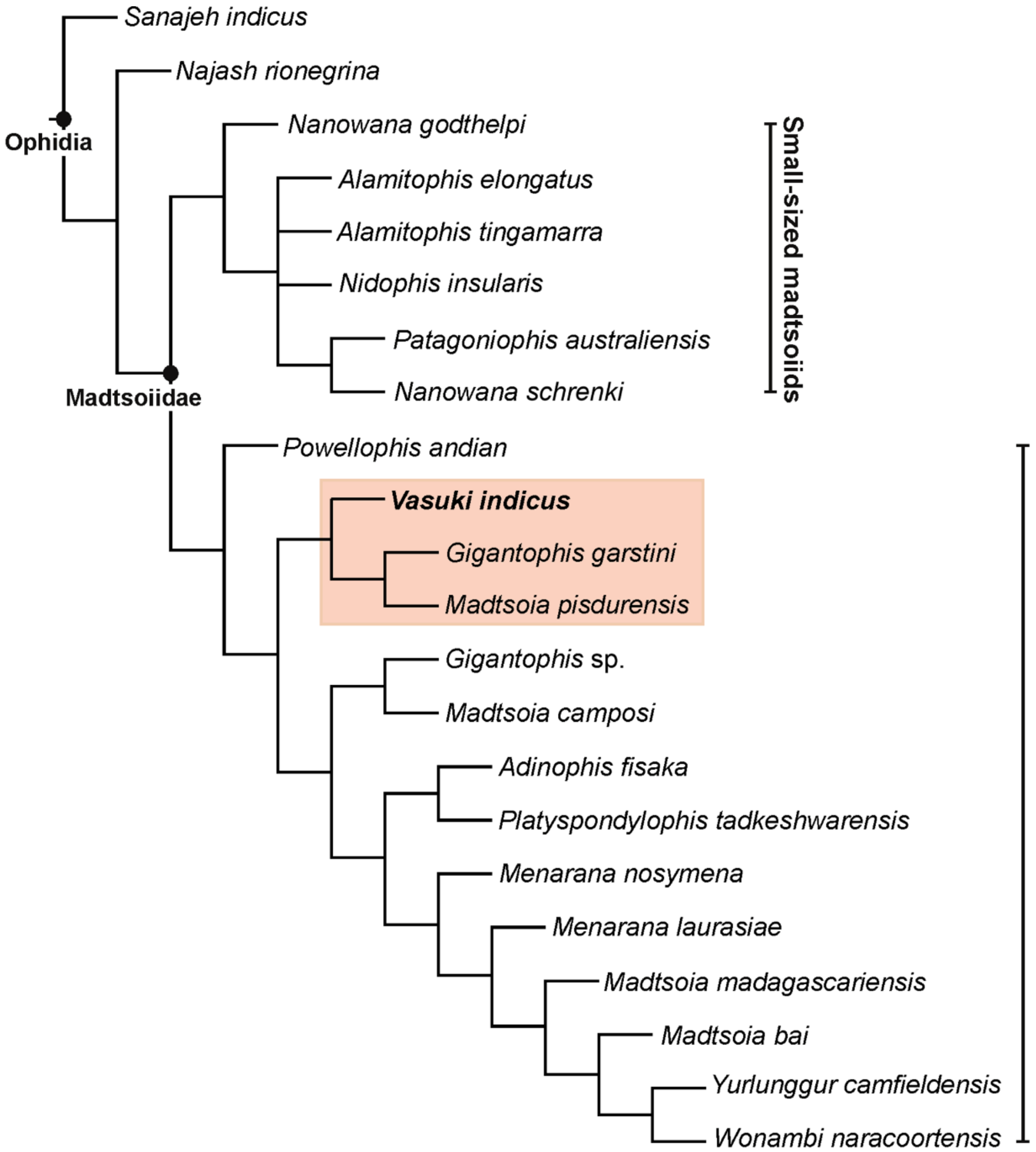

Supplementary Fig. 4. Phylogenetic position of *Vasuki indicus* gen. et sp. nov. IITR/VPL/SB 3102 in strict consensus tree of Analysis 2. Clade comprising Vasuki indicus highlighted in pink.

Supplementary References

1. Biswas, S.K. Tertiary stratigraphy of Kutch. *J. Palaeontol. Soc. India,* **37**, 1–29 (1992).
2. Khanolkar, S., Saraswati, P. K. & Rogers, K. Ecology of foraminifera during the middle Eocene climatic optimum in Kutch, India. *Geodin. Acta*. **29**, 181–193 (2017).
3. Khanolkar, S. & Saraswati, P. K. Eocene foraminiferal biofacies in Kutch Basin (India) in context of palaeoclimate and palaeoecology. *J. Palaeogeogr*. **8**, 1–16 (2019).
4. Biswas, S. K. Regional tectonic framework, structure and evolution of the western marginal basins of India. *Tectonophysics,* **135**, 307–327 (1987).
5. Agrawal, S. *et al*. Lignite deposits of the Kutch Basin, western India: Carbon isotopic and palynological signatures of the early Eocene hyperthermal event ETM2. J. Asian Earth Sci. 146, 296–303 (2017).
6. Mukhopadhyay, S. & Shome, S. Depositional environment and basin development during early Palaeogene Lignite Deposition, Western Kutch, Gujarat. *J. Geol. Soc. India*, **47**, 579–592 (1996).
7. Bajpai, S. & Thewissen, J. G. M. Vertebrate fauna from Panandhro lignite field (Lower Eocene), District Kachchh, western India. *Curr. Sci*. **82**, 507–509 (2002).
8. Rage, J.-C. et al. Early Eocene snakes from Kutch, Western India, with a review of the Palaeophiidae. Geodiversitas, 25, 695–716 (2003).
9. Thewissen, J. G. M. & Bajpai, S. New skeletal material of *Andrewsiphius* and *Kutchicetus*, two Eocene cetaceans from India. *J. Paleontol*. **83**, 635–663 (2009).
10. Saraswati, P. K. & Banerjee, R. K. Lithostratigraphic classification of the Tertiary sequence of northwestern Kutch. In: Proc. X Indian Colloq. *Micropalaeont. Stratigr*., Pune, 369–376 (1984).

11. Saraswati, P. K. *et al*. Foraminiferal biostratigraphy of lignite mines of Kutch, India, age of lignite and fossil vertebrates. *J. Palaeogeogr*. **3**, 90–98 (2014).

12. Sharma, J. & Saraswati, P. K. Lignites of Kutch, western India: Dinoflagellate biostratigraphy and palaeoclimate. *Rev. Micropaleontol.* **58**, 107–119 (2015).

13. Zaher, H., Mohabey, D. M., Grazziotin, F. G. & Wilson Mantilla, J. A. The skull of Sanajeh indicus, a Cretaceous snake with an upper temporal bar, and the origin of ophidian wide-gaped feeding. *Zool. J. Linn. Soc.* **197**, 656–697 (2023).

14. Garberoglio, F. F., Triviño, L. N. & Albino, A. A new madtsoiid snake from the Paleogene of South America (northwestern Argentina), based on an articulated postcranial skeleton. *J. Vertebr. Paleontol.* 42, e2128687 (2022).

15. Head, J. J. *et al*. Giant boid snake from the Palaeocene neotropics reveals hotter past equatorial temperatures. *Nature*, **457**, 715–717 (2009).

16. McCartney, J. A., Roberts, E. M., Tapanila, L. & Oleary, M. A. Large palaeophiid and nigerophiid snakes from Paleogene Trans-Saharan Seaway deposits of Mali. Acta Palaeontol. Pol. 63, 207–220 (2018).

17. Westerhold, T., Röhl, U., Donner, B. & Zachos, J. C. Global extent of early Eocene hyperthermal events: A new Pacific benthic foraminiferal isotope record from Shatsky Rise (ODP Site 1209). Paleoceanogr. Paleoclimatol. 33, 626–642 (2018).

18. McCartney, J. A. & Seiffert, E. R. A late Eocene snake fauna from the Fayum Depression, Egypt. J. Vertebr. Paleontol. 36, e1029580 (2016).
